# Deep learning analysis of soybean cyst nematode effectors to proven soybean resistance genes and homolog identification in the sugar beet-sugar beet root maggot plant pathosystem

**DOI:** 10.1016/j.dib.2026.112749

**Published:** 2026-04-11

**Authors:** Sidra S. Rahim, Yerang Lee, Nadim W. Alkharouf, Chenggen Chu, Vincent P. Klink

**Affiliations:** a Department of Computer and Information Sciences, Towson University, Towson, MD, 21252, USA; b USDA-ARS-NA- Northern Great Plains Research Laboratory, Northern Crop Science Laboratory, 1307 N 18TH ST, Fargo, ND 58102, USA; c USDA-ARS-NEA-BARC, Molecular Plant Pathology Laboratory, Building 004, Room 122, BARC-West, 10300 Baltimore Ave., Beltsville, MD 20705, USA

**Keywords:** Plant pathogen, Insect, *Tetanops myopaeformis*, Nematode, *Heterodera glycines*, Stakeholder, Trypsin, Kunitz trypsin inhibitor, Rhg1

## Abstract

Sugar beet (*Beta vulgaris* ssp. vulgaris) is one of two plants from which sugar is broadly produced, accounting for 55% of U.S. sugar ($1B, U.S.) and 35% global raw sugar ($4.6B), annually. The sugar beet root maggot (*Tetanops myopaeformis*) is its top pathogen in the U.S., and capable of causing total crop failure, making its study of urgent need. A *B. vulgaris* protease inhibitor, *Bv*STI (DV501688), one of 22 of its Kunitz trypsin inhibitors (KTIs) was shown through deep learning analyses to bind 9 different *T. myopaeformis* trypsins (Trps). As a surrogate crop experimental system to understand resistance in the *B. vulgaris-T. myopaeformis* pathosystem, transgenic analysis identified homologous *Glycine max* (soybean) KTIs expressed in root cells undergoing resistance to its most important pathogen, *Heterodera glycines* and suppresses parasitism by greater than 80%. The study adds to a list of 114 soybean genes identified in laser microdissection-assisted analyses whose expression suppresses *H. glycines* parasitism. Deep learning analyses demonstrate the predicted interaction between 2 different *H. glycines* Trps and each of the 114 defense proteins. Predicted interactions include proteins functioning in the circadian clock, pathogen activated molecular pattern (PAMP)-triggered immunity (PTI), nodulation, salicylic acid-mediated defense processes, cell wall metabolism, and vesicle transport including an alpha soluble NSF attachment protein homolog found at the Rhg1 locus. BLASTp analyses of the *B. vulgaris* proteome with all 114 *G. max* defense proteins identified the sugar beet homologs as a pool of potential candidate genes to be employed to generate durable resistance to *T. myopaeformis*.

Specifications Table

**Table:** 

Subject	Omics
Specific subject area	Genomics
Data format	Raw, Analyzed, Filtered
Type of data	Table, Figures
Data collection	
Data source location	
Data accessibility	Repository name: NCBI; The Beta vulgaris Resource, Phytozome Direct URL to data: https://www.ncbi.nlm.nih.gov/bioproject/PRJNA1026092; https://bvseq.boku.ac.at/; https://phytozome-next.jgi.doe.gov/ The data has been deposited in Genbank SRA archive found at NCBI, or is available at the other sites, meeting the requirements for submission.

## 1 Value of the Data

The availability of the *T. myopaeformis*, sugar beet root maggot (SBRM) genome, TmSBRM_v1.0, provides data researchers can use to understand its biology. The data has been used in parallel with the genome of sugar beet, of which there are hundreds of accessions that are available. Among these accessions are comparatively few that exhibit natural resistance to *T. myopaeformis*. Through this identification, the process of resistance at the genetic and molecular levels are becoming understood. The analysis has benefitted from the identification of an actual *B. vulgaris* protein that has a defence function, providing a polypeptide sequence for deep learning analyses to determine how it could inhibit *T. myopaeformis* effectors that perturb the defence response of the host. The *T. myopaeformis* genome is undergoing updates in sequence quality and annotation to improve its understanding and agronomic impact for stakeholders. The information and methods aid comparative studies as is shown here to other crop pests (i.e. *Heterodera glycines*) of *Glycine max* (soybean). This is notable because *G. max* is also an important crop, ($31B, U.S., $155B, globally, annually), and top U.S. export crop ($27B, annually), but global production is hampered by *H. glycines* with an annual loss of $1.5B, U.S., $23B, globally.

The data has been deposited in public databases or is available as supplemental data and is available freely for use.

The data is anticipated to be used scientifically. This analysis has improved the current annotations in two different crops (*B. vulgaris* and *G. max*), and two different crop pathogen species (*T. myopaeformis* and *H. glycines*). The work not only expands on knowledge of the KTI and Trp gene families, but broadens that understanding to the other gene families presented here. Notably, the presented annotation of the KTIs and other gene families permits target identification for their suppression or perturbation, or enhancement through overexpression, RNA interference (RNAi), mutagenesis, clustered regularly interspaced short palindromic repeats (CRISPR)/CRISPR-associated protein 9 (Cas9) (CRISPR/Cas9)-mediated gene editing, or technology-based traditional breeding [[1], [2], [3], [4], [5], [6], [7], [8], [9], [10]]. The end goal is providing more new information for the scientific community and stakeholders and a broad context to the results presented here that, otherwise, would not exist. Importantly, the analysis provides 3-dimensional protein-protein interaction maps that are built off prior information derived between the host, *B. vulgaris*, and its most significant pathogen, *T. myopaeformis*. The work presented here has used that information to provide the same type of data for the surrogate heterologous plant pathosystem *G. max* and *H. glycines* and a panel of 114 proven defence proteins used to identify homologs in *B. vulgaris* for better control measures for *T. myopaeformis*.

## 2 Background

The sugar beet root maggot (SBRM), *T. myopaeformis* (von Röder), devastates sugar beet (SB), *B. vulgaris*, ssp vulgaris (*B. vulgaris*) [11,12]. SB is an important food crop and one of only two plants, globally, from which sugar is widely produced, accounting for 35% of global raw sugar with an annual farm value in the U.S. of $1B alone [11,12]. Agricultural control of SBRM is limited by a scarcity of genetic knowledge [11]. The *de novo* sequenced and assembled *T. myopaeformis* draft genome and annotation is aiding in its understanding and generating resistance [13,14]. Transgenic analysis in sugar beet is challenging so experiments have been done in other significant crop plant systems like *G. max* to understand various developmental and/or plant disease concepts [15]. The *G. max*-*H. glycines* plant pathosystem has similarity to the *B. vulgaris*- *T. myopaeformis* system in that the pathogen affects the root, making it a useful study system [15].

## 3 Data Description

The relative transcript abundance of the *B. vulgaris* protease inhibitor, *Bv*STI (DV501688) is increased by infection by *T. myopaeformis* during a resistant reaction [16]. *Bv*STI is a Kunitz trypsin inhibitor (KTI). BLASTp analyses of the sugar beet proteome identified an additional 21 *Bv*KTIs. KTIs role is to inhibit pathogen trypsins (Trps) which facilitate infection. BLASTp searches of the *T. myopaeformis* genome made possible by using the Trp protein XP_014094233 from the dipteran *Bactrocera oleae* identified 9 Trps (i.e. *Tm*Trp-g7808 [*Tm*Trp1], *Tm*Trp-g3594 [*Tm*Trp2], *Tm*Trp-g23695 [*Tm*Trp3], *Tm*Trp-g23693 [*Tm*Trp4], *Tm*Trp-g3592 [*Tm*Trp5], *Tm*Trp-g7809 [*Tm*Trp6], *Tm*Trp-g23699 [*Tm*Trp7], *Tm*Trp-g23186 [*Tm*Trp8], and *Tm*Trp-g18287 [*Tm*Trp9]) from the publicly available annotated genome of *T. myopaeformis*, BioSample accession: SAMN37733483, BioProject ID PRJNA1026092. The data are available at the URL: https://www.ncbi.nlm.nih.gov/bioproject/PRJNA1026092. Deep learning analyses led to the identification of *Bv*KTIs that are predicted to dock to the *Tm*Trps, revealing they may interact and could function in resistance. However, for any of the computational predicted protein-protein binding outcomes, biochemical, genetic and/or in vivo work would enhance the understanding of their nature. The scope of the *Bv*KTI protein family is 22 *Bv*KTIs (*Bv*KTI1-*Bv*KTI22) in the RefBeet1.1 proteome. The analysis was repeated in the *G. max* proteome, using the *Bv*KTI1 protein sequence in BLASTp searches, identifying 35 *Gm*KTIs (*Gm*KTI1-*Gm*KTI35). Furthermore, the *H. glycines* Trps have already been identified and are available (Accessions: *Hg*Trp1 [CAA74204], and *Hg*Trp2 [CAA74205]), allowing deep learning analyses to determine interaction followed by transgenic analyses that demonstrated *GmKTI20* and *GmKTI30* overexpression suppress parasitism by over 80% while RNAi increases parasitism 7-8 fold [17]. Pertinent to this study, prior analyses identified 114 *G. max* genes whose overexpression functions in increasing their relative transcript abundance which aids *G. max* in resisting *H. glycines* parasitism between 29-87% (Supplemental data 1) [18]. Identified in this analysis were 14 proteins (12.39%) that were predicted to bind *Hg*Trp1, 43 proteins (37.71%) predicted to bind *Hg*Trp2, with 5 of them (4.42%) being common binding partners to *Hg*Trp1 and *Hg*Trp2.

Pathogen recognition receptor (PRR)-mediated defense is composed of 2 interconnected layers. One layer is the pathogen activated molecular pattern (PAMP) triggered immunity (PTI), and the second being effector triggered immunity [19]. *Hg*Trp1 was predicted to bind the ETI receptor NONRACE-SPECIFIC DISEASE RESISANCE1 (NDR1) receptor. Through NDR1, ETI activates processes leading to MAPK3 phosphorylation and inducing transcription. *Hg*Trp1 was predicted to bind NONRACE-SPECIFIC DISEASE RESISANCE1 (*Gm*NDR1-1) (Glyma.12G214100); mitogen activated protein kinase 3 (*Gm*MAPK 3-1) (Glyma.U021800), *Gm*MAPK13-1) (Glyma.12G073700), and *Gm*MAPK 20-2 (Glyma.14G028100); HMG I/Y transcription factor (Glyma.08G052600); the secretion components including conserved oligomeric complex 1 (*Gm*COG1-2) (Glyma.20G188500), *Gm*COG2-2 (Glyma.05G047300), *Gm*COG3-1 (Glyma.13G114900), coatomer epsilon (*Gm*Cϵ−1) (Glyma.09G030400), exocyst 4 (*Gm*EXOC4-1) (Glyma.10G207900), *Gm*EXOC5-2 (Glyma.16G014200), and syntaxin 6 (*Gm*SYP6-1) (Glyma.17G063600); and the secreted proteins galactose mutarotase-like protein (*Gm*GAL MUT) (Glyma.19G020700), and alpha hydroxynitrile glucosidase (*Gm*βg-4) (Glyma.11G129600) [[20], [21], [22]].

**Fig. 1:**
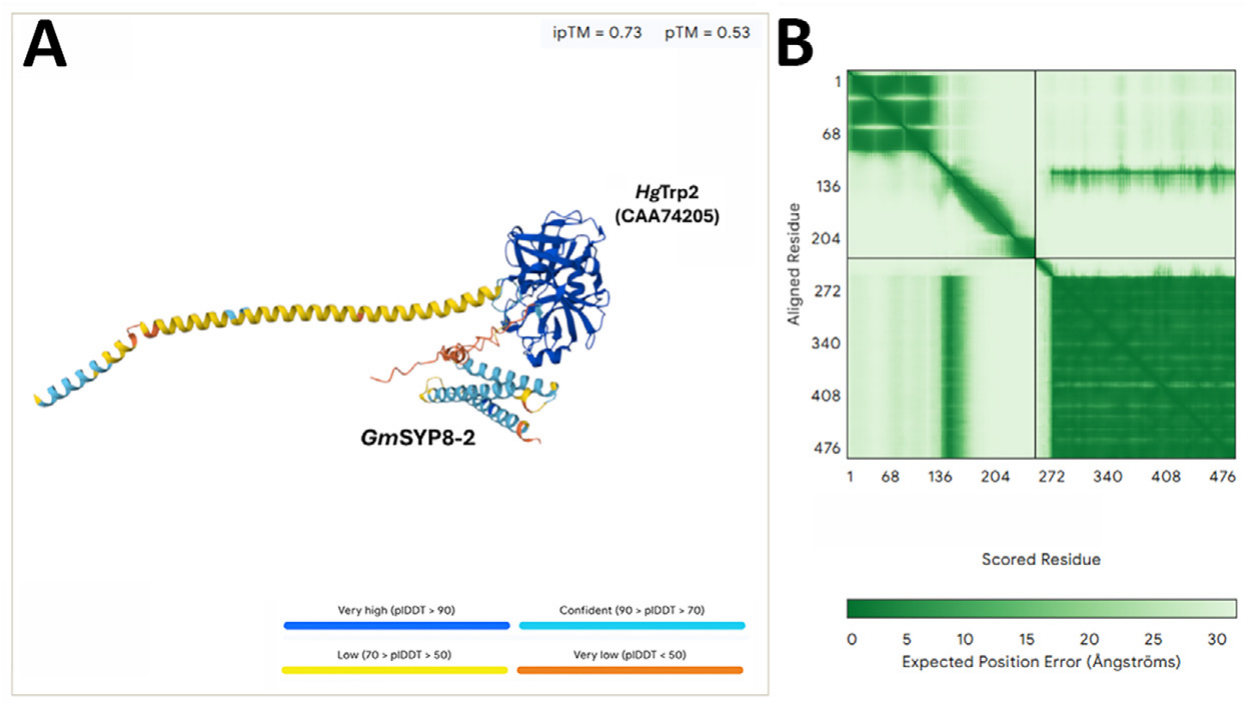
Deep learning analysis identifying interactions between *Gm*Syp8-2 and a *Hg*Trp2. **A**. Graphical view. **B**. The predicted aligned error (PAE) indicates high confidence in both the residue positioning and the overall structural model.

*Hg*Trp2 was also studied. The PTI receptor BRASSINOSTEROID INSENSITIVE 1-ASSOCIATED RECEPTOR KINASE1 (BAK1) is a co-receptor of membrane receptors that bind the PAMP that then signals defense through BOTRYTIS INDUCED KINASE 1 (BIK1) to activate downstream defense processes. *Hg*Trp2 was predicted to bind *Gm*BAK1-1 (Glyma.15G051600). Furthermore, the ETI receptor NDR1 binds proteins including RPM1-interacting protein 4 (RIN4) that regulates its downstream activity. Our analysis identified *Hg*Trp2 binds *Gm*BAK1-1 (Glyma.15G051600) *Gm*RIN4-4 (Glyma.18G166800); along with *Gm*MAPK 4-1 (Glyma.07G066800), *Gm*MAPK 16-4 (Glyma.07G255400), and *Gm*MAPK 20-2 (Glyma.14G028100); the circadian clock regulators Circadian Clock Associated 1 (*Gm*CCA1-1) (Glyma.07G048500), Timing of CAB expression 1 (*Gm*TOC1-1) (Glyma.04G166300), Gigantea (*Gm*GI-1) (Glyma.16G163200); the pathogen defense co-transcriptional activator NONEXPRESSOR OF PR1 (*Gm*NPR1) (Glyma.09G020800), Suppressor of NPR1 (*Gm*SNC1) (Glyma.16G006400); the transcription factor HMG I/Y (Glyma.08G052600); the nodulation symbiosis regulators Doesnt make infections1 (*Gm*DMI1-3) (Glyma.19G263500), *Gm*DMI3-2 (Glyma.15G222300); vesicle-associated or secreted BetV 1 pollen allergen (Bet1-1) (Glyma.06G098800), unknown secreted protein (Glyma.10G161500), secreted lipase (Glyma.08G137000), βg-4 (Glyma.11G129600), GAL MUT (Glyma.19G020700), and xyloglucan xylosy transferase (XXT 2-5) (Glyma.19G210200); proteins lacking secretion signals including heat shock protein HSP70 (Glyma.03G171100); other proteins including ascorbate peroxidase (Glyma.12G073100), aquaporin (Glyma.13G224900), and ABC transporter (ABC-G-26) (Glyma.17G039300). The analysis also identified the translocon component *Gm*Sec61-β-4 (Glyma.12G171400); secretion4 (Sec4-6) Glyma.20G099300, Sec14-1 (Glyma.14G075300), Sec23-5 (Glyma.18G003500); EXOC3-5 (Glyma.03G026900), EXOC6-1 (Glyma.02G160300), EXOC7-G1-4 (Glyma.17G197600), EXOC7-D1-2 (Glyma.16G009800); coatomer zeta (Cζ−3) (Glyma.15G008700); COG4-2 (Glyma.03G261100), COG8-1 (Glyma.16G120600); synaptosome associated protein 25kDa (SNAP25-3) (Glyma.17G076600); synaptotagmin (SYT-3) (Glyma.11G107300), synaptobrevin (SYB-2) (Glyma.16G010800); syntaxin 6 (SYP6-1) (Glyma.17G063600), SYP8-2 (Glyma.14G017700)SYP38 (Glyma.14G064300), SYP71-6 (Glyma.19G180200), SYP121-1 (Glyma.02G195300); and alpha soluble NSF attachment protein (*Gm*α-SNAP-5) (Glyma.18G022500), a protein whose homologs binds SYPs and is purported to be the major *H. glycines* resistance gene at the Rhg1 locus. An example of an *Hg*Trp2 docking is shown (Fig. 1).

Proteins predicted to bind in common between *Hg*Trp1 and *Hg*trp2 include a HMG I/Y transcription factor, βg-4, *Gm*MAPK 20-2, SYP6-1, GAL MUT. The significance remains to be explored. The predicted binding is within the range demonstrated to be actual binding in *in vivo* studies [23].

Analyses then were done taking the protein sequences of the 114 genes and performing BLASTp analyses of the *B. vulgaris* proteome (Supplemental data 1). The results show the *G. max* polyubiquitin (Glyma.20G141600), which decreased *H. glycines* parasitism by 31% (p < 0.05) exhibits trypsin cleavage and not have predicted *Hg*Trp1 binding (ipTM = 0.17), nor *Hg*Trp2 (ipTM = 0.15), has a *B. vulgaris* homlog, Bevul.4G043100.1.p, with an e value = 0, and 100% amino acid identity. In contrast, the lowest homology to *B. vulgaris* was Suppressor of NPR1 (Glyma.16G006400) whose overexpression decreased *H. glycines* parasitism by 43% (p < 0.05), is cleaved by trypsin, is not predicted to bind *Hg*Trp1 (ipTM = 0.34) but is predicted to bind *Hg*Trp2 (ipTM = 0.63), with closest *B. vulgaris* homlog, Bevul.6G185100.1.p with an e-value of 8.25E-108 at 31% identity (Table 1; Supplemental data 2). The top matched B. vulgaris proteins are provided (Supplemental data 3).

**Table 1:** The 114 *G. max* defense protein sequences are predicted to be cleaved by trypsin while specific *H. glycines* Trps (CAA74204, and CAA74205) are analyzed for predicted docking.




Footnote: Provided are the soybean accession from the *G. max* genome housed at Phytozome, the gene name, the percent (%) decrease in *H. glycines* parasitism calculated from the female index (FI). Trypsin (Trp) cleavage was determined using PeptideCutter in default settings. The *H. glycines* Trps, CAA74204 and CAA74205 were used in AlphaFold to determine docking. The *G. max* protein sequence was used in BLASTp analyses of the *B. vulgaris* proteome to obtain results with the top match accession, E-value and percent identity provided. The ipTM (interface predicted template modeling), derived from the predicted template modeling (pTM) score, which evaluates the overall accuracy of the predicted structure, measures interface quality between two proteins, showing how well the predicted structure aligns with the actual structure, obtained from the predicted protein-protein binding analysis done in AlphaFold. An ipTM > 0.7 (blue) is considered a strong likelihood of an interaction; ipTM 0.5–0.7 (red) is considered a possible likelihood of an interaction; possible; ipTM < 0.5 (black) is considered a weak/unlikely possible likelihood of an interaction.

## 4 Experimental Design, Materials and Methods

The *T. myopaeformis* genome (https://www.ncbi.nlm.nih.gov/bioproject/PRJNA1026092) was *de novo* sequenced and archived at Genbank [14,6]. The genome sequence was obtained from NCBI [14]. Alkharouf et al. [14] used the pipeline Flye, version 2.9.2, to assemble the PacBio HiFi DNA reads using default values, except for setting the –asm-coverage argument to 50, to reduce memory consumption. The gene finding tool AUGUSTUS 3.5.0 [24] was used for gene prediction analysis in these contigs with the complete gene option enabled and default set for the rest of the parameters. *Drosophila melanogaster* is the most closely related genetic model that was most closely related, phylogenetically, to *T. myopaeformis* so it was used as the reference species to *T. myopaeformis* TmSBRM_v1.0. The identified genes were used to perform a blast against the non-reductant (NR) database to predict genes. For generating the gene functional annotation, the predicted genes were functionally annotated using Blast2GO 6.0 using default values [25]. The gene model was blasted as blastp against the NCBI NR protein database. InterproScan 5.67-99.0 [26] was used under default values for domain finding. Then GO mapping and annotation was performed under default values using GeneOntology 2024-03-28 [27]. BLASTp of the *T. myopaeformis* proteome used the *Bactrocera oleae* Trp XP_014094233 in default settings to identify its Trps. BLASTp queries were done in the *B. vulgaris* RefBeet1.1 and RefBeet3.0 proteomes at https://bvseq.boku.ac.at/ set on default using DV501688 (Bv4_081010_nnrr.t1) *Bv*STI (*Bv*KTI1). The *B. vulgaris* proteome BLASTp was done in Phytozome at https://phytozome.jgi.doe.gov using the *B.vulgaris*_ssp._vulgaris EL10.2_2 proteome. The 9 *T. myopaeformis* Trps (i.e. *Tm*Trp-g7808 [*Tm*Trp1], *Tm*Trp-g3594 [*Tm*Trp2], *Tm*Trp-g23695 [*Tm*Trp3], *Tm*Trp-g23693 [*Tm*Trp4], *Tm*Trp-g3592 [*Tm*Trp5], *Tm*Trp-g7809 [*Tm*Trp6], *Tm*Trp-g23699 [*Tm*Trp7], *Tm*Trp-g23186 [*Tm*Trp8], and *Tm*Trp-g18287 [*Tm*Trp9]). The foundational *H. glycines* Trps used in the deep learning analyses were extracted from NCBI, https://www.ncbi.nlm.nih.gov/ (Accessions: CAA74204, and CAA74205).

Protein–protein interaction predictions were performed using the AlphaFold-Multimer implementation available through the AlphaFold Server. Amino acid sequences of the candidate proteins were provided in FASTA format and submitted as a multimeric complex to enable structural modelling of potential interactions. The server generated predicted complex structures using deep learning–based modelling and multiple sequence alignment information. Model confidence and interaction reliability were evaluated using the predicted Local Distance Difference Test (predicted Local Distance Difference Test (pLDDT)) and the interface predicted TM-score (interface predicted TM-score [ipTM]) provided in the output. Negative control protein-protein predicted binding assay employed the *Aequorea victoria* GFP P42212.1. Trp cleavage prediction was performed by using PeptideCutter [28]. PeptideCutter was set at a lowest cleavage displayed option of 100% [28]. Protein interaction visualization is done on default settings using AlphaFold [29].

## Limitations

The KTI-Trp protein-protein interactions are bioinformatic in nature and are not presenting actual *in vivo* interactions leading to the disarming of the pathogen Trp. *B. vulgaris* gene families from the 114 defense genes does not mean they function in defense to *T. myopaeformis*. However, the predicted binding is within the range demonstrated to be actual binding in *in vivo* studies [23].

## Ethics Statement

The authors have read and follow the ethical requirements for publication in Data in Brief and confirming that the current work does not involve human subjects, animal experiments, or any data collected from social media platforms.

## Credit Author Statement

**SR** Methodology; Software; Validation; Formal analysis; Investigation; Resources; Data Curation; Writing - Original Draft; **YL** Methodology; Software; Validation; Formal analysis; Investigation; Resources; Data Curation; Writing - Original Draft; **NA** Methodology; Software; Validation; Formal analysis; Investigation; Resources; Data Curation; Writing - Original Draft; **CC** Investigation; Resources; **VK** Conceptualization; Methodology; Resources; Visualization; Supervision; Project administration; Funding acquisition; Writing - Original Draft

## Data Availability

NCBITetanops myopaeformis genome (Reference data)

## Appendix Supplementary Materials

Supplemental data 1. The 114 *G. max* defense protein sequences. Image, application 1

Supplemental data 2. BLASTp results of the *B. vulgaris* proteome BLASTp with the 114 *G. max* defense protein sequences. Each tab provides data for a different G. max protein used in the BLASTp queries. The master list tab is provided. Provided are the soybean accession from the *G. max* genome housed at Phytozome, the gene name, the percent (%) decrease in *H. glycines* parasitism calculated from the female index (FI). Trypsin (Trp) cleavage was determined using PeptideCutter in default settings. The *H. glycines* Trps, CAA74204 and CAA74205 were used in AlphaFold to determine docking. The *G. max* protein sequence was used in BLASTp analyses of the *B. vulgaris* proteome to obtain results with the top match accession, E-value and percent identity provided. Image, application 2

Supplemental data 3. The 114 *B. vulgaris* top matches to the 114 *G. max* defense protein sequences (Sequences obtained from Supplemental data 2 BLASTp searches). Image, application 3

## References

[1] Alkharouf N.W., Klink V.P., Matthews B.F. (2007). Identification of Heterodera glycines (soybean cyst nematode [SCN]) cDNA sequences with high identity to those of Caenorhabditis elegans having lethal mutant or RNAi phenotypes. Exp. Parasitol..

[2] Anderson M.A.E., Gonzalez E., Edgington M.P., Ang J.X.D., Purusothaman D.K., Shackleford L., Nevard K., Verkuijl S.A.N., Harvey-Samuel T., Leftwich P.T., Esvelt K., Alphey L. (2024). A multiplexed, confinable CRISPR/Cas9 gene drive can propagate in caged Aedes aegypti populations. Nat Commun..

[3] Bai X., Yu K., Xiong S., Chen J., Yang Y., Ye X., Yao H., Wang F., Fang Q., Song Q., Ye G. (2023). CRISPR/Cas9-mediated mutagenesis of the white gene in an ectoparasitic wasp, Habrobracon hebetor. Pest Manag. Sci..

[4] Beggs J.D. (1978). Transformation of yeast by a replicating hybrid plasmid. Nature.

[5] Chan D.T.C., Baldwin G.S., Bernstein H.C. (2023). Revealing the host-dependent nature of an engineered genetic inverter in concordance with physiology. Biodes. Res.

[6] Fire A., Xu S., Montgomery M.K., Kostas S.A., Driver S.E., Mello C.C. (1998). Potent and specific genetic interference by double-stranded RNA in Caenorhabditis elegans. Nature.

[7] Ijaz M., Khan F., Zaki H.E.M., Khan M.M., Radwan K.S.A., Jiang Y., Qian J., Ahmed T., Shahid M.S., Luo J., Li B. (2023). Recent trends and advancements in CRISPR-based tools for enhancing resistance against plant pathogens. Plants.

[8] Jansen R., Embden J.D., Gaastra W., Schouls L.M. (2002). Identification of genes that are associated with DNA repeats in prokaryotes. Mol. Microbiol..

[9] Jinek M., Chylinski K., Fonfara I., Hauer M., Doudna J.A., Charpentier E. (2012). A programmable dual-RNA-guided DNA endonuclease in adaptive bacterial immunity. Science.

[10] Pourcel C., Salvignol G., Vergnaud G. (2005). CRISPR elements in Yersinia pestis acquire new repeats by preferential uptake of bacteriophage DNA, and provide additional tools for evolutionary studies. Microbiology (Reading).

[11] Fugate K.K., Campbell L.G., Covarrubias-Pazaran G., Rodriguez-Bonilla L., Zalapa J. (2019). Genetic differentiation and diversity of sugarbeet germplasm resistant to the sugarbeet root maggot. Plant Genet. Res.: Charact. Util..

[12] Hein G.L., Boetel M.A., Godfrey L.D., Harveson R.M., Hanson L.E., Hein G.L. (2009). Compendium of Beet Diseases and Pests.

[13] Acharya S., Alkharouf N.W., Chu C., Klink V.P. (2024). The annotation of genomic dataset sequences of the sugar beet root maggot Tetanops myopaeformis, TmSBRM_v1.0. Data Brief.

[14] Alkharouf N.W., Chu C., Klink V.P. (2024). A *de novo* assembly of genomic dataset sequences of the sugar beet root maggot *Tetanops myopaeformis*, TmSBRM_v1.0. Data Brief.

[15] Acharya S., Troell H.A., Billingsley R.L., Lawrence K.S., McKirgan D.S., Alkharouf N.W., Klink V.P. (2024). Glycine max polygalacturonase inhibiting protein 11 (GmPGIP11) functions in the root to suppress Heterodera glycines parasitism. Plant Physiol. Biochem..

[16] Smigocki A.C., Ivic-Haymes S.D., Puthoff D.P., Zuzga S. (2008). Recent advances in functional genomics for sugar beet (Beta vulgaris L.) improvement: progress in determining a role of BvSTI in pest resistance in roots. Sugar Tech..

[17] Urwin P.E., Lilley C.J., McPherson M.J., Atkinson H.J. (1997). Characterization of two cDNAs encoding cysteine proteinases from the soybean cyst nematode Heterodera glycines. Parasitology.

[18] Klink V.P., Darwish O., Alkharouf N.W., Lawaju B.R., Lawrence K.S. (2021). The impact of pRAP vectors on plant genetic transformation and pathogenesis studies including BRI1-associated receptor kinase 1 (BAK1). J. Plant Interact..

[19] Jones J.D.G., Dangl J.L. (2006). The plant immune system. Nature.

[20] Niraula P.M., Sharma K., McNeece B.T., Troell H.A., Darwish O., Alkharouf N.W., Lawrence K.S., Klink V.P. (2020). Mitogen activated protein kinase (MAPK)-regulated genes with predicted signal peptides function in the Glycine max defense response to the root pathogenic nematode Heterodera glycines. PLoS One.

[21] McNeece B.T., Pant S.R., Sharma K., Niruala P., Lawrence G.W., Klink V.P. (2017). A Glycine max homolog of NON-RACE SPECIFIC DISEASE RESISTANCE 1 (NDR1) alters defense gene expression while functioning during a resistance response to different root pathogens in different genetic backgrounds. Plant Physiol. Biochem..

[22] McNeece B.T., Sharma K., Lawrence K.S., Lawrence G.W., Klink V.P. (2019). The mitogen activated protein kinase (MAPK) gene family functions as a cohort during the Glycine max defense response to Heterodera glycines. J.: Plant Physiol. Biochem..

[23] Singh D., Liu Y., Zhu Y.H., Zhang S., Naegele S.M., Wu J.Q. (2025). Septins function in exocytosis via physical interactions with the exocyst complex in fission yeast cytokinesis. Elife.

[24] Hoff K.J., Stanke M. (2019). Predicting Genes in Single Genomes with AUGUSTUS. Curr. Protoc. Bioinformatics.

[25] Conesa A., Götz S., García-Gómez J.M., Terol J., Talón M., Robles M. (2005). Blast2GO: a universal tool for annotation, visualization and analysis in functional genomics research. Bioinformatics.

[26] Zdobnov E.M., Apweiler R. (2001). InterProScan--an integration platform for the signature-recognition methods in InterPro. Bioinformatics.

[27] Ashburner M., Ball C.A., Blake J.A., Botstein D., Butler H., Cherry J.M., Davis A.P., Dolinski K., Dwight S.S., Eppig J.T., Harris M.A., Hill D.P., Issel-Tarver L., Kasarskis A., Lewis S., Matese J.C., Richardson J.E., Ringwald M., Rubin G.M., Sherlock G. (2000). Gene ontology: tool for the unification of biology. The Gene Ontology Consortium. Nat. Genet..

[28] Gasteiger E., Hoogland C., Gattiker A., Duvaud S., Wilkins M.R., Appel R.D., Bairoch A., Walker John M. (2005). The Proteomics Protocols Handbook.

[29] Abramson J., Adler J., Dunger J., Evans R., Green T., Pritzel A., Ronneberger O., Willmore L., Ballard A.J., Bambrick J., Bodenstein S.W., Evans D.A., Hung C.C., O'Neill M., Reiman D., Tunyasuvunakool K., Wu Z., Žemgulytė A., Arvaniti E., Beattie C., Bertolli O., Bridgland A., Cherepanov A., Congreve M., Cowen-Rivers A.I., Cowie A., Figurnov M., Fuchs F.B., Gladman H., Jain R., Khan Y.A., Low C.M.R., Perlin K., Potapenko A., Savy P., Singh S., Stecula A., Thillaisundaram A., Tong C., Yakneen S., Zhong E.D., Zielinski M., Žídek A., Bapst V., Kohli P., Jaderberg M., Hassabis D., Jumper J.M. (2024). Accurate structure prediction of biomolecular interactions with AlphaFold 3. Nature.

