# Supplementary material for: Deep learning analysis of soybean cyst nematode effectors to proven soybean resistance genes and homolog identification in the sugar beet-sugar beet root maggot plant pathosystem
Source: Data Brief. 2026 Apr 11;66:112749. doi: 10.1016/j.dib.2026.112749 (PMC13112395; doi:10.1016/j.dib.2026.112749)
Supplement: Supplementary file 1 — Supplemental data 1. The 114 G. max defense protein sequences. [file mmc1.docx]

**114 resistance gene protein sequences (soybean)**

R-1

>Glyma.12G073100

MGKSYPTVSADYQKAVEKAKKKLRGFIAEKRCAPLMLRLAWHSAGTYDVSSKTGGPFGTIKHPSELAHGANNGLDIAVRLLEPLKAEFPILSYADFYQLAGVVAVEVTGGPEVPFHPGREDKPEPPPEGRLPDATKGSDHLRDVFGKAMGLSDRDIVALSGGHTIGAAHKERSGFEGPWTSNPLIFDNSYFKELLSGEKEGLLQLPSDKALLSDPVFRPLVEKYASDEDAFFADYAEAHQKLSELGFAEA

R-2

>Glyma.08G022300

MEKLIRMFPLLLVLLLCPNFAFAGHDYGQALSKSLLFFEAQRSGYLPHNQRVTWRAHSGLQDGKASGVDLVGGYYDAGDNVKFGLPMAFTVTMMSWSIIEYGKQMAASGELGHAMEAVKWGTDYFIKAHPQANVLYGEVGDGNTDHYCWQRPEDMTTDRHAYKVDPSNPGSDLAGETAAAMAAASIVFRRSNPAYAGELLRHAYQLFDFADKYRGKYDSSITVAQKYYRSISGYNDELLWAAAWLYQASNNQYYLDYLGRNGDSMGGTGWSMTEFGWDVKYAGVQTLVAKFLMQGKSGHHAPVFERYQQKAETFMCSCLGKSNRNVQKTPGGLIFRQRWNNMQFVTSASFLATVYSDYLASSGRNLRCSSGNVPPAELLSLAKSQVDYLLGDNPRATSYMVGYGSNFPQRVHHRGSSIVSIKVNPSFVSCRGGYATWFSSKRSDPNLLTGALVGGPDAYDDFADERDNYEQTEPATYNNAPLIGILARLGGGHGGYNQLLPVVVPAPKPAVTKPQPTPSPKTTPSPASWSGPISIEQKRTTSWVANGKTYYRYSTVVTNKSNKSLNSLNLSISKLYGPIWGVTKSGDSYTFPSWLSSLSAGKSLEFVYIHSASPADVSVANYVLA

R-3

>Glyma.19G197100
MQGCSDAPLSKRLEDKVALITGGASGIGEATARLFLRHGAKVVIADIQDNLGHSLCQNLNSGNNISYVHCDVTNDNDVQIAVKAAVSRHGKLDILFSNAGIGGNSDSSIIALDPADLKRVFEVNVFGAFYAAKHAAEIMIPRKIGSIVFTSSAVSVTHPGSPHPYTASKYAVVGLMKNLCVELGKHGIRVNCISPYAVATPLLTRGMGMEKEMVEELFAEAGNLKGVVLKEEDLAEAALFLASDESKYVSGVNLVVDGGYSVNNTASAEVALGKFSAD

R-4

>Glyma.07G052400
MGSKTKTFTFEEVAKHNHRKDCWIIVKGKVYDVTPFLDDHPGGDEVLVTATEKDATTDFEDIGHSDSATEMMEKYFVGEVDTNTLPAQVTSSSSVRPPTQAPVYNNQSSGFVVKILQYIVPLLILAFAFGLQYYGKKSKSEN

R-5

>Glyma.08G137000
MAPATLSLSLLLLFSLSIAVTVRSSDYDYDCVYTAYVRTGSVLKGGTDSKIGLKLYDKYGYYIYIKNLEAWGGLMGKGYDYFERGNLDIFSGRGPCLDGPVCAVNVTSDGSGSHHGWYLNYVQVTSTGPHLSCAQDQYEVEQWLALDTSPYQLWAVRNHCRYSLDRAQPVSERPGSGSGSAFSILNARA

R-6

>Glyma.15G154700
MGYWKSKVLPKIKKVFEKNSTKKAAAAEATKSFDESKEEYNKAFEEKKTELQTKVVEIYEASSTEIKSLVKEPKEAGLKKNSTEVQKFLEELVKIDFPGSKAASEASSKFGPALASGSVFFVFEKVSTFIVTEEKDVEAPPGVETKTEEETSSVVKEREIVVEEEKKKEKKEEEKPQVIETSDEKKVEEKPAETSAKGEEKPAEEAAAVVEQAEPPKP

R-7

>Glyma.13G158200
MARNLLLVLFAVATLLHGSAAQTRHMVGDATGWIIPAGGAATYTAWASNKTFTVNDTLVFNFATGQHNVAKVTKSAFDACNGGSAVFTLTSGPATVTLNETGEQYYICSVGSHCSAGQKLAINVNRASSTGPSPAPQPRGSGSPPRASPVPTQAPQASSPTPPPRSAPAPAFGPSSEPATFIVGETAGWIVPGNASFYTAWASGKNFRVGDVLVFNYASNTHNVEEVTKANFDACSSASPIATFTTPPARVTLNKSGQHFFICGIPGHCLGGQKLAINVTGSSTATPPSAAAPPTTPSSPSPAGAVTPPPQNSGAASLGVVGVFATLLSVAATFFY

R-8

>Glyma.10G161500
MGSSKLLGIMAMLFIVLLPMAAKGDNITDFFDKVCEEVECGKGSCVVNTSYPLNFVCECDSGWKRTQDDDDEYATSFLPCVIPECSLNYGCQPAPPPVPEKSFPHNFSAFDPCYWAYCGEGTCTKNRTHTHRCECQPNYYNLLNISVFPCYSECTLGSDCSRLGIKVANSSTDSGSQDSSASIFTGRFHWMVMLLMSTGMVMWS

R-9

>Glyma.19G068300
MADQLTDEQISEFKEAFSLFDKDGDGCITTKELGTVMRSLGQNPTEAELQDMINEVDADGNGTIDFPEFLNLMARKMKDTDSEEELKEAFRVFDKDQNGFISAAELRHVMTNLGEKLTDEEVDEMIREADVDGDGQINYEEFVKVMMAK

R-10

>Glyma.02G291000

MEGVILRRVIPSDNSCLFNAVGYVMDHDKKKAAELRQVIAATVASDPQKYCEAFLGKPNAEYCNWILDSEKWGGAIELSILADYYGREIAAYDIQTTRCDLYGQESNYSERVMLIYDGLHYDALVMSPFEEAPEEFDQTIFAVQKNRSIGPVEGLALTFVKDQQRKRSYTDTSNFTLRCGVCQIGVIGEKEAVEHAQATGHVNFQEYR

R-11

>Glyma.08G108900

MAPMPNGRFKSSPTSTSEIMNYEIKMLPTFKNEHIRTHISIPPQQTQQHSLFSLLAFRSSLTLIHSFPPFMDPVSVWGNTPLATVDPEIHDLIEKEKRRQCRGIELIASENFTSFAVIEALGSALTNKYSEGMPGNRYYGGNEYIDQIENLCRSRALQAFHLDAQSWGVNVQPYSGSPANFAAYTAVLNPHDRIMGLDLPSGGHLTHGYYTSGGKKISATSIYFESLPYKVNSTTGYIDYDRLEEKALDFRPKLIICGGSAYPRDWDYKRFREVADKCGALLLCDMAHTSGLVAAQEVNSPFEYCDIVTTTTHKSLRGPRAGMIFYRKGPKPPKKGQPENAVYDFEDKINFAVFPSLQGGPHNHQIGALAVALKQAASPGFKAYAKQVKANAVALGKYLMGKGYSLVTGGTENHLVLWDLRPLGLTGNIYRIGSLPSGFDLLQMSINLTCSLCDCFAGNKVEKLCDLCNITVNKNAVFGDSSALAPGGVRIGAPAMTSRGLVEKDFEQIGEFLHRAVTLTLEIQKEHGKLLKDFNKGLVNNKAIEDLKADVEKFSALFDMPGFLVSEMKYKD

R-12

>Glyma.05G204600

MAALRKAVFFVIAQCFTFSAYAARFEITNRCTYTVWAASVPVGGGVQLNPGQSWSVDVPAGTKGARVWARTGCNFDGSGRGGCQTGDCGGVLDCKAYGAPPNTLAEYGLNGFNNLDFFDISLVDGFNVPMDFSPTSNGCTRGISCTADINGQCPSELKTQGGCNNPCTVFKTDQYCCNSGSCGPTDYSRFFKQRCPDAYSYPKDDPTSTFTCNGGTDYRVVFCP

R-13

>Glyma.14G003400
MAAQALVSSSSLTFSAEATRQSLGPRSLQSPFGFSRKASFLVKAAATPPVKQGSDRPLWFASKQSLSYLDGSLPGDYGFDPLGLSDPEGTGGFIEPKWLAYGEIINGRYAMLGAVGAIAPEILGKAGLIPQETALPWFRTGVFPPAGTYNYWADSYTLFVFEMALMGFAEHRRFQDWAKPGSMGKQYFLGLEKGLGGSGEPAYPGGPFFNPLGFGKDEKSLKDLKLKEVKNGRLAMLAILGYFVQALVTGVGPYQNLLDHLADPVHNNILTSLKFH

R-14

>Glyma.03G171100
MAGKGEGPAIGIDLGTTYSCVGVWQHDRVEIIANDQGNRTTPSYVGFTDTERLIGDAAKNQVAMNPINTVFDAKRLIGRRFSDSSVQSDIKLWPFKVIPGAADKPMIVVNYKGEEKQFAAEEISSMVLIKMREIAEAYLGSTVKNAVVTVPAYFNDSQRQATKDAGVIAGLNVMRIINEPTAAAIAYGLDKKATSVGEKNVLIFDLGGGTFDVSLLTIEEGIFEVKATAGDTHLGGEDFDNRMVNHFVQEFKRKNKKDISGNPRALRRLRTACERAKRTLSSTAQTTIEIDSLYEGIDFYSTVTRARFEELNMDLFRKCMEPVEKCLRDAKMDKRSVDDVVLVGGSTRIPKVQQLLQDFFNGKELCKSINPDEAVAYGAAVQAAILSGEGNEKVQDLLLLDVTPLSLGLETAGGVMTVLIPRNTTIPTKKEQVFSTYSDNQPGVLIQVFEGERARTRDNNLLGKFELSGIPPAPRGVPQITVCFDIDANGILNVSAEDKTTGQKNKITITNDKGRLSKEDIEKMVQEAEKYKSEDEEHKKKVEAKNALENYAYNMRNTVKDDKIGEKLDPADKKKIEDAIEQAIQWLDSNQLAEADEFEDKMKELESICNPIIAKMYQGGAGPDVGGAGAAEDEYAAPPSGGSGAGPKIEEVD

R-15

>Glyma.04G100400
MPEGLTTPHAQKSLFNLSPQLKTHISLDLYIPLFLSPSSTSQHPPIPRFKKRAVIVVTIQKMAGLVVSSQCFLKLLLVVSVFHVSFAARRLNELVQDQSQLLHYHNGPLLYGKIAVNLIWYGNFKPSQKAIITDFVTSLSSPASQSSQPSVATWWKTTEKYYHLSPRKASLSLSLGDQILDETYSLGKSLTGKNLVQLASKGGQRNSINVVLTSADVAVEGFCMSRCGTHGSSASHLKKNSKSYKFAYIWVGNSETQCPGQCAWPFHQPIYGPQSPPLVAPNNDVGLDGMVINLASLLAGTATNPFGNGYFQGPAEAPLEAASACPGVYGKGAYPGYAGDLLVDSTTGASYNVKGANGRKYLVPALYDPSTSSCSTPV

R-16

>Glyma.08G052600

MDPTSIPPPPATTVPFTVEPSNHVTPADNTNTNHPPYDEMIYTAIGALKEKDGSSKRAIGKYMEQVYKDLPPTHSALLTHHLNRLKSAGLLILVKKSYKLPGSDPLPVLQAQKPRGRPPKLKSQPNTELTWPALALNDNPALQSAKRGPGRPKKIAGPVGVSPGPMVPGRRGRPPGTGRSKLPKRPGRPPKPKSVSAISSGLKRRPGRPPKAESNVNVIPFAAPVAPGLPTVQPIVPTASVPNGSPRPRGRPKKIVAGAGAPALSSVGGAPRGRGRPRGVLPLVRPGRPQKLAVGRPKNPARRPVGRPKGSTAAAITAHKAANDDLRRKLEHFQSKVKESLGTLKPYFNHESPVTAIAAIQELEVLSTLDLKAPLRDETHQQPQPQPQVYEQQYPQPQPLLQQFFQPHTSAPS

R-17

>Glyma.13G224900
MSVVADNSANNGSHQVVLNVNGDAPKKCDDSANQDCVPLLQKLVAEVVGTYFLIFAGCASVVVNLDKDKVVTQPGISIVWGLTVMVLVYSVGHISGAHFNPAVTIAHATTKRFPLKQVPAYVIAQVVGATLASGTLRLIFNGKNDHFAGTLPSGSDLQSFVVEFIITFYLMFVISGVATDNRAIGELAGLAVGSTVLLNVMFAGPITGASMNPARSLGPAIVHHEYRGIWIYLVSPTLGAVAGTWAYNFIRYTNKPVREITKSASFLKGSEAE

R-18

>Glyma.20G114200
MGLQIKEPLLFTLVTISLISITKLLHSYFSIPFSPSNLSIAIATLIFVLISYKFSSSSIKHSSTTLPPGPLSVPIFGNWLQVGNDLNHRLLASMSQTYGPVFLLKLGSKNLVVVSDPELATQVLHAQGVEFGSRPRNVVFDIFTGNGQDMVFTVYGDHWRKMRRIMTLPFFTNKVVHNYSNMWEEEMDLVVRDLNVNERVRSEGIVIRRRLQLMLYNIMYRMMFDAKFESQEDPLFIQATRFNSERSRLAQSFEYNYGDFIPLLRPFLRGYLNKCKDLQSRRLAFFNTHYVEKRRQIMAANGEKHKISCAMDHIIDAQMKGEISEENVIYIVENINVAAIETTLWSIEWAVAELVNHPTVQSKIRDEISKVLKGEPVTESNLHELPYLQATVKETLRLHTPIPLLVPHMNLEEAKLGGHTVPKESKVVVNAWWLANNPSWWKNPEEFRPERFLEEECATDAVAGGKVDFRFVPFGVGRRSCPGIILALPILGLVIAKLVKSFQMSAPAGTKIDVSEKGGQFSLHIANHSTVLFHPIKTL

R-19

>Glyma.20G141600

MQIFVKTLTGKTITLEVESSDTIDNVKAKIQDKEGIPPDQQRLIFAGKQLEDGRTLADYNIQKESTLHLVLRLRGGMQIFVKTLTGKTITLEVESSDTIDNVKAKIQDKEGIPPDQQRLIFAGKQLEDGRTLADYNIQKESTLHLVLRLRGGMQIFVKTLTGKTITLEVESSDTIDNVKAKIQDKEGIPPDQQRLIFAGKQLEDGRTLADYNIQKESTLHLVLRLRGGMQIFVKTLTGKTITLEVESSDTIDNVKAKIQDKEGIPPDQQRLIFAGKQLEDGRTLADYNIQKESTLHLVLRLRGGF

R-20

>Glyma.07G189500
MPAVGGINTGGGKEYPGSLTLFVTVTCIVAAMGGLIFGYDIGISGGVTSMDPFLLKFFPSVFRKKNSDKTVNQYCQYDSQTLTMFTSSLYLAALLSSLVAATVTRKFGRKLSMLFGGLLFLVGALINGFAQHVWMLIVGRILLGFGIGFANQSVPLYLSEMAPYKYRGALNIGFQLSITVGILVANVLNYFFAKIKGGWGWRLSLGGAMVPALIITVGSLVLPDTPNSMIERGDREKAKAQLQRIRGIDNVDEEFNDLVAASESSSQVEHPWRNLLQRKYRPHLTMAVLIPFFQQLTGINVIMFYAPVLFSSIGFKDDAALMSAVITGVVNVVATCVSIYGVDKWGRRALFLEGGVQMLICQAVVAAAIGAKFGTDGNPGDLPKWYAIVVVLFICIYVSAFAWSWGPLGWLVPSEIFPLEIRSAAQSINVSVNMLFTFLIAQVFLTMLCHMKFGLFLFFAFFVLIMTFFVYFFLPETKGIPIEEMGQVWQAHPFWSRFVEHDDYGNGVEMGKGAIKEV

R-21

>Glyma.06G266700
MKGQQQLKKSKVVKIDSRKSWEHHITNATNKGYPVMVHFSAYWCMPSIAMNHFFQQLASTYQNVLFLNVDVDEVKEVASKLEIKAIPTFCLMNGGAPVDKIVGANPDELRKRINCFIHQKHSPKSV

R-22

>Glyma.16G006400
MAFQVSHKIKYVAFLCLLLARIDNNSSYLVTSFTFLNDTPQIKYDVFVSFRGADIRQGFLSHLIEAFSRKHIAAFVDHNILKGDELSETLLGAINGSLISLIIFSQNYASSRWCLLELVKIVECRKRDGQIVVPVFYKVDPSDVRHQKGTYGDAFAKHEGKFSLTTIQTWRSALNESANLSGFHSSTFGDEAELVKEIVKCVWMRLNHAHQVNSKGLVGVGKRIVHVESLLQLEAADVRIIGIWGIGGIGKTTIAQEVYNKLCFEYEGCCFLANIREESGRHGIISLKKNLFSTLLGEEYLKIDTPNGLPQYVERRLHRMKVLIILDDVNDSEQLETLARTDWFGPGSRIIVTTRDRQVLANEFANIYEVEPLNFDESLWLFNLNVFKQKHPEIEYYELSKKVVDYAKGIPFVLKLLGHRLHGKEKEIWESQLEGQNVQTKKVHDIIKLSYNDLDQDEKKILMDIACFFYGLRLEVKRIKLLLKDHDYSVASGLERLKDKALISISKENMVSMHDIIKETAWQIAPQESIEDPRSQIRLFDPDDVYQVLKYNKGNEAIRSIVVNLLRMKQLRLNPQVFTKMNKLHFLNFYSVWSSSTFLQDPWGLYLSQGLESLPNELRYLRWTHYPLESLPSKFSAENLVELHLPYSRVKKLWLKVPDLVNLKVLKLHSSAHVKELPDLSTATNLEIIGLRFCVGLTRVHPSVFSLKKLEKLDLGGCTSLTSLRSNIHMQSLRYLSLHGCLELKDFSVISKNLVKLNLELTSIKQLPLSIGSQSMLKMLRLAYTYIETLPTSIKHLTRLRHLDLRYCAGLRTLPELPPSLETLDVRECVSLETVMFPSIPQQRKENKKKVCFWNCLQLDEYSLMAIEMNAQINMVKFAHQHLSTFRDAQGTYVYPGSDVPQWLDHKTRHGYDDDYVTIAPHSSHLGFIFGFIVPEVPYGGSNLKLKITTGAEGEEGNSIIVYLERPHHGIKSNHVYLMYDQACSHFLNSRAKHHPMLKIKVTVASQTLTSQYVPLQIRGFGVSTINSFPQIEELVYSSSKENSDVYPVSVWNG

R-23

>Glyma.14G064300
MASSYRDRTSEFRLLLETLKKIGSPVQPENAPSTSHGESYSRSEFNRKASRIGLGIHETSQKIARLTQLARKSSMFNDPAVEIQELTVLIKNEITALNSALFDLQTVQNTDMADGGYSQDTIVHSTAVCDDLKSKLMGATKHLQDVLAARTENIKAHENRKQIFSKNASRENPLQHQPKPTTEPPPWSNSSNASESLHQELALPSNGAPVGNQLRRRLAVDSTPSQQMEMSMVQQVVPRHDNYAQSRATALHNVESTITELSGIFSHLATMVAHQGELAIRIDDNMDESSANVEGAHSSLLRHLNRISSNRWLLIKIFVILILFLMIFIFFVA

R-24

>Glyma.06G187200
MTQVMRGEVIEKAYAGSWKAHKSPDKPYLIEKINRNDPQEVIFCFPGSGAVRDWYSQKNFGETKIDLGLFPSLRSIGIDEQALVNEAFQKKFQEILSAKPSLADEVEKAMSKKKQIVFAGHSSGGAVAILATLWALENYQPPKSHGGIPPLCVTFGSPLVGNHIFSHATRRENWSHYFFHYVMRYDIVPRILLAPLSSLDPKFEPISQSFNPKSKSFMSDSVGRASAETTSEFYFAIISNAATVTSHAASKLMGTTDTTLETWSNFITLSPYRPFGTYYFCTGNGKSGKKIVITNSNAVLQVLFFSAQLSTEAEAAQVPYRSLRDHTIYGTELQQMGPQNVVHLDQHQLQNLPLSEDGAGGSNATINTALNDLGLIPRARLCLRAAAEWEARRTDNENKIKEKKDFVAKKLDVLREYRKMYKDKRVGFYDGFREHKQGEDDFKANVTRLELAGVWDEMMEKVRSYELPDEFEGNKDYIDLGTELRKLMEPLDIANYYRHGRNYEDSSSSYMIKGRPKRYRYPQRWLEHAERKSHESLSASCFWAEVEELHYKTSRSSNIVSLDQQFKERIEKLEIQIKAWSDRKELDEDVFLEGSTLVKWWKALPQQHKQHSCIKTLIRE

R-25

>Glyma.09G020800
MAYSAEPSSSLSFTSSSHLSNGSVSHNICPSYGSDPGPNLEAISLSKLSSNLEQLLIEPDCDYSDADLVVEGIPVSVHRCILASRSKFFHELFKREKGSSEKEGKLKYNMNDLLPYGKVGYEAFLIFLGYVYTGKLKPSPMEVSTCVDNVCAHDACRPAINFAVELMYASSIFQIPELVSLFQRRLLNFIGKALVEDVIPILTVAFHCQSNQLVNQCIDRVARSDLDQISIDQELPHELSQKVKLLRRKPQQDVENDASVVDALSLKRITRIHKALDSDDVELVKLLLNESDITLDEANALHYAAAYCDPKVVSEVLGLGLANVNLRNSRGYTVLHIAAMRKEPSIIVSLLTKGACASDLTFDGQSAVSICRRLTRPKDYHAKTEQGKETNKDRICIDVLEREMRRNPMAGDACMSSHTMADDLHMKLLYLENRVAFARLFFPSEAKLAMDIAHAETTSEFAGLSASNSKGSNGNLREVDLNETPIVQNKRLLSRMEALTKTVEMGRRYFPHCSEVLDKFMEDDLPDLFYLEKGTHEEQRIKRTRFMELKDDVHKAFNKDKAEFSRSGISSSSSSSSLRDSVVHYKARKV

R-26

>Glyma.14G068700
MGCCLSARIKAESPPRNGLSSKDGNKEEDGLSSKVSTPSDPPTPRTEGEILKSSNMKSFNFSELKTATRNFRPDSVVGEGGFGCVFKGWIDEQTLAPVRPGTGMVIAVKRLNQEGLQGHSEWLTEINYLGQLRHPNLVKLIGYCLEDDQRLLVYEFLTKGSLDNHLFRRASYFQPLSWNFRMKVALDAAKGLAYLHSDEAKVIYRDFKASNILLDSNYNAKLSDFGLAKDGPAGDKSHVSTRVMGTYGYAAPEYMATGHLTKKSDVYSFGVVLLEIMSGKRALDSNRPSGEHNLIEWAKPYLSNKRRIFQVMDARIEGQYTLRESMKVANLAIQCLSVEPRFRPKMDEVVRALEELQDSEDRAGGVGSSRDQTARRSGHSSSSSGPRQHRGRQHETTRK

R-27

>Glyma.17G065100
MASTFSRSLALVSFYIVVCAAGNLNQDFQLTWGDGRAKILNNENLLTLSLDKASGSGFQSKNEYLYGKIDMQLKLVPGNSAGTVTAYYLSSKGSTWDEIDFEFLGNLSGDPYILHTNVFSQGKGNREQQFYLWFDPTADFHTYSILWNPQRIVFSVDGTPIREFKNLESIGVPFPKSQPMRIYSSLWNADDWATRGGLIKTDWSKAPFTASYRNFNANACVWNSGKSSCKSNSSPSSASPTNAWLSQELDSTAQQRLRWVQKNYLIYNYCTDNKRFPQGLPLECKHS

R-28

>Glyma.08G129400
MQSQVVCNGCRSLLLYPRGATNVCCALCNTITSVPPPGMEMSQLYCGGCRTLLMYTRGATSVRCSCCHTVNLVPPASNQVAHVHCGNCRTTLMYPYGAPSVKCALCHFITNVSTNNGRLPIPVHRPNGTNNAGTLPSTSTSMPQSQSQTVVVENPMSVDSSGKLVSNVVVGVTTDKK

R-29

>Glyma.02G195300
MNDLFSGSFSRTNDQVSPDHHHVIEMAATASPTAEGSVNLEKFFQEVEQVKEELKELERLHENLRGSHEKSKILHSAKAVKELRLRMDSDVTLALKNAKLVKVRLEALDRSNQTSQSLPGSGPGSSSDRTRTSVVSGLRKKLKDSMDSFNSLRQKISSEYRETVQRRYYTVTGENPDDKTIDLLISTGESETFLQKAIQQQGRASVMDTIQEIQERHDTVKEIERNLNELHQVFLDMAVLVQSQGEQLDDIESHVARANSYVRGGVQQLHVARKHQKNTRKWTCIAIILLIIIILIIVLPIVLRN

R-30

>Glyma.08G355600
MGQQSLIYSFVARGTVILAEYTEFTGNFTGVAAQCLQKLPSSNNKFTYNCDGHTFNYLVDNGFTYCVVAVESAGRQIPIAFLERIKEDFTKKYAGGKAATAAAQSLNREFGPKLKEQMQYCVDHPEEISKLAKVKAQVSEVKGVMMENIEKVLDRGEKIELLVDKTENLRSQAQDFRQQGTKIRRKMWFQNMKIKLIVLGIIIALILIIVLSVCGGFNCGK

R-31

>Glyma.11G029600
MSMSDSDSSSYAGADYKSFKQISRERLLHEMLRSSKTGDSKSTWKVLIMDKLTVKIMSHSCKMTDITDEGVSLVEDIYKRRQPLPTMDAIYFIQPTRENVIMFLSDMSGRTPLYRKAFVFFSSAISKELVMDIKKDTKVLTRLGALREMNLEYFPIDSQGFITNNERALEELFGDEENNHKGVTCLNVMAKRIATVFASLREFPSVRFRAAKSLDATTMTTFRDLIPTKLAAGVWDCLMKYKKSIPNFPQTETCELLIVDRSIDQIAPVIHEWTYDAMCHDLLNMEGNKYVHEVPGKSGGPAERKEVLLEDHDPIWLELRHAHIAYASEQLHEKMTNFISKNKAAQIQHGSKSSSEMSTRDIQKMVQALPQYSEQIDKLSLHVEIAGKINRIIRESGLRELGQLEQDLVFGDATTKDVIKFFTMTEDIAHENKLRLLMILASVCPEKFEGEKGQNLMRLAKLTEEDMNVVHNMRMLGGQPVTKKKLTTAFGLKFDIHKKKRAARKDRPGEEEKWQLSRFYPIIEELLEKLTKNELSKEDYPCLNDPSPSYHGSPFSGPVNQNPHSMRSRRTPTWARPRGSEDGYSSDSVLRHASSDFRRMGQRIFVFIVGGATRSELRVCHKLTEKLKREIILGSSSLDDPAQFITKLKMITTHELSLDDIQI

R-32

>Glyma.17G076600
MFGSKKSPLKVAKPSSVESWTNPFDSNDEGMDTKKYSSSRKTSSERALTTLGVNTNPFDDGTDANKKSSSTLYGFQSANWNKYKNDFRDSGGLENQSVQELESYAVYKAEETTNSVTNCLKIAENIREEATQTLVTLHQQGEQITRSHHVAADIDHDLTRGEKLLGSLGGLFSKTWKPKKTRAITGPVIVGDDPVRRKGNHLEQREKLGLTSAPKGQSKLRSPPQEPTNAFEKVEVEKNKQDDALSDLSDLLGELKGMAVDMGSEIERHNKALNHLYDDVDELNFRVIGANQRGRRLLGK

R-33

>Glyma.16G010800
MVKLTMIARVTDGLPLAEGLDDGRDLKDAEFYKLQVKALFKNLSRGHYEASRMSVETGPYVFHYIIEGRVCYLTMCDRAYPKKLAFQYLEELRNEFERVNGSQIETAARPYAFIKFDTFMQKTKKLYQDTHTQRNIAKLNDELYEVHQIMTRNVQEVLGVGEQLDQVSQMSSRLSSESRIYADKARDLNRQALIRKWAPVAIVFGVVFVLFWIKNKLW

R-34

>Glyma.11G107300
MGFFGTILGFLGFGVGISIGLVAGYFLFIYFQPTNVEDPEIKPLAEQEQETLQRMFPEIPLWIKNPDFDRLDWLNKFVEYMWPYLDKAICKTAKNIAKPIIAEQIPKYKIDSVEFETLTLGSLPPTFQGMKVYVTDEKELIMEPSVKWAANPNVTVSVKAFGLKATVQVVDLQVFLLPRITLKPLVPSFPCFANIYVSLMEKPHVDFGLKLIGADLMSIPGVYRIVQELIKDQVANMYLWPKTLEVQVLDMSKALKRPVGILHVKVLQAIKLKKKDLLGASDPYVKLKLTEDKLPSKKTTVKHKNLNPEWNEEFNMVVKDPDSQVLEINVYDWEQVGKHDKMGMNVIPLKEVSPEETKRFSLDLLKNMDPNDVQNEKSRGQIVVELTYKPFKEEDLGKGFEETQTVPKAPEGTPAGGGLLVVIVHEAQDVEGKYHTNPHVRLIFRGEEKKTKRIKKNRDPRWEDEFQFMVEEPPTNDRLHVEVVSTSSRNLLHQKEPLGYIDINLGDVVANKRINEKYHLIDSKNGRLQIELQWRTLEA

R-35

>Glyma.07G195900
MASRFGLSSSSSSASSMRVTNTPASDLALTNLAFCSPSDLRNFAVPGHNNLYLAAVADSFVLSLSAHDTIGSGQIALNAVQRRCAKVSSGDSVQVSRFVPPEDFNLALLTLELEFVKKGSKSEQIDAVLLAKQLRKRFMNQVMTVGQKVLFEYHGNNYSFTVSNAAVEGQEKSNSLERGMISDDTYIVFETSRDSGIKIVNQREGATSNIFKQKEFNLQSLGIGGLSAEFADIFRRAFASRVFPPHVTSKLGIKHVKGMLLYGPPGTGKTLMARQIGKILNGKEPKIVNGPEVLSKFVGETEKNVRDLFADAEQDQRTRGDESDLHVIIFDEIDAICKSRGSTRDGTGVHDSIVNQLLTKIDGVESLNNVLLIGMTNRKDMLDEALLRPGRLEVQVEISLPDENGRLQILQIHTNKMKENSFLAADVNLQELAARTKNYSGAELEGVVKSAVSYALNRQLSLEDLTKPVEEENIKVTMDDFLNALHEVTSAFGASTDDLERCRLHGMVECGDRHKHIYQRAMLLVEQVKVSKGSPLVTCLLEGSRGSGKTALSATVGIDSDFPYVKIVSAESMIGLHESTKCAQIIKVFEDAYKSPLSVIILDDIERLLEYVPIGPRFSNLISQTLLVLLKRLPPKGKKLMVIGTTSELDFLESIGFCDTFSVTYHIPTLNTTDAKKVLEQLNVFTDEDIDSAAEALNDMPIRKLYMLIEMAAQGEHGGSAEAIFSGKEKISIAHFYDCLQDVVRL

R-36

>Glyma.18G022500
MADQLSKGEEFEKKAEKKLSGWGLFGSKYEDAADLFDKAANCFKLAKSWDKAGATYLKLASCHLKLESKHEAAQAHVDAAHCYKKTNINESVSCLDRAVNLFCDIGRLSMAARYLKEIAELYEGEQNIEQALVYYEKSADFFQNEEVTTSANQCKQKVAQFAAQLEQYQKSIDIYEEIARQSLNNNLLKYGVKGHLLNAGICQLCKEDVVAITNALERYQELDPTFSGTREYRLLADIAAAIDEEDVAKFTDVVKEFDSMTPLDSWKTTLLLRVKEKLKAKELEEDDLT

R-37

>Glyma.13G051600
MAHSPFLLLPNLQGFWPSLLAMITCFIIMIKALRNNFIENYSNKQKPKLPPGPKPWPIVGNLPEMLANKPAHKWIHNLMKEMNTEIACIRLGNAYVIPVTCPTIAREFLRKQDATFASRSQSVSTDLISNGYSTTIFGPFGAQWKKMKKILTNDLLSPHKHLWLHGQRTEEADNLMFHVYNKCKNVNDGVGGLVNIRSVARHYCGNLTRKIIFNTRYFGKGREDGGPGFEEVEHVDSIFDLLKYVYAFSVSDYMPCLRGLDLDGHEKNVKEALKIIKKYHDPIVQERIKLWNDGLKVDEEDWLDVLVSLKDSNNNPLLTLEEINAQIIELMLATIDNPSNAFEWALAEMINQPELLHRAVEELDSVVGKERLVQESDIPKLNYVKACAREALRLHPIAPFIPPHVSMSDTMVGNYFIPKGSHVMLSRQELGRNPKVWNETYKFKPERHLKSDGSDVDLTEPNLKFISFSTGRRGCPGVMLGTTMTVMLFARLLHGFTWTAPPNVSSINLAESNDDILLAEPLVAVAKPRLASELYQL

R-38

>Glyma.17G039300
MAQLAGADEIESLRNELAEIGRSIRSSFRSHASSFQSVSSINPVQQEVDNNAGEALQWAEIQRLPTFERITSALFDVYDGMETGEKVEGKQVVDVSKLGAQERHMFIEKLIKHIENDNLRLLQKFRNRIDKVGINLPTVELRYQNLCVEAECKIVQGKPIPTLWNTLKEWIFDTTKLPVLKSQNSKISIIKSANGIIKPGRMTLLLGPPASGKTTLLLALAGKLGHSLKVQGEISYNGHMLEEFIPQKSSAYVSQYDLHIPEMTVRETLDFSARCQGVGSRSKLLMEVSRKEKEGGIVPDPDLDAYMKATSINGLKSSLQTDYILKILGLDICADTLVGDPIRRGISGGQKKRLTTGEMIVGPTKALFMDEISNGLDSSTTFQIISCLQHLVHITDATALISLLQPAPETFDLFDDVILMAEGKIVYHGPCDYILEFFEDSGFKCPQRKGTADFLQEVISKKDQAKYWNSTEKPYSYVSIDQFIEKFKDCPFGLKLKEELSKPFDKSQSHKNALVFKKYSLTKWELFNACMMREILLMKKNSFVYVFKSTQLVIVAFVAMTVFIRTRMTVDVLHGNYFMGSLFYSLIILLVDGFPELSMTVSRLAVIYKQKELCFFPAWAYTIPSAVLKIPLSLLESFIWTTLSYYVIGYSPEIGRFFRQFLLLFIIHVTSVSMFRFIASVCQTVVASVTAGTVTILVVLLFGGFIIPKPYMPSWLQWGFWVSPLTYGEIGLTVNEFLAPRWEKMSGNRTLGQQVLESRGLNFDGYFYWISIAALIGFTVLFNVGFTLMLTFLNSPARSRTLISSEKHSELQGQQESYGSVGADKKHVGSMVGSTVQTRKGGLVLPFQPLAVAFHDVQYYVDSPLEMRNRGFTEKRLQLLSDITGSLRPGILTALMGVSGAGKTTLMDVLCGRKTGGIIEGEIRIGGYPKVQETFARVSGYCEQNDIHSPNITVEESVMFSAWLRLPSQIDAKTKAEFVNEVIHTIELDGIKDSLVGMPNISGLSTEQRKRLTIAVELVANPSIIFMDEPTTGLDARAAAVVMRAVKNVVGTGRTVACTIHQPSIDIFEAFDELILMKAGGRLTYAGPLGKHSSRVIEYFESIPGVPKIKDNYNPSTWMLEVTSRSAEAELGIDFAQIYRESTLYEQNKELVEQLSSPPPNSRDLYFPSHFPQNGWEQFKACLWKQHLSYWRSPSYNLMRIIFVAVSSLLFGILFWKQGKKINSQQDVFNVFGAMYSAALFFGINNCSTVLPYVATERTVLYRERFAGMYSPWAYSFAQVLIEVPYIFIQAVVYVIITYPMLSYDWSAYKIFWSFFSMFCNILYYNYLGMLIVSLTPNVQLAAIVASSSYTMLNLFSGYFVPRLRIPKWWIWMYYLCPMSWALNGMLTSQYGDVNKEISAFEEKKTIAKFLEDYYGFHHDFLGVVGVVLIVIPIVIAILFAYCIGNLNFQKR

R-39

>Glyma.11G129600
MAFKGYFLLGLIALVIVRSSKVICEEAANTVSPIIDISLSRKSFPEGFIFGAGSSSYQFEGAAKEGGRGPSVWDTFTHNYPGKIMDRSNGDVAIDSYHNYKKDVGMMKDMNLDSYRFSISWSRILPKGKRSGGINQEGINYYNNLINELVANGIQPLVTLFHWDLPQALEDEYGGFLSPRIVNDFRDYAELCFREFGDRVKYWVTLNEPWSYSQNGYANGRMAPGRCSAWMNLNCTGGDSSTEPYLVTHHQLLAHAATARVYKTKYQASQNGVIGITLVANWFLPLRDTKSDQKATERAIDFMYGWFMDPLTSGDYPKSMRSLVRTRLPKFTVEQSKLLIGSFDFIGLNYYSTTYASDAPQLSNARPSYLTDSLVTPAYERDGKPIGIKIASDWLYVYPRGISDLLLYTKEKYNNPLIYITENGINEYDEPTLSLEESLIDTFRIDYHYRHLFYLQSAIRNGANVKGYYVWSLIDNFEWSSGYTSRFGMIFVDYKNDLKRYQKLSALWFKDFLKKETKLYGSNK

R-40

>Glyma.14G075300
MFHRWSNSHQQDQGSSELYAESKIKELKGVIGPLSGRSLTYCTDACFKRYLEARNWNVDKSKKMLEETLRWRSTYKPEEIRWHEVAMEGETGKLYRASFHDRQGRTVLILRPGMQNTTSMENQLRHLVYLLENAMLNLPPGQEQMSWLIDFTGWSITNNVPLKLARETINILQNHYPERLAIAFLYNPPRVFEAFWKIVKYFLDNKTFQKVKFVYPNNKDSVQVMKSYFDEENLPKELGGKSIMSYNHEEFSRLMVQDDLKCAAFWGSDGKLSNHIVNGNSASVLK

R-41

>Glyma.20G099300
MAAAPARARADYDYLIKLLLIGDSGVGKSCLLLRFSDGSFTTSFITTIGIDFKIRTIELDSKRIKLQIWDTAGQERFRTITTAYYRGAMGILLVYDVTDEASFNNIRNWIRNIEQHASDNVNKILVGNKADMDESKRAVPTSKGQALADEYGIKFFETSAKTNMNVEEVFFSIARDIKQRLADTDSRAEPQTIKINQPDQATSGGQPAQKSACCGS

R-42

>Glyma.18G003500
MSEMASPDPEGLDGVRMTWNVWPRTKVESSKCVIPLAATVALIRPHPDIPRLPYAPLRCKTCSSALNPFSRVDFTAKIWICPFCYQRNHFPPHYHAISETNLPGELYPQYTTVEYILPLSNSLNPSPVFLFLLDTCLIDEEIHFLKSALRRAIGLLPDNALVGFVSFGTQVQVHELGFSDMSKVYVFRGSKEIPAEQILDQLGLSAAGRRPQKGAPGIAGAGGFPNSGITRFLLPASECEYTLNALLDELQTDQWPVPPGRRPARCTGVALSVAAGLLSACNPGTGARIVALVGGPCTEGPGAIVSKDLSDPVRSHKDLDKDAAPFFKKAVKFYEGLAKQLVGQGHVLDIFASALDQVGVAEMKVAVERTGGLVVLSESFGHSVFKDSFKRVFEDGEQSLGLCFNGTLEINCSKEIKIQGIIGPCTSLEKKGPSVADTVIGEGNTTAWKMCGLDKSTCLTVMFDLSSSDRSNTPGAVNPQLYLQFLTSYQDPSGQSVLRVTTVTRRWVDSSVSSEELVQGFDQETAAVVMARFASLKMESEETFDATRWLDRFLIRLCSKFGDYRKDDPSSFTLNPSFSLFPQFMFNLRRSQFVQVFNNSPDETAYFRMLLDRENISNAAVMIQPSLISYSFNALPAPALLDVASIAADKILLLDSYFSVVIFHGMTIAQWRNLGYQNQQEHQAFAQLLRAPHDDAQMIIRERFPVPRLVVCDQHGSQARFLLAKLNPSATYNNAHEMAAGSDVIFTDDVSLQVFFEHLQRLAVQS

R-43

>Glyma.02G098600
MMLHFVQPSKLKTKKMVFEDVYASCDSTTLEHLKELSSKRKAIEDSINECNFVTEAIAKEMSGGLESRFEQDIHKIENYLPFLEILIPYADATSTINQIQISQWNAALKIRWSSALTTSSFFKIKGPKFFQINNLRFELGMTLFLCGGILRERALEVLSTDLVQSATIFRQAAGIYHHLAQEVLPHLEPELPPEKPPEALAAVSSIMSLICLAEAQAVTIRKAEEKGTSSSLLAKLHHGVTLFLEEAIGIFHTVVTQCKDISSRLSEFMYFCKCLHELKGQQYLAESLKACGQIGAAIGVLSSVLNKVKKKIPGEDPWKSIYQKQIQDASEVLRKFVHENDFVWHEKIPSIYELPLPEGTKIVTFIPYSPKRWERQLSFKGLV

R-44

>Glyma.17G063600
MLVANSFDLWRKDGFFSAAEEVQESADIMESAYRAWLREKRERSTPEELNELCRELQTALGTAKWQLEEFDKAVRLSYRHHGDDNTSTRHRQFISAIESQITQVEAALRESYIEEGKQPLRWVNLDEEERDDLAAFLSGTCQTTQSTNDDCMEVKQAKKEDKIVDINTFRNRDISASEKSSKDVISVNKDTDYVIEIKADAVSRSNDDVVSQTDRTTSTRKTWSPPNYGALKIVIADEDEQRDKPTRTVDATPKEKGFRNLFWKQKFEEYPQAMRVVHMFNQRFGRIGICQSQRQFQSPLHLRYGCSVQVTLALMLTIFLIGKLSISCC

R-45

>Glyma.12G194800
MNDLLTDSFVGEASNGQPARQSDVEMGQVPRSNSDMGMEAFNKQIHEADKQIDKLSVLLQKLKEANEESKAVTKASAMKAIKKRMEKDIDEVGKIAHGVKTKIEAISRDNLSNRQKPGCEKGTGIDRARMNMTNALTKKFKDLMTEFQTLRQRIQDEYREVVERRVITVTGTRPDDETIDRLIETGNSEQIFQRAILEAGRGQVVNTVEEIQERHDAVKEIEKKLLDLHQIYLDMAVLVDAQGEILDNIESQVNNAVDHVQRGTSALQNAKKLQKNSRKWMCIAIIILLIIVAIIVVGVLKPWKSS

R-46

>Glyma.19G180200
MSVIDILTRVDSICKKYDKYDVEKQRDSNLSADDAFAKLYASVDADIEALLQKAETADKEKSKASTVAINAEIRRTKARLLEEVPKLQRLAMKKVKGLSSQEFAARNDLALALPDRIQAIPDGAPAAPKQTGSWAASASRPGIKFDSDGKFDDEYFQQTEESSRFRQEYEMRKMKQDQGLDMIAEGLDTLKNMAHDMNEELDRQVPLMDEIDTKVDRASSDLKNTNVRLRDTVNQLRSSRNFCIDIVLLIIILGIAAYLYNVLKK

R-47

>Glyma.14G017700
MASSSDSWVKEYNEALKLADDISGMISEQSSFPASGPETQHHSSAIRRKITILGTRLDSLQSLLSKLPGKQPISEKEMNRRKDMLSNLRSKVNQMASTLNMSNFANRDSLLGPERKPDAMTRMVGLDNNGLVGLQRQIMKEQDDGLEQLEETVASTKHIALAVNEELDLHTRLIDDLDQHVDVTDSRLRRVQKNLAVLNKRTKGGCSCMCMLLSVVGIVALIVVIWLLVKYL

R-48

>Glyma.06G098800
MPPLIDPGLFVFYSLFPFLLFNSISCVNCREGLSTRPVASSDEIQLHIDPGIDFDDEITGLRGQVKKLKNVAEEIGSEVKFQRDFLEQVQMVMIQAQAGVKNNLRRLNKSIVKNGSNNIVHVIAFALVCFFIVYFWSKMSRK

R-49

>Glyma.09G030400
MATPDHLFNLRNNLYLGAYQAAINSGDVTNLSQEDSLERDTLVHRCYIALGQLQFVISEIHDDAPTPLQAVKLLALYFSSPDTKDSAIASLKEWLADPAIANNATLRLVAGLVFLHENDFNEALKHTNAGGTMDLHALNVQIFIKMHRSDYAERQLRIMQQIDEDHTLTQLANAWLNLAVGGSKIQEAYLIFQDLSERYQSTSLLLNGKAVCCMHMGNFDEAETLLVEALNKDARDPETLANLVVCCLHLGKPSNKSFSQLKISHPEHVLVKRVSSAEESFDRALQSFSS

R-50

>Glyma.15G008700
MPEESSFQSRIAKGCTMGFHGVPAEERLHWRSFLVKLGADNLKGVKNEELLVACHKSVYIVYTVLGDVSIYVVGKEEYDELALSEVIFVITSAVKDVCGKPPSERLFLDKYGRICLCLDEIVWKGYLENTEKDRIKRLVRLKPPTEF

R-51

>Glyma.13G088200
MESIISKLRNLDAYPKINEDFYSRTLSGGVITLASSILMLLLFYSELRLYLHAVTETKLVVDTSRAETLRINFDVTFPALPCSILSLDAMDISGEQRLDVKHDIIKKRLDSRGNVIETRQEGIGAPKIEKPLQRHGGRLEHNETYCGSCYGSEVSDDDCCNSCEDVREAYRKKGWALSNPDLIDQCKREGFLQRIKDEEGEGCNVYGFLEVNKVAGNFHFAPGKSFQQSGVHVHDLLAFQKDSFNLSHHINRLTFGEYFPGVVNPLDNVHWTQETPSGMYQYFIKVVPTVYTDVSGHTIQSNQFSVTEHFRTGDMGRLQSLPGVFFFYDLSPIKVTFTEENVSFLHFLTNVCAIVGGIFTVSGILDSFIYHGQRAIKKKMELGKFN

R-52

>Glyma.15G132800
MATKPRLAYLSSTFLLLLVVTVFTSVSAQAPESPSLYNTFLQCLTKYTNNPSNIVFANTNPKFPTILQNYIRNARFNTSSTRKPLLIVTPQQESHVQGTVICAKSVEIQLKIRSGGHDYEGISYISEEPFVILDMFNYRRITVDVKNEVAVVEAGATLGEVYYRIWEKSKVLGFPAGVCPTVGVGGHFSGGGYGNMLRKYGLSVDNVIDAQIVDVKGNLLNRKTMGEDLFWAIRGGGGASFGVILSFTIKLVPVPETVTVFRVEKTLETNVTATDLVVQWQQVAPNTDDRLFMRLLLQPVSSKVVKGTRTVRASVVALFLGGANEVVSILAKEFPLLGLKKENCTEVSWIDSVLWWNDDNSLKNGDKPETLLDRNLNNAGFLKRKSDYVQNAISRDGLEWLFKRMIELGKTGLVFNPYGGKMAEIPSDATPFPHRKGNLYKIQYSVNWDDPSPGAALNFTNQAKRLFSYMTPFVSKNPRSAFLNYRDLDIGVNSFGENSFQEGLVYGTKYFNDNFQRLVKIKTTVDPENFFRNEQSIPVLHGEA

R-53

>Glyma.19G210200
MLEKCLGAQRARRFHRALRHCKVTILCLVLTIVVLRGTIGAGKFGTPEQDLVDIRNRFYTRKLPEPHRLLAELHSTTTPFESSTSTTNYNAFDINTILVDETEDENREKVNPHEAYRLGPKISIWDEQRSRWLRENPNFSNFLRPNKPRVLLVTGSSPKPCENPVGDHYLLKAIKNKIDYCRLHGIEVFYNMALLDAEMAGFWAKLPLIRKLLLSHPEVEFLWWMDSDAMFTDMKFAVPWERYKDSNLVMHGWNEMVYDDKNWIGLNTGSFLLRNCQWSLDILDAWAPMGPKGKVRDEAGKVLTRELKDRPVFEADDQSAMVYLLAKEREKWGGKVYLENGYYLHGYWGILVDRYEEMIENYHPGFGDHRWPLVTHFVGCKPCGKFGDYPVERCLKQMDRAFNFGDNQILHIYGFTHKSLGSRGVKRIRNETSNPLEVKDELGLLHPAFKAVEVS

R-54

>Glyma.19G227800
MPPNITTVVANVTTEQLPKARGGSGRAFVTFLAGNGDYVKGVVGLAKGLRKAKSMYPLVVAVLPDVPEEHREILKSQGCIVREIEPVYPPENQTQFAMAYYVINYSKLRIWEFVEYKKTIYLDGDIQVFGNIDHLFDLPDNYFYAVMDCFCEKTWSHTPQFQIGYCQQCPDKVQWPSHFGSKPPLYFNAGMFVYEPNLDTYRDLLQTVQLTKPTSFAEQDFLNMYFKDKYKPIPNMYNLVLAMLWRHPENVELDKVQVVHYCAAGSKPWRFTGKEENMDREDIKMLVKKWWDIYEDETLDYNNNSVNVERFTSALLDAGGFQFVPAPSAA

R-55

>Glyma.12G214100
MAAEHDSCCSRCVTFLITIGLTALFLWLSLRVDEPKCYLDYIYVPALNKTLNSNSTHNKNTTILFALKLTNGNKDKGIQYDDVLLSFRVFESVNLTRPLGNATVQRFYQGHQKKATKHGNFSGGGGNLTAAVAGRMWYRVDYATAVKYKILFWYTKRHRLWGGANVEIGDSGMKVYRKAVRLGGKNPVVIESGASKLSGRYRALLLSLLLPFCGLWV

R-56

>Glyma.12G073700
MENNAESENPKGKGKGIPIHGGKYVRYNILGNHFQVYSKYAPPLQPVGRGAYGIVCCATNSETKEGVAIKKIGNAFDNRIDAKRTLREIKLLCHMEHDNIIKIKDIIRPAERENFNDVYIVYELMDTDLHQIIQSNQALTDEHCQYFLYQLLRGLKYIHSANVLHRDLKPSNLLLNANCDLKICDFGLARTTSETDFMTEYVVTRWYRAPELLLNCSEYTSAIDIWSVGCILMEIIRREPLFPGKDYVQQLALITELIGSPNDSDLGFLRSDNAKKYVKQLPHVEKQSFAERFPDVSPLAIDLAEKMLVFDPSKRITVEEALNHPYMASLHEINEEPTCPTPFIFDFEQTILNEEDIKELIWKESLNFSQDHQMLE

R-57

>Glyma.08G017400
MTTKGSSSSAASSASGDAKIKRVLTHGGKYAQYNVYGNLFEVSSKYVPPIRPIGRGVNGIVCAAVNSETHEQVAIKKIGNAFDNIIDAKRTLREIKLLRHMDHDNIIAIKDIIRPPKKETFNDVYIVYELMDTDLHHIIHSDQPLSEEHCQKLVQYFLYQLLRGLKYVHSANVLHRDLKPSNLLMNANCDLKIGDFGLARTTSETDFMTEYVVTRWYRAPELLLNCSEYTSAIDVWSVGCILGEIMTREPLFPGKDYVHQLRLITELLGSPDDASLEFLRSDNARRYIRQLPQYRKQKFSTRFPNMLPKALDLLEKMLIFDPNKRITVDEALCHPYLSSLHNINDEPVCPRPFSFDFDQPTCTEEHMKELIWKESVKFNPDPPC

R-58

>Glyma.14G028100
MQQDQRKKGSMEMEFFSDYGDVSRYKIQEVIGKGSYGVVCSAIDTHTGEKVAIKKIHDIFEHVSDAARILREIKLLRLLRHPDIVEIKHVMLPPSRRDFKDIYVVFELMESDLHQVIKANDDLTKEHYQFFLYQLLRALKYIHTANVYHRDLKPKNILANANCKLKICDFGLARVAFNDTPTTVFWTDYVATRWYRAPELCGSFYSRYTPAIDIWSIGCIFAEVLIGKPLFPGKNVVHQLDLMTDLLGTPSLDTISKVRNDKARRYLTSMRKKQPIPFAQKFPNADPLALRLLERLLAFDPKDRPTAEEALADPYFKGLSKIEREPSCQPITKMEFEFERRRVTKEEIGELIFREILEYHPQLLKDYINGTERTNFLYPSAVDQFKQQFSHLEENGGKSYPIMPLERKHASLPRSTMVHSNMVPSKEQSNIASCINRQTTGEFNNNSRDTESPAPRSIPGLQKFSQAKPGKVVGPVIPYEYASVVKGTYDPRTFMRGSVLPSQPILPTNHYQRSTSGKQERSATEADKGVSLQSKLAQQCGVNAKIAPDTAINIDTNPFFMTRAGVNKIEQDDRIAIETKLLQSKAHQYGGISTTAHRKVGPVQYGMTRLF

R-59

>Glyma.07G255400
MPHDQRKKSSVDIDFFTEYGEGSRYKIEEVIGKGSYGVVCSAYDTHTGEKVAIKKINDIFEHVSDATRILREIKLLRLLHHPDIVEIKHILLPPSRREFKDIYVVFELMESDLHQVIKANDDLTPEHYQFFLYQLLRGLKYIHTANVFHRDLKPKNILANADCKLKICDFGLARVAFNDTPTAIFWTDYVATRWYRAPELCGSFFSKYTPAIDIWSIGCIFAELLTGKPLFPGKNVVHQLDLMTDFLGTPSPEAIARVRNEKARRYLCCMRKKKPVPFSQKFPNVDPLALRVLERMLAFEPKDRPTAEEALAYPYFKGLAKVEREPSAQPVTKMEFEFERRRITKEDVRELIYREILEYHPKMLKEHLEGEEPTGFMYPSAVDHFKKQFAYLEEHYGKAGTVTPPERQHASLPRPCVLYSDNSRQNMAEVADDISKCIIKEVEKPAMDRTGGIPMTRLPLQAPQNIQGVMTDRIASGVAARPGKVVGSILHYNNCGAAVTADAEQRRMGRNPSVSAQYAAPSSSYPRRNPSCKSERAEEVIEGANGLQTKPQHITQKVAAAALGGPGGNWY

R-60

>Glyma.U021800
MAGVNPNGVADFPATPTHGGQFIQYNIFGNLFEVTAKYRPPIMPVGRGAYGIVCSLLNTETNELVAVKKIANAFDNHMDAKRTLREIKLLRHLDHENVIGLRDVIPPPLRREFNDVYIATELMDTDLHHIIRSNQNLSEEHSQYFLYQILRGLKYIHSANVIHRDLKPSNLLLNSNCDLKIIDFGLARPTLESDFMTEYVVTRWYRAPELLLNSSDYTSAIDVWSVGCIFMELMNKKPLFPGKDHVHQMRLLTELLGTPTEADLGLVKNEDARRYIRQLPQYPRQPLAQVFPHVHPAAIDLVDKMLTVDPTKRITVEEALAHPYLEKLHDVADEPICMEPFSFDFEQQQLDEEQIKEMIYREALALNPEYA

R-61

>Glyma.06G029700
MATPVEPPNGIRTEGKHYYSMWQTLFEIDSKYVPIKPIGRGAYGIVCSSVNREINEKVAIKKIQNAFENRVDALRTLRELKLLRHLHHENVIALKDIMMPVHRNSFKDVYLVYELMDTDLHQIIKSSQALSNDHCQYFLFQLLRGLKYLHSANILHRDLKPGNLLINANCDLKICDFGLARTNCSKNQFMTEYVVTRWYRAPELLLCCDNYGTSIDVWSVGCIFAELLGRKPIFPGSECLNQLKLIINILGSQREEDIEFIDNPKAKKYIKSLPYSPGTPLSQLYPNAHPLAIDLLAKMLVFDPTKRISVTQALQHPYMAPLYDPNCDPPAVIPIDLDIDEDLGEEMIRDMMWKEMLHYHPESAMESAGLC

R-62

>Glyma.02G138800
MEGGGAAPPADTVMSDAAPPPQQAMAMGIENIPATLSHGGRFIQYNIFGNIFEVTAKYKPPIMPIGKGAYGIVCSALNSETNEHVAIKKIANAFDNKIDAKRTLREIKLLRHMDHENVVAIRDIVPPPQREIFNDVYIAYELMDTDLHQIIRSNQGLSEEHCQYFLYQILRGLKYIHSANVLHRDLKPSNLLLNANCDLKICDFGLARVTSETDFMTEYVVTRWYRAPELLLNSSDYTAAIDVWSVGCIFMELMDRKPLFPGRDHVHQLRLLMELIGTPSEADLGFLNENAKRYIRQLPLYRRQSFQEKFPHVHPEAIDLVEKMLTFDPRKRITVEDALAHPYLTSLHDISDEPVCMTPFNFDFEQHALTEEQMKELIYREALAFNPEYQQ

R-63

>Glyma.07G066800
MSVVESGEHNNIRGVPTHGGRYVQYNIYGNLFEVSRKYVPPIRPVGRGAYGIVCAAVNAETGEEVAIKKIGNAFDNRIDAKRTLREIKLLRHMDHANIMSIKDIIRPPQKENFNDVYLVSELMDTDLHQIIRSNQQLTDDHCRYFLYQLLRGLKYVHSANVLHRDLKPSNLLLNANCDLKIADFGLARTTSETDFMTEYVVTRWYRAPELLLNCSEYTAAIDIWSVGCILGEIITRQPLFPGKDYVHQLRLITELIGSPNDASLGFLRSDNARRYVKQLPQYPKQNFSARFPDMSPGAVDLLEKMLIFDPNRRITVDEALSHPYMAPLHDINEEPVCTRPFSFDFEQPSFTEEDIKELIWRESVKFNPVPPVY

R-64

>Glyma.12G073000
MAGVNPNGVADFAAVPTHGGQFIQYNIFGNLFEVTTKYRPPIMPIGRGAYGIVCSLLNTETNELVAVKKIANAFDNHMDAKRTLREIKLLRHLDHENVIGLRDVIPPPLRREFNDVYIATELMDTDLHHIIRSNQNLSEEHCQYFLYQILRGLKYIHSANVIHRDLKPSNLLLNSNCDLKIIDFGLARPTLESDFMTEYVVTRWYRAPELLLNSSDYTSAIDVWSVGCIFMELMNKKPLFPGKDHVHQMRLLTELLGTPTEADLGLVKNEDARRYIRQLPQYPRQPLAQVFPHVHPAAIDLVDKMLTVDPTKRITVEEALAHPYLEKLHDVADEPICMEPFSFDFEQQQLDEEQIKEMIYREALALNPEYA

R-65

>Glyma.15G062400
MGLCKVSFPVLCVLGLVMIVSHVANAQDSPADYVNAHNAARSEVGVQNLAWDDTVAAFAQNYANQRKGDCQLIHSGGGGQYGENLAMSTGDLSGTDAVKLWVDEKSNYDYNSNSCVGGECLHYTQVVWRDSVRLGCAKVACDNGGTFITCNYAPPGNYVGQRPY

R-66

>Glyma.08G308700

MASTSGTKGGPFEMGRQPSKRMVRAPTRNVELGNDEGVVDSEIVPSSLAVLVPILRAALEIEEENPRVAYLCRFHAFEKAHTMDPTSSGRGVRQFKTYLLHKLEKEGELTEKSVQRSDARELQTYYQHFYEKKIRDGEFSQRPEEMAKNVQIATVLYEVLKTMVAPQNTEDKTRRYAEDVEHKRGQYEHYNILPLYAVGVKPAIMELPEIKAAIAALCRVDNLPMPIIRARPDASQDDSTMPTDRLKKVNDILDWIASVFGFQKGNVANQREHLILLLANINIRDRPEPSYQLHVETIEKLVGKIFKNYESWCHYVRCESNLRYLEDYDLQQIELIYIALYLLIWGEASNIRFMPECLCYIFHHMCHEVYNILDKNLARVTGSTDLVEGRDDEHFLREVITPIYQVLMKEAKRNNKGKASHSNWRNYDDLNEYFWSKKCFDDLSWPLNSKADFFRHSDETQTRRRGRSHANTAVGKRKPKTNFVEVRTFLHLYRSFDRMWIFFILALQAMIIIAWSSLGPVGFFFDGDVFRNVMTIFITYAFLNFLQVTLDIILTWNALKNMKFTQLLRYFLKFVVAAVWVVVLPVCYSSSQVNPSGLIRFVTSWAGDWGNQSLYTYVVVLYMLPNIVAAILFFLPPLRRKLERSNMRILTFLMWWAQPKLYVGRGMHENMFSLLKYTLFWIMLLISKLAFSYYVEISPLVGPTKLIMGMSIDNYQWHEFFPENETHNICIVIAIWAPIMLVYFMDAQIWYAIYATLFGGIIGAFSHLGEIRTLGMLRSRFQSVPIAFSQRFWTGRDRKTKQEESDETYERQNIAYFSQVWNEFINSMREEDLISDRDRDLLLVPYSSSYVSVIQWPPFLLASKIPIAVDMAKDYKKETDDDLVRKIKSDGYMYSAVVECYETLRDIILNLLLDEDDRRVVMRICGRVEECIHEEKFVKEFNMSGLPSLSEKLEKFLTLLRSEDGKLESQIVNVLQDIVEIIIQDVMFDGHLLLQTPQQTPHEYHVERGQKFVNIDTSFTHNTSVMEKVIRLHLLLTVKESAINVPQNIEARRRITFFANSLFMNMPKAPKVRDMLSFSVLTPYFKEDVLYSDEELNKENEDGISILFYLKKIYPDEWANFNERVKSDYLEEDKELIRQWASYRGQTLYRTVRGMMYYWQALILQYFIESAGDNALSEGYRTMDSYEKNKKLLEEAQAMADLKFTYVVSCQVYGSQKKSKNTRDRSCYTNILSLMLTHSALRVAYIDETEDTKDGKSQKVYYSVLVKGGDKYDEEIYRIKLPGPPTEIGEGKPENQNHAIVFTRGEALQTIDMNQDNYYEEAFKMRNVLEEFRRGRSGQRTPSILGIREHIFTGSVSSLAWFMSNQETSFVTIGQRILANPLRVRFHYGHPDIFDRLFHITRGGISKASKVINLSEDIFAGFNSTLRQGYITHHEYIQVGKGRDVGMNQISLFEAKVANGNGEQTLSRDVYRLGRRFDFYRMLSFYFTTVGFYFSSMITVLTVYVFLYGRLYMVLSGVEREILQSPNMHQSKALEEALATQSVVQLGLLLVLPMVMEIGLEKGFRTALGDFIIMQLQLASVFFTFQLGTKAHYYGRTLLHGGSKYRPTGRGFVVFHAKFADNYRMYSRSHFVKGLEILILLIVYEVYGSSYRSSHLYLFITISMWFLATSWLFAPFLFNPSGFDWQKTVDDWTDWKRWMGNRGGIGISSDKSWESWWDEENEHLKYSNLRGKIIEIILAFRFFMYQYGIVYHMDITHHNKDLLVFGLSWAVLVIILIVLKMVSMGRRRFGTDFQLMFRILKALLFLGFLSVMTVLFVVCGLTIADLFAAIIAFMPSGWAIILIAQACKVCLKGAKLWDSVKELSRAYEYVMGLIIFLPTAILSWFPFVSEFQTRLLFNQAFSRGLQISMILAGKKDTYKSD

R-67

>Glyma.10G295100
MSRAEEHWERLVRAALRRERTGDDAYGRPVGGIAGNVPSALAKNRDIDEILRVADEIEDDDPNISRILCEHAYSLSQNLDPNSEGRGVLQFKTGLMSVIKQKLTKREAGTIDRSQDIARLQEFYKSYREKHNVDKLCEEEMKLRESGAFSRDLGELERKTLKRKRVFATLKVLGTVLEQLCEEEIPDELKRLMDSDSALTEDLIAYNIIPLDASSSTNAIVYFPEVQAAVSALKYFNGLPELPRGYFIQPTRNATMFDFLQCTFGFQKDNVANQHEHIVHLLANEQSRLRIPEDAEPKLDEAAVQAIFLKSLQNYINWCDYLGIQPVWSSLEAVSKEKKLLYVSLYFLIWGEASNIRFLPECLCYIFHHMAREMDEILRQQIAQPANSCIYDSKDGVSFLDNVIFPLYDIVSAEAANNDNGKAPHSSWRNYDDFNEYFWSIHCFELSWPWRKSSPFFQKPQPRSKKMLIPGSSRHQGKTSFVEHRTFFHLYHSFHRLWIFLFMMFQGLTILAFNNGKLNAKTLREVLSLGPTFVVMKFFESVLDIFMMYGAYSTTRRSAVSRIFLRFLWFSLASVFITFLYVKALQEESNINGNSVVFRLYVIVIGIYAGVQFFISFLMRIPACHRLTNQCDRFPLISFVKWLRQERHYVGRGMYERSSDFIKYMLFWLVILSAKFAFAYFLQIRPLVDPTRAIIKEDNINYSWHDFVSKNNHNALTVVSVWAPVVAIYLLDIYVFYTLVSAVYGFLLGARDRLGEIRSLEALHRLFEQFPRAFMDTLHVPLPNRSSHQSSVQVVEKNKVDAARFAPFWNEIIRNLREEDYVTNFEMELLLMPKNSGDLPLVQWPLFLLASKIFLARDIAVESKDTQDEPWDRISRDDYMMYAVQECYYAIKFILTEILDDVGRKWVERIYDDINASITKRSIHVDFQLNKLALVITRVTALMGILKETETPELEKGAVRAVQDLYDVMRHDVLSINMRENYDTWSLLKKARDEGHLFEKLKWPKNTDLKMQVKRLYSLLTIKESASSIPKNLEARRRLQFFTNSLFMKMPCAKPVREMLSFSVFTPYYSEIVLYSMAELLKKNEDGISILFYLQKIYPDEWKNFLARIGRDENTLESELYDNPSDILELRFWASYRGQTLARTVRGMMYYRKALMLQTYLERTTAGDLEAAIGCEEVTDTHGFELSPEARAQADLKFTYVLTCQIYGKQKEEQKPEAADIALLMQRNEALRVAFIDVVETLKEGKVNTEYYSKLVKADINGKDKEIYSVKLPGNPKLGEGKPENQNHAIVFTRGNAVQTIDMNQDNYFEEALKMRNLLEEFHSDHGLRPPSILGVREHVFTGSVSSLASFMSNQETSFVTLGQRVLANPLKVRMHYGHPDVFDRIFHVTRGGISKASRVINISEDIYSGFNSTLRQGNITHHEYIQVGKGRDVGLNQIALFEGKVSGGNGEQVLSRDVYRLGQLFDFFRMLSFYFTTVGYYFCTMLTVLTVYAFLYGKAYLALSGVGETIEERARITKNTALSAALNTQFLFQIGIFTAVPMILGFILEQGFLRAIVSFVTMQFQLCTVFFTFSLGTRTHYFGRTILHGGARYQATGRGFVVRHIKFSENYRLYSRSHFVKGLEVALLLIVYLAYGSNEGGALSYILLSISSWFMALSWLFAPYLFNPSGFEWQKVVEDFRDWTNWLLYRGGIGVKGEESWEAWWEEELAHIRSLGSRIAETILSLRFFIFQYGIVYKLNVKGTSTSLTVYGLSWVVLAVLIILFKVFTFSQKISVNFQLLLRFIQGVSLLVALAGLVVAVILTKLSLPDIFASMLAFIPTGWGILSIAAAWKPVMKRLGLWKSVRSIARLYDAGMGMLIFVPIAFFSWFPFVSTFQTRLMFNQAFSRGLEISLILAGNNPNTGI

R-68

>Glyma.13G239300
MNLRQRPVAARGGATNLPRPPPLNSVFNIIPVHDLFTDHPSLRYPEVRAAAAALRTVGDLPKHQFMRWEPEMDLLDWLRLLFGFQLDNARNQREHLVLHLANSQMRLEPPPVIVDALDAGVLRRFRRKLLHNYSAWCSFLGLKSNVLLSRRRDPTDLRRELLYVSLYLLVWGEAGNLRFTPECLCYIYHFMAKELNHVIDEHGDPDTGRPYMPTVSGELGFLKSVIMPIYNTIKVEVDSSRNGKAPHSAWRNYDDINEYFWSRRCLKRLGWPLNFECNFFGTTPKEKRVGKTGFVEQRSFWNVYKSFDRLWVMLILFFQAAIIVAWEGTTYPWEALEKRDVQVKMLTVFITWSALRFLQSVLDAGTQYSLVTRETRWLGVRMALKSMAAIMWTVLFSVFYGMIWIEKGSRPIWSDAANQRIYTFLKVVLFFLIPELLALVLFVVPWLRNVIEESDWKIVYLLTWWFHTRIFVGRGVRQALIDNVKYTVFWVAVLASKFSFSYLFQIEPLVAPTKALLNLKNIRYKWHEFFNNTNRVAVVLLWVPVVLVYLMDLQIWYSIFSAFYGAAIGLFSHLGEIRNVTQLRLRFQFFASAMQFNLMPEEKLLSQQATLLKKLRDAIHRLKLRYGLGQPFNKIESSQVDATRFALIWNEIMITFREEDIISDRELELLKLPPNCWNIRVIRWPCSLLCNELLLAVSQAKELENESDWSLWLKICKNEYRRCAVIEAYDSVKYLFPKVLKAEKEEYSIMTNIFGADKNPRADEILYLMQNNEALRVAYVDEVSLGREGTEYYSVLVKYDQQLQSEVEIYRIRLPGPLKLGEGKPENQNHAIIFTRGDAVQTIDMNQDNYFEEALKMRNLLEEFNAYYGIKKPTILGVRENIFTGSVSSLAWFMSAQDTSFVTLGQRVLANPLKVRMHYGHPDVFDRFWFLGRGGVSKASRVINISEDIFAGFNCTLRGGNVTHHEYIQVGKGRDVGLNQISMFEAKVASGNGEQVLSRDVYRLGHRLDFFRMLSVFYTTIGFYFNSMVIVLMVYAFLWGRLYMALSGIEHAALKNATNNKALGAVLNQQFAIQVGIFTALPMIFENSLEHGFLPALWDFLTMQLQLASLFYTFSLGTRTHFFGRTILHGGAKYRATGRGFVVAHKSFAENYRLYARSHFAKGIELGIILIVYAAHSPLARDTFVYIAMTISSWFLVVSWIMSPFVFNPSGFDWLKTVYDFEDFINWIWYPGGPFKKAEHSWETWWYEEQDHLKTTGIWGKLLEIILNLRFFFFQYGIVYQLGIAGGNNSIAVYLLSWIVMVVIVAIYIIMAYARDKFATKEHLYYRLVQLLVIVITVLVLFLLLEFAHLKFIDLLSSFLAFVPTGWGMISIALVLRPFLQTTKVWETVVSLARLYDLLFGVIVMAPMAIVSWLPGFQSMQTRILFNEAFSRGLQISRIVSGKKSV

R-69

>Glyma.15G268800
MSYRRGSDQPPQRRILRTQTAGNLGADPILDSEVVPSSLVEIAPILRVANEVEASNKRVGYLCRFYAFELAHRLDPQSSGRGVRQFKTALLQRLEKENVTTQEGRKKSDAREMQAFYRQYYEKYIQALDKAADKDRAQLTKAYQTAAVLFEVLKAVNRIEDIPVSDEIMEAHIKVEEQKQLYAPYNILPLDPNSGKEAIMRYHEIQASVSALRNTRGLPWPKEHGNKVNEDILDWLQLMFGFQKDNVENQREHLILLLANVHIRQVPKPDQQPKLDDRALNEVMKKLFRNYKKWCKYLGRKSSLWLPTIQQEMQQRKLLYMGLYLLIWGEAANLRFMPECLCYIYHHMAFELYGMLAGNVSPLTGEPVKPAYGGDNEAFLMKVVKPIYDVIAKEAKRSNMGKAKHSHWRNYDDLNEYFWSVDCFRLGWPMRVDSDFFSVPFPQQERQVNKDEENRGPASDRWSGKTNFVEIRTFWHIFRSFDRMWSFYILCLQAMIIIAWNGSGELSSIFRGDVFKQVLSIFITAAILKLAQAILDIFLSWKARKVMSLHVQLRYIFKAILAAAWVIILPVTYAYSWKNPSGFAQTIKNWFGNGTGSPSLFILAVFIYLSPNILSALLFVFPFIRQFLERSNNGVVKLMMWWSQPRLFVGRGMQEGPISLLKYTSFWVMLILSKLAFSYYLEIKPLVAPTKAIMNAHVSVYRWHEFFPHARNNIGVVIAIWSPIILVYFMDTQIWYAIFSTIVGGIYGAFRRLGEIRTLELLRSRFESIPGAFNACLIPTEQTEKKKKRGLKATFSRRFDQVASNKEKESARFAQLWNKIITSLREEDLIDNREMDLMLVPYSADRSLNLIQWPPFLLASKIPIAVSMAQDSLGKGQELEKRLLRDKYMKSAVEECYASFKSIINFLVLGERETMVIQNIFQRVDEHIENKAVLNELNLSAVPSLYERFVKLIERLLENKEEDKDSIVIFLLDMLEIVTRDIMDGDIEGLLDSSHGGSYGKDERFTPLEKQYKFFGKLQFPVKTDIDAWAEKIKRLQLLLTVKESAMDVPSNLDARRRISFFSNSLFMDMPPAPKVRNMLSFSVLTPYFDEAVLFSLNNLEKQNEDGVSILFYLQKIFPDEWKNFVQRFDNKSEEKLRVENEEDLRLWASYRGQTLTKTVRGMMYIRQALELQAFLDMAKDEELMKGYKAAELESMESTTGERSLWTQCQSLADMKFTYVVSCQQYSIHKRSGDSRAKEILKLMIKYPSLRVAYIDEVEEHIKDSSRKTDKVYYSALVKAALPSKSNDSSETVQSLDQVIYKIKLPGPAILGEGKPENQNHAIIFTRGEGLQTIDMNQDNYMEEAFKMRNLLQEFLKQHDGPRMPTILGLREHIFTGSVSSLAWFMSNQEHSFVTIGQRLLAYPLKVRFHYGHPDVFDRLFHLTRGGVSKASKVINLSEDIFAGYNSTLREGNVTHHEYIQVGKGRDVGLNQISMFEAKIAAGNGEQTMSRDIYRLGHRFDFFRMLSCYYTTIGFYFSTLITVLTVYVFLYGRLYLALSGVEESLNKQRAIRDNKALQVALASQSVVQIGFLLALPMLMEIGLERGFREALSEFVLMQLQLAPVFFTFSLGTKTHYYGRTLLHGGAEYKGTGRGFVVFHAKFADNYRLYSRSHFVKGIELMILLVVYHIFGHGYRGVVAYILITVTMWFMVGTWLFAPFLFNPSGFEWQKIVDDYTDWQKWISNRGGIGVSPQKSWESWWEKEHEHLRHSGKRGIATEIILALRFFIYQYGLVYHLSVTDEKTQSVLVYGLSWLIIFVILGLMKGVSVGRRRLSADYQLLFRLIEGSIFLTFLAIFIILILLANMTIKDIIVCILAVMPTGWGMLLIAQACKPLIEKTGFWGSVRALARGYEVIMGLLLFTPVAFLAWFPFVSEFQTRMLFNQAFSRGLQISRILGGQRSERSSNHKE

R-70

>Glyma.19G020700
MTKIFVLLCLLFLASSGFVHGSSKKDHIRLFELKKGDFSIKVTNWGATLVSVILPDKNGKLGDIVLGYDSPKAYTNDTSYFGATVGRVANRIGGAQFTLNGIHYKLVANEGNNTLHSGPKAFSDVLWKVTRYIKDDDKPRITFSYHSYDGEGGFPGDLLVTVSYILGKNSLSIIMKAKALNKPTPVNMVNHAYWNLGNHNSGNILDEVVQIFGSQVTLFDDNLIPTGQYASVRGTPNDFLEPHIVGERINQLPKTNGYNINYVLDGEKGNAEIKLAAIAVDKKSGRVMKLFTNAPGLQFYTANFVKNDKGKGGFVYQPRSALCLESQAFPDSVNHPNFPSTIVTPDKPYKHVMLLKFSTKVPYAFSQF

R-71

>Glyma.13G178700
MAKALASVLLFLLVAVSNVFAVELELETLPPSYPHPHPVHSPTPAPLHPPANAPHPHHHHHHHHPPAPAPAPAPVPSPSHHNYPPTPAPAKPPTHHHHHHPPAPVNPPPVPVHPPVKPPVPVHPPVKPPVPVHPPVKPPVPAHPPVKPPVVPVHPPVKPPVVPVHPFPRSFVAVQGVVYVKSCKYAGVDTLLGATPLLGAVVKLQCNNTKYKLVQTSKSDKNGYFYIEAPKSITTYGAHKCNVVLVSAPYGLKASNLHGGVTGALLRPEKPFLSKRLPFVLYTVGPLAFEPNCH

R-72

>Glyma.09G096700
MESCAQFGFWVVFILFLAFQIPPNYGFYLPGSYPHNYGVSDELWVKVNSLTSIDTEIPFSYYSLPFCKPEGGIKDSAENLGELLMGDRIENSPYRFRMYSNESEIYLCRIEALSGDQFKILKERIDEMYQVNLILDNLPAIRFTQKEGYFMRWTGYPVGIKIEDAYYVFNHLKFNVLVHKYEETNVARVMGTGEGAELIPVVKQGSSEKPGYMVVGFEVIPCSIMHNADSAKTLKMYEKYPSSIRCDPATVAMPIKEGQPVVFTYEVTFEESDIKWPSRWDAYLKMEGAKVHWFSILNSLMVITFLAGIVLVIFLRTVRRDLTRYEELDKEAQAQMNEELSGWKLVVGDVFRAPSNPALLCVMVGDGVQILGMSVVTILFAALGFMSPASRGTLITGILFFYMILGIAAGYVSVRMWRTIGFGEQKGWVSIAWKAACFFPGISFLILTTLNFLLWGSHSTGAIPFALFIILILLWFCISLPLTLVGGYFGAKAPHIEYPVRTNQIPREIPQQKYPSWLLVLGAGTLPFGTLFIELFFIMSSIWMGRVYYVFGFLFVVLILLVVVCAEVSLVLTYMHLCVEDWKWWWKSFFASGSVAIYIFLYSVNYLVFDLKSLSGPVSATLYLGYSLFMVLAIMLSTGTIGFLSSFWFVHYLFSSVKLD

R-73

>Glyma.14G020000
MAPLTFSFFFILISYISSSSEAGSIGINYGRIANDLPTPAKVVELLKSQGLNRVKLYDTDATVLTAFANSGMKVVVAMPNELLANAAAEQSFTDAWVQANISSYYPATQIEAIAVGNEVFVDPNNTTKFLVPAMKNVHASLVKYSLDKNIKISSPIALSALQNSFPASSGSFKTELLEPVIKPMLDFLRQTGSYLMVNAYPFFAYAANSDKISLDYALFKENPGVVDSGNGLKYTNLFDAQIDAVFAAMSAVKYDDVKIAVSETGWPSAGDSNEIGASPDNAASYNGNLVKRVLSGSGTPLKPNESLDVFLFALFNENQKTGPTSERNYGLFYPSQKKVYDIQLTAEAPPSGVGKSQVPVSGDVTTSSKGQTWCVANGGSSEKKLQNALNYACGEGGADCTPIQPGATCYDPNTLEAHASYAFNSYYQKMARASGTCYFGGTAYVVTQPPKYGNCEFPTGY

R-74

>Glyma.13G349300
MAISPILLLAILSLIYGNGVLPIEATHHVYRNLQTLSSDSSDQPYRTAYHFQPRKNWINDPNGPMRYKGLYHLFYQYNPKGAVWGNIVWAHSISNDLVNWTPLDHAIYPSQPSDINGCWSGSATILPRGKPAILYTGINPNKHQVQNLAIPKNMSDPLLREWVKSPKNPLMAPTISNNINSSSFRDPTTAWLGKDGYWRVLIGSKIHTRGMAILYKSKNFVNWVQAKQPLHSAEGTGMWECPDFYPVLDNKGPSTIGLDTSVNGDNVRHVLKVSLDDTKHDHYLIGTYDIAKDIFTPDNGFEDSQTVLRYDYGKYYASKTIFEDGKNRRVLLGWVNESSSVPDDIKKGWAGIHTIPRAIWLHKSGKQLVQWPVVELESLRVNPVHWPTKVVKGGEMLQVTGVTAAQADVEISFEVNEFGKAEVLDKWVDPQILCSRKGAAVKGGLGPFGLLVFASRGLQEYTAVFFRIFRYQNKNLVLMCSDQSRSSLNKDNDMTTYGTFVDMDPLHEKLSLRTLIDRSVVESFGGEGMACITARVYPTIAINKKAQLYVFNNGTAAVKITRLSAWSMKKAKIN

R-75

>Glyma.12G096300
MYFRIYGVTILLFLLPTLTFSLSQGQINSNSILVALLDSHYTEVAELVEKAMLLQTLENTVMNNNITIFAPRNEALERDLDPDFKRFLLEPRNLQSLQTLLLSHIVPKRIIKPEYLTGTGNPGRSNNPTRHRTLAPNHHLTLQSLNLTHWNVDSSRVMNPDSVTRPDGVIHGIDTLLIPRSVQDEFNRRRNLISIAAVKPEPSPEVDPRTHRLKKPAPASPAGSPPALPIYDAMAPGPSLAPAPAPGPGGPRHHFNGEKQVKDFIHTLLHYGGYNEMADILVNLTSLATEMGRLVSEGYVLTVLAPNDEAMAKLTTDQLSEPGSPEQIMYYHLIPEYQTEESMYNAVRRFGKVRYDTLRLPHKVTAQEADGSVKFGHGDTSAYLFDPDIYTDGRISVQGIDGVLFPPQEEDAGPVTRSKPAKVVVKQRRGKLLETACWMLGSFGQNSRFISCQ

R-76

>Glyma.01G171100
MVKSINFLLLLSLLAFAPLCHCKKKIGGYLYPQFYDGSCPRAQEIVQSIVAKAVAKEPRMAASLLRLHFHDCFVKGCDASVLLDSSGTIISEKRSNPNRDSARGFEVIDEIKSALEKECPHTVSCADILALAARDSTVLTGGPSWGVPLGRRDSLGASISGSNNNIPAPNNTFQTILTKFKLKGLDIVDLVALSGSHTIGNSRCTSFRQRLYNQTGNGKADFTLDQVYAAELRTRCPRSGGDQNLFVLDFVTPIKFDNFYYKNLLANKGLLSSDEILLTKNKVSADLVKQYAENNDIFFEQFAKSMVKMGNITPLTGSRGEIRKNCRRINK

R-77

>Glyma.12G064300
MARSLFLCSLLILAVTTFSSPTGALILTLVNNCNYTVWPGIQPNAGHPVLAGGGLTLRSLTHQSIPVPDAHWSGRVWARTGCSYSGTAFSCASGDCGGRLQCNGAGGAAPATLAQLEVHHGSNDYASYGVSLVDGFNVPMTFTPHEGKGVCPVVGCRNDLLATCPRVLQHRVPAGHGPVVACKSGCEAFHTDELCCRNHFNNPNTCKGSIYSSFFKHACPATFTFAHDTPSLMHQCSSPRELKVIFCH

R-78

>Glyma.04G034700
MAKSSADDAELRRACEHAIEGTKHKVVLSIRTVKTHGTWGKTAKLGRQMAKPRVLALCTKAKVQRTKAFLRVLKYSNGGVLEPAKLYKLKHLSKMEVATNDPSGCTFTLGFDNLRSQSVAPPQWTMRNVDDRNRLLFGILNICKDILGRLPKVVGIDVVEMALWAKENTPAVSTQNNQQDGTSVESTAAETELKVYVEKELVSQAEEEDMEALLGTYIMGIGEAEAFSERLKRELQALEAANVHAILESEPLIDEVLQGLEAASNCVEDMDEWLGMFNVKLRHMREDIESIETRNNKLEMQNVNNRTLIQELDKLLEQLSIPSEYSACLTGDSFDEAQMLQNMEACEWLTTAMRGLEVPNIDPTYAKMRAVKEKRGELQIIKSVFVGKASEYLRSYFASFVDFMLNDKNYFSQRGQLKRPDHADLRYKCRTYARLLKHLKILDKNCLGPLRKAYCSSLNLLLRREAREFANELRASAKTSKTPTVWEGFMGSGQNVNSADTAAVSEAYAKILTIFIPLLVDESSFFAHFMCFEVPSLVDGNKTGHNDQKNDDDLGIMDIDENDSKSGKNSVDFAALNESLQDLLDGIQEDFAAVVDWAHKIDPLCCISMHGTTERYLSGQKADAAGFVRILLGDLESRISMQFSRFVDEACHQIERSERNARQTGVLPYIPRFASLATRMEQYVAGQSRDLVDQAYTKFVSIMFVTLEKNAQTDPKYADIFLIENYAAFQNSLYDLANVVPTLAKFYHQASEAYEQACTRHISVIIYYQFERLFQFARKIEDLILNNVAPEEIPFQVGLSKADLRKTLKSSLSGVDKSIAAMYKKLHKNLTSEELLPSLWNKCKNDFLDKYETFVQLVARIYPAESVPSVAELRDLLASM

R-79

>Glyma.10G217200
MSSDSDEDELLQMALKEQAQRDVNYGGKSSSNSRKPVANYVQPLKKPAPPPKQSQGKGRVADDDDDSEIEMLSISSGDEDNVQDPVAASRTKAAAAGRPVREDDRTWDGEEPSRWKHVDEAELARRVREMRETRSAPAPQKFVASKFEKEGSAVGRKGLTYLQSFPRGMECVDPLGLGIIDNRTLRLITESAHSSPKTDKDIQDGNLREKLLYFSENFDAKMFLSRIHSNTSAADLEAGALALKTDFKSRTEQRKQLVKDNFDCFVSCKTTIDDIESKLRQIEDDPEGSGTSHLFNIIQDVSLQANRALKPLFERQAQAEKIRTVQGMLQRFRTLFNLPSTIRGSISKGEYDLAVREYKKAKSIVLPSHIQVGILKRVLEEVEKVMNDFKTMLFKSMEDPQIDPTNLENTVRLLLDLEPESDPVWHYLNIQNQRICGLLEKCTLDHEARMENLHNELRERALSDARWRQIQEDMNESSDINNSPIGNTYPAVQSHPSDLTGEEVDGLRGRYIHRLTAVIIHYIPAFWKVALSVFSGKFAKSSQVPTDSNSNSSANKIEEKAGDGKYSSHSLDEVAAMICSTISLYGVKVTSIFHDLEESNVLQFYMSEAIEDISKACATLELKEAAPPIAVASIRTLQSEIIKIYILRLCSWMRASVEEVSKDVTWVIVSILERNKSPYAISFLPLTFRSVVASAMDQINSMLRSLRNEATKSEDMFMQLQEIQESVRLAFLNCFLDFAGSLERIGFELGQHRSDEEGSQLPNGYTHELENAPSGLRGGVIDPHQQLLIVLSNIGYCKNELSCELYDKYRHIWQHSRGKDEGNSDLEYLVNSFSALEAKVLEQYTFAKANLIRSAAMNYLLHSGIQWGAAPAVKGVRDAAVELLHTLVAVHAEVFAGAKPLLDKTLGILVEGLIDTFISIFHENEATDLSALDTNGFCQLMLELEYFETILNPYFTSDARDSLKSLQGLLLEKATESVTDAVDNPGHNRRPTRGSEDALADDKQQGTTVSPDELISLAQQYSSEFLQSELERTRINTACFAESFPLDSVPEPAKSAYSPFRNSMDSPSRNHRGTYNTGASSFSRHRH

R-80

>Glyma.03G026900
MMAEDLGVEAKEAAVREVAKLLPLPELLQSISSIKADYISRQQANDAQLSTMVAEQVEQSQAGLKSLSFSERTINQLRENFVSIENLCQECQTLIDNHDQIKILSNARNNLNTTLKDVEGMMSISDEAAEARDSLSDDKEIVNTYERLTALDGKRRFALAAAGSHKEEVGRLREYFEDVDRTWETFEKTLWGHISNFYKLSKESPQTLVRAVRVVEMQEILDQQIAEEAAEAEGDGAMASVANPRNTGIKSTSAMASSKNLTQQKLKVQGKGYKDKCYEQIRKTVEGRFNKLLNELVFEDLKAALEEARAIGEELGDVYDYVAPCFPPRYEIFQLMVNLYTERFIQMLRLLSDRANELTNIEILKVTGWVVEYQDNLIGLGVDESLAQVCSESGAMDPLMNSYVERMQATTRKWYLNILEADRTQPPKKTEDGKLYTPAAVDLFRILGEQVQIVRDNSTDLMLYRIALATIQVMIDFQAAEKKRLEEPASEIGLEPLCAMINNNLRCYDLAMELSNSTIEALPQNYAEQVNFEDTCKGFLEVAKEAVHQTVSVIFEDPGVQELLVKLYQKEWSEGQVTEYLVATFGDYFGDVKMYIEERSFRRFVEACLEETVVVYVDHLLTQKNYIKEETIERMRLDEEVIMDFFREHISVSKVENRVSVLSDLRELASAESLDTFTLIYTNILEHQPDCPPEVVEKLVGLREGIPRKDAKEVIQECKEIYENSLVDGRPPKAGFVFRRVKCLTATKGGLWRKLT

R-81

>Glyma.10G207900
MGIFDELPLPSEKAYLREDLSRIDESWVAARFDSLPHVVHILTSKDRDAAAQFLKEQSDIIEEVVDEVVHSYHSGFNRAIQNYSQILKLFSESTESISVLKVDLGEAKRRLSARNKQLHQLWYRSVTLRHIISLLDQIEDIAKVPARIEKLIAEKQFYAAVQLHVQSILMLERGLQTVGALQDVRSELTKLRGVLFYKILEDLHAHLYNKGEYSAAGSSLLENDDEIPTTTAVALAAHNSQPLSRRTRSLKGDNQNNLQIDGSYRPASVDGGSFDGHDEADLNEEATLDGNMATTRINGNDIPKDSNNALRQMPTWLSNSTPDEFLETIRKSDAPLHVKYLQTMVECLCMLGKVAAAGAIICQRLRPTLHEIITSKIKAHAELLNSSRSSIGQDSQAGTGNLHFIKGQLESYQLPKQKRKNGISIAGTLLAVSPVSPLMAPGGKAQVAAKELLDSILDAVVRIFENHVIVGELLEAKASQHADINTPKSLPVDVNWNPDSEASQVTGGYSIGFSLTVLQSECQQLICEILRATPEAASADAAVQTARLASKVPSKDKRDGSEDGLTFAFRFTDASISIPNQGVDLVRQGWSRKGPNVLQEGYGSAAVLPEEGIYLAASIYRPVLQFTDKVASMLPTKYSQLGNDGLLAFVENFVKDHFLPTMFVDYRKGVQQAISSPAAFRPRAHVATTYTSSIEKGRPVLQGLLAIDHLTKEVLGWAQAMPKFSNDLVKYVQTFLERTYERCRTAYMEAVLEKQSYMLIGRHDIEKLMRIDPSSAYLPNLLGQLNVESNSSDAETIEAELELSELLLSLRPIKQENLIHDDNKLILLASLSDSLEYVADSIERLGQTTQRASNHVGGKYHHSHSDSAPTRSLVSFAQDYRKLAIDCLKVLRIEMQLETVFHMQEMANTEYLDDQDAEEPDDFIISLTAQITRRDEEMAPFISNAKRNYIFGGICGVAANASVKALADMKSINLFGVQQICRNAIALEQALAAIPSINSEAVQQRLDRVRTYYELLNMPFEALVAFITEHIHLFTPVEYAKLLNVQVPGREIPPDAQDRLSEILSV

R-82

>Glyma.16G014200
MREPRDGANNKPSKAAAAAAASPPQSFPLILDVDDFKGDFSFDALFGNLVNELLPTFKLEESESDGGDALPNGHLRVPSTDGSKYSQGIVSPLFPEVEKLLSLFKDSCKELLELRKQIDGRLYNLKKDVSVQDSKHRKTLAELEKGVDGLFDSFARLDSRISSVGQTAAKIGDHLQSADAQRETASQTIELIKYLMEFNSSPGDLMELSPLFSDDSRVADAASIAQKLRSFAEEDIGRHGIPVPSAMGNATASRGLEVAVANLQDYCNELENRLLSRFDAASQKRELTTMAECAKILSQFNRGTSAMQHYVATRPMFIDVEIMNADTKLVLGDQAAQASPSNVARGLSSLYKEITDTVRKEAATITAVFPSPSEVMSILVQRVLEQRITALLDKLLEKPSLVNLPSVEEGGLLLYLRMLAVAYEKTQELARDLQAVGCGDLDVEGLTESLFSSHKDEYPEYEQASLRQLYKVKMEELRAESQQISDASGSIGRSKGASVVSSQQQISVTVVTEFVRWNEEAISRCNLFASQPATLATHVKAVFTCLLDQVSQYIADGLERARDSLTEAANLRERFVLGTSVTRRVAAAAASAAEAAAAAGESSFRSFMIAVQRSGSSVAIIQQYFANSISRLLLPVDGAHAAACEEMATAMSSAEAAAYKGLQQCIETVMAEVERLLSAEQKATDYRSPDDGMAPDHRATSACTRVVAYLSRVLESAFTALEGLNKQAFLTELGNRLHKVLLNHWQKYTFNPSGGLRLKRDITEYGEFLRSFNAPSVDEKFELLGIMANVFIVAPESLSTLFEGTPSIRKDAQRYNYIGSVNAIIYVLWSCYYY

R-83

>Glyma.02G160300
MDIKTMRRGVMENSDGGEDMVLANLIANGDDVGPLVRLAFERGRPEGLLHQLIYVVKQKEAEIEEMCKTHYEEFILAVDELRGVLLDAEELKSELQSDNFKLQQVGSALLAKLEELLESYSVRKNMTEAIEMSKNCIQVLELCVKCNSHISEGQFYSALKTLDLVEKSCTQNIPAKAIKMLIESRIPVIKLHIEKKVCSEVNEWMVEIRSSAKNIGETAIGHAVTVRQRDKEMLEQQRKAEEQSISGLGDLAYTLEAEELEEDSVLQFDLTPLYRACHIHDCLGIQEKFREYYYTNRLLQLNSDLEITSAQPFVESYQTFFAQIAGFFIVEDRVLRTTGGLLVADQVETMWETAVAKMSSLLEEQFSCMESAPHLLLVKDYVTLFGSTLRQYGHEIGTLLDVLNSSCDKYRLLFLEECQQQILDVFGNDPYDQMEIKKQSDYENIVLSFNLQTSDIMPAFPYTAPFSSMVPNACRIVRSFIKGSVDYLSYGIHVNFFDVVRKYLDKFLIDVLNVMLLEKINSGNVTVPQLMQIAANIAVLERACDFYLRHAAQLCGIPVRSVGRPLGTLTAKVILKTSREAAFIALQSLVNTKIDEFMTLTESVNWTPEETNENGNDYIHEVIIYLDSILSPAQQILPLDAVYRVGSGAFEHISNSIVAAFSSDNVKRFNANAVINVDYDLQIIENFAEERFYSAGLGEIDDEVSFKICLVEARQLVNLLLSSHPENFLNPDIWEKNYYTLEIKKVAAILDKFKDSPDGIFGSLANKNAKQSARKKSMDVLKKRLKDFN

R-84

>Glyma.14G001400
MWEKGVLRLILIKGRVSTTMLSSKPPRRKVVPANGDDSADKLDQLLLSSAICNNEDLGPFIRKTFASGKPETLHHHLRHFARSKESEIEEVCKAHYQDFILAVDDLRSLLSDVDSLKSSLSDSNSRLQHVACPLLSSLDAFVETRNVSKNVNLAIDSVRTCVKLMEVCTRANRHLADDNFYMALKCVDAIEREYLDQTASSTLRRMLEKKIPEIRSYIERKVNKEFGDWLVEIRVVSRNLGQLAIGQASAARQREEDLRIKQRQAEEQSRLSVRDCIYALEEEEEDGIVAGGIGEDGGGAAGFDLTSLYRAYHIHQTLGLEDRFKQYYFENRKLQLTSDFQVSSMTPFLESHQTFFAQIAGFFVVEDRVLRTGGGLISKMEVENLWDIAVSKMCSVLEDQFSRMQTANHLLLIKDYVSLLGVTLRRYGYPIDALLDVLSKHRDKYHELLLSDCRKQIAEAVVADKFEQMLMKKEYEYSMHVLSFQIQTSDIIPAFPYVAPFSSTVPDCCRIVRSFIEDSVSFMSYGGQLEFYEVVKKYLDRLLSEVLDEALVKLINTSINGVSQAMQMAANMVVLERACDFFFRHAAQLSGVPLRMVERSRRQFPLRKARDAAEDMLSGLLKAKVDGFMTLIENVNWMCDEAPQSGNEYVNEVIIYLEILVSTAQQILPSQVLKRVLQEVFAHISEKIVGTLVSDSVKRFNVNAINGIEVDIRLLESFSDNQASLFSDGDVDVLKASLASSKQLINLLLSNHPENFLNPVIRERSYNTLDHKKVVIVSEKLRDPSDRLFGTFGSRGARQNPKRKSLDTLIKRLRDVS

R-85

>Glyma.07G222800
MESSEEEEDFPSIESIIPQSKVDSLYQSQTEKGIRKLCCELLDLKDAVENLCGNMHSKFLAFLRISEEAVEVKHELIELQKHISAQGILVQDLMTGVCRELDEWNQSSNDVSEIQQEPELPELLEPLPNERNDKKILFLETIDVLLAEHKFEETLEALEALEALDAEEKNSAELKGSGNNSSDDVSSYKSALLERKAMLEDQLVGIAEQPSVSFPELKTALNGLTKLGKGPLAHQLMLKFYQSHLQKRIEALLPSSSLCPETFPSTLSKIVFSVISLTIKESALIFGDNPVYTNRIVQWAEWEIEYFVRVVKENAPSSETVSALRAASIGIQASLNYCSILESQGLKLSKLLLVLLRPSIEEVLESNFRRARRVVLDMAESAECCPLSPQFASSLSAIASSSNSMLVESGMRFMHIVEEILEQLTPMASLHFGGNVLNRILQLFDKYMDALIRALPGPSDDDNLPELKEVVLFRAETDSEQLAILGIAFTILDELLPNAVLSRWMLQSESKAKEPNSGVTENVSFNTNATVELKEWRKHLQHSFDKLRDHFCRQYIVTFIYSREGKTRLNAHIYLSDNRDDLYWDSGPLPSLPFQALFAKLQQLATVAGDVLLGKEKIQKMLLARLTETVVMWLSDEQEFWGVLEDKSAPLKPLGLQQLILDMHFTVEIARFAGYPSRHIHQIASAITARAIRTFSARGIDPQSALPEDEWFVETAKSAINKLLLGVSGSEASDTDEDHIIDHHDEVVSDSDTVSSLSSMESTESFASASMAELDSPSNLSDPDN

R-86

>Glyma.20G194100
MGIAVGGVDLLSEKAAMMRECLQKSETITDNVVTILGSFDHRLSALETAMRPTQIRTHSIRKAHENIDRTSKVAEVILAHFDQYRQAEAKILKGPHEDLENYLEAIDKLRSNIQFFGSKKGFKSSDGIVVHANNLLAKAISKLEDEFRQLLLSYSKPVEPERLFDCLPNSMRPSSPGHEGDPSGKNHHSESHNNNAEAVVYTPPALIPPRFLPLLHDLAQQMVEAGHQQQLLKIYRDARSNVLEESLQKLGVEKLNKDDVQKLQWEILEAKIGNWIHFMRIAVKLLFAGERKVCDQIFEGFDSLSEQCFAEVTTNSVSMLLSFGEAIAKSKRSPEKLFVLLDMYEIMQELHSEIETLFKGKACSAIREAATSLTKRLAQTAQETFGDFEEAVEKDATKTAVTDGTVHPLTSYVINYVKFLFDYQSTLKQLFQEFEGGDDSSQLASVTVRIMQALQTNLDGKSKQYKDLALTHLFLMNNIHYIVRSVRRSEAKDLLGDDWVQRHRRIVQQHANQYKRNAWAKILQCLSIQGLTSSGGGSGTAGGDSGTGSSSGASRAIVKDRFKAFNIMFEELHQKQSQWTVPDSELRESLRLAVAEVLLPAYRSFVKRFGPLVESGKNPQKYIKYSAEDLDRMLGEFFEGKNMSETKR

R-87

>Glyma.02G232100
MSENGEEKLLAVARHIAKTLGHNNTMSDDIFQILSNFDGRFSRENLSEKGADADPRGCAALDHSLKTLDRRISLYVSYDRPIWSDAADSAAFLDAVDKLVAVVAEWNHLASDKAVAACLVRAEDMLQHAMFRLGDEFRSLMERGGESFGLTRSYWNGESTENLPFESDEDEEEEEARNGGGDKEEQIPVALPVTGFDIVIDALPSGTINDLHEIAKRMVAGGFGKECSHVYSSCRREFLEESVSRLGLQKLSIEEVHKMTWQDLEGEIEKWIKASNVALKILFPSERRLCDRVFFGFASASDFSFMEVCRGSAIQLLNFADAVAIGSRSPERLFRILDVFETLRDLIPEFEALFSDQFSVSLRNEAITIWRRLGEAIRGIFMELENLIRRDPAKMAVPGGGLHPITRYVMNYLRAACRSRQSLEQVFEDYGLKEYTKLEDRVPSSSSLSVQMDWIMELLESNLEAKSRIYKDPALRYVFLMNNGRYIVQKTKDSELGTLLGDDWIRKHAAKVRQFHVHYQRCSWTKVLGILKLDSNGSSLPPNGLAKSMKETLKLFNTVFEETCREHSSWFVFDEQLREEIRISLEKILLPAYGNFVARFESVAELGKNADKYIKYGTEEIQATLNGLFQGSSGSTGSRK

R-88

>Glyma.07G041100
MESLPLETAEKIILRWDSTASEEARDKMIFSGGGDRDEADLYLQAVDEIQRSLSSVSVSVSSDKVNSAIQIAMARLEDEFRNILISHTNPFDPSSEDEPSQTLDSLSTTSSPKHPLTNEETTEEESDSNHNTISTPLFRFNSDGAASSVRSSVNSVNSSSYRSTSSIREIDLIPSDAVYDLRCIAERMVSSGYLRECIQVYGSVRKSSVDASFRKLQIEKLSIGDVQRLEWEQLENKIRRWIRAAKVCVRTLFASEKKLCEQIFDGVGTSIDDACFMETVKGPAIQLFNFAEAISISRRSPEKLFKILDLHDALTDLMPDIDVVFDSKSSESIRVQAAEILSRLGEAARGILSEFENAVLREPSRVAVPGGTIHPLTRYVMNYISLISDYKVTLNELIVSKPSTGSRYSGDPGIPDMDLSEFEEKTPLDVHLIWIIVILQFNLDGKSKHYRDASLAHLFVMNNVHYIVQKVRGSPELREMIGDDYLKKLTGKFRQAATSYQRATWVRVLYCLRDEGLHVSGGFSSGVSKSALRERFKAFNAMFEEVHRTQAVWLIPDLQLREELRISISEKLIPAYRSFLGRFRSHIESGRHPENYIKYSVEDLEDAVLDFFEGIPVSQHLRRRAE

R-89

>Glyma.16G009800

MSHSHRAEDKYFFAHTQRNFTQQNSLSFSHFLIMESLPLETAEKIILRWDSTASEDARDKMIFSGGVDRDEADLYLQAVDEIQRSLSSVSVSSSDKVNSAIQIAMARLEDEFRNILISHTIPFDPSTSEDDPSQTLPDLLPSSTSSPKHPLTNLEQDSTETEPETTNTTTTTTPLLRFNSDGAASSVHSSVSSVNSVNSSSYRSTSSIREIDLIPSDAVYDLRCIAERMLSSGYLRECIQVYGSVRKSSVDASFRKLHIEKLSIGDVQRLEWEQLENKIRRWIKAAKVCVRTLFASEKKLCEQIFDGVGTSIDDACFMETVKGPAIQLFNFAEAISISRRSPEKLFKILDLHDALTDLMPDIDVVFDSKSSESIRVQAAEILSRLGEAARGILSEFENAVLKEPSRVPVPGGTIHPLTRYVMNYISLISDYKVTLNELIVSKPSTGSRYSGDVGIPDMDLSEYEEKTPLDVHLIWIIVILQFNLDGKSKHYRDASLAHLFIMNNVHYIVQKVRGSSELREMIGDDYLKKLTGKFRQAATRYQRETWVKVLYYLRDEGLHASGGFSSGVSKSALRDRFKTFNSMFEEVHRTQAVWLIPDSQLREELRISISEKLIPAYRSFLGRFRSYIESGRHPENYIKYSVEDLEYAVLDFFEGIPVSQHLRRRSE

R-90

>Glyma.08G245400
MAVEESEPVIGELEREENLIAAVRHIVKALGPNKTLTSDAKKILADLGTRLSSMSVPSEKEEGKQGQGKDDGDNCDGGGDLYDEDDDDDEGISAIEEKLNVIQEKIMRWEEDQSMIWDLGPEEASEYLNAANEARRLIEKLESLNLKKEDQEYKFMQRAYSVLQTAMARLEEEFRNLLIQNRQPFEPEYVSFRSSEEDAVDENSIVSLGDESVEESLQRDSVSRASEEHIIYLVHPAVIPDLRCIANLLFASNYVQECSNAYIIVRRDALDECLFILEMERLSIEDVLKMEWGTLNSKIKRWIWAVKIFVRVYLASERWLSDQLFGEGEPVGLSCFVDASKASILQLLNFGEAMSIGPHQPEKLFRVLDMYEVLQDLMPDIDALYSDEVGSSVKIECHEVLKRLGDCVRVTFLEFENAIATNVSSTPFVGGGIHPLTKYVMNYLRTLTDYSDILNLLLKDQDEDAISLSPDMSPGTEEDSRSQGSPGRVSSMALHFRSIASILESNLEEKSKLYKEVSLQHLFLMNNLHYMAEKVKGSELRLIHGDEWIRKCNWKFQQHAMKYERASWSPILNLLKDEGIHVPGTNSVSKSLLKERLRSFYLGFEDVYRIQTAWIIPDIQLREDLRISISLKVIQAYRTFVGRHNSHISDKIIKYSADDLENYLLDFFEGSQKWLQNPHRR

R-91

>Glyma.05G047800
MATTTTSLGGGVGVGGDDRVLATAQQIVKSLRAAKEDREDMLMIFSAFDNRLSGISDLINGDDSKSSDEEDLDRFEAAEKVILADASLSGEPSRQSTSLFNPPNNPAEYFSAVDEIIHWMEQFSIAPPPSSALGRTVHVIADRAENAIQLAMSRLEEELRHVLICNTIPLDAVSRYGSIKRVSLSFGSHDGAIDDSPLESFGEVDSSRFHDRGASLGDDLFVDLVRPEAVQDLREIIDRMVRSGYERECLQVYSSVRRDALDECLIILGVERLSIEEVQKVEWRSLDEKMKNWVQAVKVVVGVLLSGEKRLCDGLFGDLDDLKEICFNETAKGCVMQLLNFGEAIAICKRSPEKLFRILDMYEALRDAMPDLQAMVSDEFVIGEANGVLSGLGEAAKGTFAEFENCIRNETSKKPVITGDVHPLPRYVMNYLRLLVDYGDPMDSLLELSEEDLYRFKNDLGGDGSQLEAMSPLGQWILLLMSELEYNLEEKSKLYEDSAMQQVFLMNNLYYLVRKVKDSDLGRVLGDNWIRKRRGQIRQYATGYLRASWSKALSCLKDEGIGGSSNNASKMALKERFKSFNACFEEIYRVQTAWKVPDDQLREELRISISEKVIPAYRSFVGRFRIQLEGRHVGKYIKYTPEDLETYLLDLFEGSPAVLHHIRRKST

R-92

>Glyma.17G197600
MEASRIENLIRAKKSLKISLEKSKSVGLALEKAGPRLDEIRVRLPSLGSAVRPIRAEKDALAAVGGHINRAVGPAAAVLKVFDAVHGLEKSLLSDPRTDLAGYLSVLKRLQEALRFLGDNCGLAIQWLEDIVEYLEDNSVADKVYLANLKKELKNLRESQHGELDGGLLDAALGKLEDEFRLLLSENSVPLPMASASGDQACIAPSPLPVSVVHKLQAILGRLIANDRLDRCVGIYVEVRSSNVRASLQALNLDYLEISLSEFNDVQSIEGYIAQWGKHLEFAVKHLFEAEYKLCNDVFERIGLDVWMGCFSKIAAQAGILAFLQFGKTVTESKKDPIKLLKLLDIFASLNKLRLDFNRLFGGAPCVEIQNLTRDLIKSVIDGAAEIFWELLVQVELQRPNPPPMDGNVPRLVSFITDYCNKLLGDDYKPILTQVLIIHRSWKRQSFQEKLLVNEILNIVKAVEQNVETWIKAYDDPILSNFFAMNNHWHLCKHLKGTKLGELLGDSWLREHEQYKDYYSTIFLRDSWGKLPGHLSREGLILFSGGRATARDLVKKRLKKFNEVFDEMYAKQTSWIMPERDLREKTCQLIVQAVVPVYRSYMQNYGPLVEQDASSTKYAKYTVQKLEEMLLCLYRPRPVRHGSLRSSTFSAKYGNGVPDLRRTASAVV

R-93

>Glyma.11G144600
MPRKGMRSIFFTSTPTASLPPPSRQRTFSDSLMDENIETAEKLITKWDDSKVTTTTQLFSGTRQEAKQYLNAVKGLQSAMQYLVAQDSTSSTLVRAQFLMQLAMKTLQKEFYQILSSNREHLDPETVSTRSSVDHRSSVSDYDDEISITEDEFRVSETERVSMLAMEDLKAIAECMISSGYGKECVKVYIVMRKSIVDEALYHLGVEKLNLSQVQKLDWEVLELKIKSWLKAVKVAVGTLFNGERILCDHVFAADSGKRIAESCFAEITKDGAVSLLGFPEMVAKCKKSPEKMFRILDLYEAISDYWPQIEFIFSFESTVNIRTQTVTSMVKLGDAVRTMLTDFETAIQKESSKKPVPGGGVHPLTRYVMNYLTFLADYSGVLVDIIADLPQSPLPESYYRSPMREENPPASELSERIAWIILVVLCKLDGKAELYKDVAHSYLFLANNMQYVVVKVRKSNLGFLLGEEWLAKHELKVREYTSKYESVGWSAVFSSLPENPAAELTAEQARACFVRFDAAFHEACKKQASWVVSDPKFRDEIKDSIASKLMQKYSVFFEKNRVGSKSVRDFLPDDIGKYLSNILCDGDSVSVSSHSSSTTSASHRSNRR

R-94

>Glyma.20G188500
MRVSSPDTAAPSLHADEHRIFTLGSRDAESLFRSKPIAEIRKTEAATRKQIEDKKEELRQLVGNRYRDLIDSADSIVLMKVSCNGISSNIAAVHGRIRSLSQSQSQSQTKLHSQSRAWTYGAACRVKYLVDTPENIWGCLDEGMFLEAASRYVRAKNVHHHLFVDSDDQKKKFLSNFAMLQHQWQIVESFRAQISQRSRDRLLERGLAISAYSDALAAVAVIDELEPKQVLSLFLESRKSWISQILGNAGPGDASSLVVSILCDVLGIIQVTVGQVGELFLQVLNDMPLFYKVILGSPPASQLFGGIPNPDEEVRLWKSFRDKLESIMVMLDKRYIADTCFAWLRGCVSKISGRNLIDVVGSGQDLACAEKSIRETMESKQVLQESLEWLKSVFGSEIELPWSRIRELVLEDDSDLWDEIFEDAFVGRMKAIIDLRFRELTGAVDVLNSISAIGDFCTKLEDVQGYLNRPSTAGGVWFLESNARKTGVASGFKVQPEESEFQYCLNAYFGPEVSRIRDAVDVSFQSIFEDLLSFLESPKASRRLKDLAPYLQSKCYECVSSILMTLKKELDSLYAPTENGKVPTAVTVEKSLFIGRLLFAFQNHSKHIPLILGSPRFWANGNASAVGKLPTLVKQSRFGSDSAICDSPGRQTSLGSKRQNSSAVSALLGVREGASHELEELNKTIGDLCIRAYNLWILWISDELSAIVSQDLKQDDALSLSTPWRGWEDIIVKQDQSDENQSDMKISLPSMPSLYIISFLFRACEEVHRVGGHVLDKKILHKLASRLLEKVTGIFEDFLSTAESGVHQVSEKGVLQVLLNFKFATDVLSGGDSNMVGELSSNPKAKLPGRRKQDQSLTTSAIRERSNQLLNRLSQKLDPIDWLTYEPYLWENERQSYLRHAVLFGFFVQLNRMYTDTVQKLPTNSESNILRCSTVPRFKYLPISAPALSSRGTKKAFTPSSSEISSRSSWNSITNGELSQKINLDDSSSLGVAAPLLKSFMQVGSRFGESTFKLGSILTDGQVGIFKDRSAAAMSSFGDILPAHAAGLLSSFTAPRSDS

R-95

>Glyma.05G047300
MADPIPAPPRSATELFSDPLDAHPLWFKPASFLSPDFDSESYISELRTFVPFDTLRSELNSYLSSLNHELIDLINRDYADFVNLSTKLVDVDAVVVRMRAPLVELRDKIEQFRGSVEVSLVAIKSRLRQRSEVASARETLELLLDAFHVVSKVEKLIKELPSVPTDWSNGDVNLSERNNFSNGVSVQHVENEMSIRETQSMLLERIASEMNRLKYYVTHAKNLPFIENMEERIQNASLTVYASLGHCFVNGLENRDATAIFNCLRAYAAIDNTKNAEEIFRATVVAPLVQRIIPHGSSAVVAGSSGDGLENDYQLIKECIDKDCKFLLEISSAENSGLHVFDFLANSILKEVLSAIQKGKPGAFSPGRPTEFLKNYKSSLDFLAYLEGYCPSRLSVAKFRSEAIYTEFMKRWNIGVYFSLRFQEIAGSLDSVLTTSSLVPVLNSDAGEANYQGLTLKQSVTLLESLRSCWREDVLVLSCSDRFLRLSLQLLSRYSSWLSSGLTARKNHNTSTSPGCEWAVSAVIDDFIFVIHDIRYLEEQVRGDYLQHVLKLLSSCSPDVLEPIRQSILLGGQSLKSLEPLVIKAVVESLVEKSVEDLRQMKGITATYRMTNKPLPVRPSPYVSGVLRPLKAFLDGERATRYLASEIRNKILLCAATEITDRYYELASDLVSVARKTESSLQKIRQSAQRRAGASSDISDNNVSDTDKICMQLFLDIQEYARNLSALGVEAVNIASYRSLWQCVAPADRQNTINL

R-96

>Glyma.13G114900
MGSRGPPQSHPNSAAISKGYNFASTWEQNAPLTEQQQSAIVSLSHAVSERPLPRKLAQENASVQDNALSVKTKDSSFDDSGAIETVMVNTNQFYKWFTDLESAMKSETEEKYQHYVNTLTDRIQTCDEILQQVDDTLDLFNELQLQHQAVATKTKTLHDACDRLLQEKQRLIDFAEALRSKLNYFDELENVATNFYSPNMNVGNENFLPLLKRLDECISYVENNPQYAESSVYLLKFRQLQSRALGMMRSHVLAVLKGASSQVQEAIRGSGGGKASISEGVEASVIYVRFKAAASELKPLLEEIESRSSRKEYGQILAECHRLYCEQRLTLIRGIVQRRISEFAKKESLPSLTRSGCAYLIQVCQLEHQLFNHFFPASSKDISSLAPLMDPLSTYLYDTLRPKLVHETNIDFLCELVDILKMEVLGEQHSRRSESLAGLRPTFERILADVHERLTFRARTHIRDEIANYIPTNEDLDYPEKLKKSAESTSEINPADDNPDIFKTWYPPLEKTLSCLSKLYRCLESAVFTGLAQEVVEVCSASIQKASKLIAKRSSQMDGQLFLIKHLLILREQIAPFNIEFSVTQKELDFSHLLEHLRRLLRGQASLFEWSRSTSLARTLSPRVLENQIDTKKELEKSLKATCEEFIMSVTKLVVDPLLSFVTKVTAVKVALSSGGQNQKLESVMAKPLKDQAFATPDKVAELVQKVRTAIQEQLPVVIEKMKLYLQNSSTRTILFKPIKTNIVEAHIQVQSLLQSEYTSEEIQTINLKSVQDLQNELDNYL

R-97

>Glyma.03G261100
MMGAIPEANGNVADEENGSSVGGSIDFGTAEAVQYVRSLTDVGAMTRLLHECIAHQRAVDVELDELLSQRTDLDRHLLQLQRSSDVLDIVNSDADYMLSNVASTSDLADQVSRKVRELDLAQSRVRNTLLRIDAIVERANSLEGVHRALEAEDYESAACYVQTFLQIDAQYKDSGSDQLQRDRLLAAKKQLEGIVRKKLSAAVDQRDHPAILRFIRLFTPLGVEEEGLQVYVGYLKKVIAMRSRMEFEQLVEMMDQQNVNFVGCLTNLFKDIVLAIEENSEILSGLCGEDGIVYAICELQEECDSRGSVILNKYMEYRQLAKLSSEINAHNTNLLAVGGGPEGPDPREVELYLEEILNLMQLGEDYTEFMISKIKALTSVDPELLPRATKAFRSGSFSKVAQDLTGFYVILEGFFMVENVRKAIKIDEQVPDSLTTSMVDDVFYVLQSCLRRAISTSNISSVVAVLSGASSLLGNEYHEALQHKTREPNLGAKLFFGGVGVQKTGTEIATALNNMDVSSEYVLKLKHEIEEQCAEVFPAPADREKVKSCLTELADSSNAFKQALNAGIEQLVATITPRIRPLLDSVGTISYELSEAEYADNEVNDPWVQRLLHAVESNVAWLQPLMTANNYDTFVHLIIDFIVKRLEVIMMQKRFSQLGGLQLDRDARALVSHFSAMTQRTVRDKFARLTQMATILNLEKVSEILDFWGENSGPMTWRLTPAEVRRVLGLRVDFKSEAIAALKL

R-98

>Glyma.14G029500
MASPAAARTPVSTGASPMQRLSTFKNPSSAAASTATTTPSSSALDSLASDPIFSAFLSPSFSSTSFSSAALSSGSPASTAEKLHHAIRLLENQLRSEVLSRHHDLLSQLSSLHHADHALSTLRSALSSLQSSVRRLRSELSDPHRSVAAKTAQLSNLHRTTELLQHSIRALRLSKKLRDLMAAADPEKLDLAKAAQLHFEILSLCDEYDLVGIDAVDEELNWVRETGDLLRSEAMKVLERGMEGLNQAEVGTGLQVFYNLGELKGTVEQVVNKYKGLGAKSVTVALDMKTISGGSGYGPGGIRGSGTPHIGGGAKAREALWHRLGNCMDQLHSIAVAVWHLQRVLSKKRDPFTHVLLLDEVIQEGDPMLTDRVWEAITKAFASQMKSAFTGSSFVKEIFTMGYPKLYSMIENLLERISHDTDIKGVLPAINLSGKEQIISAVEIFQNAFLAHCLSRLSDLVNSVFPMSSRGSVPSKEQISRIISRIQEEIETVQMDARLTLLVLREIGKVLILLAERAEYQISTGPESRQVNGPATPAQLKNFTLCQHLQDVHTRISSILKGMPSIAADVLSASLGVIYGVACDSVTALFQAMLDRLESCILQIHDHNFGVLGMDAAMDNNASPYMEELQKCILHFRSEFLSRLLPSRNSTAPGTENICTRLVQSMASRVLVFFIRHASLVRPLSESGKLRMARDMAELELAVGQNLFPVEQLGAPYRALRAFRPLIFLETSQLASSPLLQDLPPNVILHHLYTRAPEELQSPLQRNKLTPLQYSLWLDSQWEDQIWKGIKATLDDYAANVRSRGDKEFSPVYPLMLQLGSSLIEKDQTSSKS

R-99

>Glyma.01G154500
MGTSVADLAPGLSRKLKKVQESRIDTPYLLSSLNTLSSFYDDNTPQARRNLRSTIEKRALSINCEFLDASHAAQLINALREEELNENFFKALSHVQEIHANCKVLLRTHHQRAGLELMDMMAVYQEGAYERLCRERSVLFKYCAEEVANMRHNALFRRFISALTRGGPGGLPRPIEVHAHDPLRYVGDMLGWLHQALASERELVAVLLDPDTITDSGPKQFSNNSEDGSGKTESDLMFVLDRIFEGSQPSLIVSYKLSSTLEFYCYTISDLLGRETALCNTLWALKDAAQNTFFDILKGRGEKLLRYPPLVAVDLSPPPAVTEGVSVLLEIIDNYNSMMVPASGQKPAFGPVISAILDPIVQMCEQAAEAHKSKGAGHSSRRSGMSFDCGQLTKSSVDAILSNSSSVSSSLVCSYSFSLKFYRYLLP

R-100

>Glyma.12G013000

MMLDLGPFSNENFDPKKWINSACQSRHPQDSLDKHLVDMEMKLQMVSEEIAASLEEQSAAALLRVPRATRDVIRLRDDAVSLRSAVSSILQKLKKAEGSSAESIAALAKVDVVKQRMEAAYETLQDAAGLTQLSATVEDVFASGDLPRAAETLANMRHCLSAVGEVAEFANIRKQLEVLEDRLDNMVQPRLMDALSNRKVDAAQDLRGILIRIGRFKSLESQYVKVHLKPIKQLWEDFDSRERASKPANEKNEMDRTSSGGDFQSVSPAIPFSSWLPSFYDELLLYLEQEWKWCMIAFPEDYKTLVPKLLSETMMAIGSSFISRINLAIGDAVPETKALAKGLLDILAGDMQKGIKLQTKHLEALIELHNMTGTFARNIQHLFSVSDVRFLMDVLKSVYLPYESFKQRYGQMERAILSAEIAGVDLRGAVIRGLGAQGVELSETVRRMEESIPQIIILLEAAAERCINFTGGSEADELILALDDIMLQYISTLQETLKSLRTVCGVDYGSDGTFKKDMEKKDGNQNARRVDLISNEEEWSIVQGALQILTVADNLTSRSSVFEASLRATLARLSTTLSFSAFGSSLDQHQAINSSVDGEPSYGGRAALDMATLRLVDVPEKARKLFNLLNQSKDPRFHALPVASQRVASFTDTVNELVYDVLISKVRQRLSDVSRLPIWSSVEEQGSFPLPTFSAYPQSYVTSVGEYLLTLPQQLEPLAEGISNNEVNDEAQFFATEWMFKVAEGATALYIEQLRGIQYISDRGAQQLSVDIEYLSNVLSALSMPIPPVLATFQSCLSTPRNQLKIF

R-101

>Glyma.16G120600
MESATASSSESEMALPLPLASESQQPYVSELLSFTLDRLHKEPELLRVDADRIRRQMQEVAVGNYRSFIAAADALIAIRQEVSSIDNHLESLINEIPKLTSGCTEFIESAEQILEKRKMNQTMLTNHSTLLDLLEIPQLMDTCVRNGNYDEALDLEAFVGKLSTMHPKLPVIQALAAEVRLTTQSLLSQLLQKLRSNIQLPECLRIIGYLRRIGVFSEYGMRLLFLRCREAWLTGILEDLDQANPYEYLKGMINCHRMHLFDVVNQYRAIFADDTSGSEENYDGGLLFSWAMHQITSHLQTLKVMLPKITEGGSLSNILDQCMYCAMGLGWVGLDFRGLLPSLFEEAVLNLFSKNMSTAVENFQLVLDSHRWVPLPGVGFSAHTVGDENHEDVTPPSNLMEYKMETFLVDGSCVSAAMNDLRPCAPISLKHVLAQELIKGLWAVSDSLLLYNTTRVLRSNESGLFRSLCRAFIEVAYPHCATCFGRCYPGGATLVMDAKNVYDGISRLLETSSSRELPKPVNNEESKSVAENGELPKMENGETPDAKESEAVIGKDVNEEGPTLQIDQEDTNLEKSVE

R-102

>Glyma.04G166300
MESGDEINLNIESGNNGKSGGDGFVDRSKVRILLCDNDSKSSQEVFTLLLRCSYQVTSVKSARQVIDALNAEGQHIDIILAELDLPMKKGMKMLKYIAQDKEFRRIPVIMMSAQDEVSVVVKCLRLGAADYLVKPLRTNELLNLWTHMWRRRRMLGLVEKNILNYDFDLVVSDPSDANTNSTTLFSDDTDDKSKRSTNPEVGISVQQEQESTIAIAAAVEEPPDAHASECGPDVDGVNDHQTAHFSSGPKKSELRIGESSAFFTYVKASILKSNLEGVVNVDKNGATHVRMEVMHQTCAQQGVNDLQIRENGEAYESQSQDDLPSSTSIPDSLSIERSCTPPASMEVSQQKHYREENLHQGVMHPRNGTHCSELEVSGMASQQVYPYYISGVVNHVMMPSSAQMYHQKNIQDLQNHPSSAMISQYSHLPQGGPHGTGMTSFPYYPMSICLQPGQIPNPHSWQSFGNSSSSEAKLSKVDRREAALMKFRQKRKERCFDKKIRYVNRKRLAERRPRVRGQFVRKLKGANADLNGQPASIDYDEDDEEDEDDQGARDSSPEDA

R-103

>Glyma.16G163200
MAAASGERWMDRLQFSSLFWPPPLDDQQRKDQVAAYVEYIGQFTSEQFSEDIAECNYIYIRTVLYFDLCNPGVLSNTLTAVLVLHHPEHGHAVVLPIISCIIDGTLDYDKTSPPFASFISLVCPKNENEYSEQWAMACGEILRILTHYNRPIYKMERQYCEPEVSSGKSHATTNDSVDGESGHNSLMQQEKKPIRPLSPWITDILRAAPLGIRSDYFRWCSGVMGKYAAGELKPPTIVSARGSGKHPQLVPSTPRWAVANGAGVILSVCDDEVARYETATLTAAAVPALLLPPPTTALDEHLVAGLPALEPYARLFHRYYAIATPSATQRLLLGLLEAPPSWAPDALDAAVQLVELLRAAEDYACGIRLPRNWMHFHFLRAIGTAMSMRSGVAADAAAALLFRILSQPALLFPPLRQVDGVEVQHEPLGGYISSNRKQIEAASAEATIEATAQGIASVLCAHGPEVEWRICTIWEAAYGLIPLNSSAVDLPEIVVATPLQPPILSWNLYIPLLKVLEYLPRGSPSEACLMKIFVATVEAILQRTFPAESTSDHKIKTRYYCVGSASKNLAVAELRTMVHSLFLESCASVELASRLLFVVLTVCVSHEVQFNGSKKPRGENNYLVEEIIEDLQAVSESQKETKNRKMKKQGPVAAFDSYVLAAVCALACELQLFPMISRGDNHSVPNNVQNIAKPVKIDGSSHALQNGIDSAIRHTHRILAILEALFSLKPSSVGTSWSYSSNEIVAAAMVAAHISELFRRSKACMRALSVLMRYKWDDEIHSRASSLYTLIDIHRKAVASIVNKAEPLGATFIHTPICKDPCDRSKRKNQCENSRCLDPGQTSTSTSEDSSRSKFSKKSERTSYSNEASGCTFEKATTSLPFDASDLANFLTMDRHIGFNCSAQIFLRSRLAEKQQLCFSVVSLLWQKLIGSPETQPCAESTSAQQGWRQVADALCKVVSASPTKAAMAVVLQAEKELQPWIAKDDNLGQKLWRINQRIVKLITELLRNHDNLESLVIVASASDLLLRATDGMLVDGEACTLPQLKLLEATAKAVQPVIELGESGLGVADGLSNLLKVNKLSSFELEPILNVKF

R-104

>Glyma.17G066600
MGIERGGFKGFRSAWSVPPKPCDSCKLASAALFCRPDSAFLCIACDSNIHCSNKLASRHERVWMCEVCEQAPAAVTCKADAAALCVTCDSDIHSANPLAQRHERVPVEPFFDSAESIVKASAAATFGFIVPSDDGGASDAFAPDDSDAAAWLIPNPNFGSKLMDAPEIKSKEIFFSEMDPFLDFDYSNSFQNNNSAGNDSVVPVQKPSLAPPLINNHHHHQSETCFDVDFCRSKLSSFNYPSNSLSQSVSSSSLDVGVVPDGNTVSDMSYSFGRNSSDSSGIVVVSGNSVGQGATQLCGMDREARVLRYREKRKNRKFEKTIRYASRKAYAETRPRIKGRFAKRTEIDSDVERLYSPGPAVLMLDTPYGVVPSF

R-105

>Glyma.07G048500
MDAYSSGEEVVVKTRKPYTITKQRERWTEEEHNRFLEALKLHGRAWQRIEEHIGTKTAVQIRSHAQKFFTKLEKEALVKGVPIGKALDIDIPPPRPKRKPNNPYPRKTRIGSASLHSGAKDGKLNLVESSHVNQALNLEKEPLPEKHDLDEGITTVKENKDENRGAVFTLLQEVPCSSVSSANKSSITMSVPLGNPCAFKEITPSVKEVIPRDEKTESFVTIELENGTLEINDRKQTNGTSKDSTLENSDALHMKLVQNEKTDGLDCALTIDGMQGNQNYPRHVTVHVVDGNLGTNTQNPSQDMLFRDSMFQPIGGVNGQQNVFTNSAPSNTSESQNNTVRSSVHQSFLPYPPFTQHNQDDCQSFFHMSSTFSNLIISTLMQNPAAHAAASFAATFWPYANPETSANSPRCSQGGFTNRQIGSPPSVAAIAAATVAAATAWWAAHGLLPLCAPLHTSFACSPASVTAVPSMNTGEAPALKAEQEKTTLQNPPLQDQMLDPEYSEAQQAQHSASKSPAAILSDSESGDAKLNTSSKATDHETNKTIPEHLDSNKTKGRKPVDRSSCGSHTASSSDVETDALEKGEKGKEEPETPDANQLAIDFSNRRRSVSNLTDSWKEVSEEGRLAFQALFSREVLPQSFSPPHALKNKNQQMDNANNNKQNIDDKDEDPDSKKCSSNYEAMQKNLPFVENNEGLLTIGLGQGKLKTRRTGFKPYKRCSMEAKENRVGASNNQGEEQGCKRIRLEGETST

R-106

>Glyma.18G166800
MAQRSNVPMLGKSEENVSDTAHSDKAQKGQPGSKMINPNDTKENSDVVSSAGLPHSKPRVHSEDPSGKGSVRSIHELQMSREDGDPKQFTDSPARHGGSDSAYRGHGVGSADNRKRPSRQSTGSEHSIDRSPLHRQAKTPGRDSPSWEGKNSYDSSHGTPGRSRLRPPNRGDETPDKGAAVPKFGEWDESNPASADGYTHIFNKVREEKQVGAGHVPVTPNGRQYAARNQPADDKAQSCCFCWGKK

R-107

>Glyma.19G263500
MSLDSETSGSARDWFFPSPSFLRSSSSQYGRRFYSNSKPHSPPSSSTRIRHRRRVKFPRTPTNDKSQLSDTENVKSSATARNNLICLSQFRFQFALVTLTIVFLLLLLRNTHLESQVTKLQGEILGLNHRLHACHKLDTLYVTSSISQDVDPWSRENFKRNLALFFSFTLLFIPLLIFKYIGFVSKSRFSDNISEQVSLNKQIAYRVDVFLSVYPYAKPLVLLVATLLLIFLGGLALFGVTTEDLAHCLWLSWTYVADSGNHASSQGIGPRLVAVSISFGGMLIFAMMLGLVSDAISEKFDSLRKGKSEVVEQNHTLILGWSDKLGSLLNQLAIANESLGGGTVAVMAERDKEEMELDIAKMEFDFKGTSVICRSGSPLILADLKKVSVSKARAIIVLAEDGNADQSDARALRTVLSLTGVKEGLRGHIVVELSDLDNEVLVKLVGGDLVETVVAHDVIGRLMIQCARQPGLAQIWEDILGFENCEFYIKRWPQLEGMQFEDVLISFPAAIPCGIKVASYGGKIILNPDDSYVLQEGDEILVIAEDDDTYAPASLPTVWRGSLPKDFVYPKSPERILFCGWRRDMEDMIMVLDASLAHGSELWMFNDVPEKEREKKLTDGGLDINRLENISLVNREGNAVIRRHLESLPLESFDSILILADESVEDSAIQADSRSLATLLLIRDIQARRLPYVSMASQAHGGSFSKGSWIGEMKQASDKTVIISEILDPRTKNLISMSKISDYVLSNELVSMALAMVAEDRQINDVLEELFAEEGNEMHIRKADLYLCEGEELNFYEIMLRARQRREIVIGYRLANAERAVINPPVKTDRRKWSLKDVFVVITEKE

R-108

>Glyma.11G246200
MGLCSLFLVTLLLLTSFAVCQLEEFISIDCGGTSNYTDKSTGLAWISDSGIMKHGKPVEVQNPSGNKFQYQRRREFPIDSRKYCYTLVTEERRRYLVRATFKYGNLDDGDTYPQFQLYLDATKWATVSIYDASRIYAKEMIFRAPSNSIDVCMCCATTGSPFISTLELRPLNLSMYATDFEGSFFLKVAARINFGAPSEDVVRYPDDPYDRIWESDLIKRQNYLVGVAPGTERINTTKKIEIETRENPPVKVMQTAVVGTKGILSYRLNLEDFPGNARAYAYFAEIEDLPKNETRKFKLEQPYIADYSNAVVNIAENANGSYTLYEPSYMNVSLEFVLSFSFVKTRDSTQGPLLNAMEISKYMPIASKTDRQDSNFVNAFRFLSAESVLKNEGDPCVPTPWEWVNCSTTTPPRITKINLSRRNLKGEIPGKLNNMEALTELWLDGNMLTGQLPDMSNLINVKIMHLENNKLTGPLPSYLGSLPSLQALFIQNNSFSGVIPSGLLSGKIIFNFDDNPELHKGNKKHFQLMLGISIGVLVILLILFLTSLVLLLILRRKTSQQKRDEKGVSGRSSTKPLTGYSFGRDGNIMDEGTAYYITLSELKEATNNFSKNIGKGSFGSVYYGKMKDGKEVAVKTMTDPSSYGNQQFVNEVALLSRIHHRNLVPLIGYCEEEYQHILVYEYMHNGTLREYIHECSSQKQLDWLARLRIAEDAAKGLEYLHTGCNPSIIHRDVKTSNILLDINMRAKVSDFGLSRLAEEDLTHISSVARGTVGYLDPEYYANQQLTEKSDVYSFGVVLLELLSGKKAVSSEDYGPEMNIVHWARSLIRKGDVISIMDPSLVGNLKTESVWRVAEIAMQCVEQHGACRPRMQEVILAIQDASNIEKGTESQLKLSSSGGNSKPQSSRKTLLASFLEIESPDLSNSCLPSAR

R-109

>Glyma.15G222300
MGNETRKLSDEYEVSEVLGRGGFSVVRKGTKKSSSDTKTHVAIKTLRRVGTASNSNNPSGFPRPKGGEKKSTAAMMGFPTWRQVSVSDALLTNEILVMRRIVENVSPHPNVIDLYDVYEDSNGVHLVLELCSGGELFDRIVAQDRYSETEAAGVVRQIASGLEAIHRANIVHRDLKPENCLFLDVRRDSPLKIMDFGLSSVEEFTDPVVGLFGSIDYVSPEALSQGKITTKSDMWSLGVILYILLSGYPPFIAQNNRQKQQMIMNGNFSFYEKTWKGITRSAKQLISDLLIVDPSRRPSAQDLLSHPWVVGDKAKDDAMDPEIVSRLQSFNARRKLRAVAIASIWSTTIFLRTKKLKSLVGTHDLTEEEIENLRMSFKKICVSGDNATLSEFEEVLKAMNMPSLIPLAPRIFDLFDDNRDGTVDMREILCGFSSFKNSKGDDALRLCFQMYDTDRSGCITKEEVASMLRALPEDCLPTDITEPGKLDEIFDLMDANSDGKVTFDEFKAAMQRDSSLQDVVLSSLRPQ

R-110

>Glyma.19G145200
MMYPTACYKFQRSLTHSKMVTMYGIWCFITVLLFSPVAFSELCNPQDKKVLLQIKKDFNNPYLLASWNPNTDCCNWYCVQCHPETHRINSLVILSSVPQTNLSGPIPPSVGDLPFLETLQFHKLPKLTGPIQPTIAKLTKLKEIYISWTNVSGPVPDFLARLTNLQFLDLSFNNLSGPIPSSLSQLSNLVSLRLDRNRLTGPIPESFGSFKKPGPSLWLSHNQLSGPIPASLANIDPQRIDFSRNKLEGDASVLFGRNKTTQIVDVSRNSLAFDLSKVEFPTSLISLDLNHNQITGSIPVGLTAVDFLQGFNVSYNRLCGEIPQGGRLQKFDVYSYFHNKCLCGSPLTSCK

R-111

>Glyma.06G227200
MGGGFRVLHLVRPFLSFLPEVQTADRKVPFREKVIYTVISLFIFLVCSQLPLYGIHSTTGADPFYWMRVILASNRGTVMELGITPIVTSGLVMQLLAGSKIIEVDNNVREDRALLNGAQKLLGILIAVGEAVAYVLSGMYGSVGQLGVGNAILIILQLCFAGIIVICLDELLQKGYGLGSGISLFIATNICENIIWKAFSPTTINSGRGAEFEGAVIALFHLLITRTDKVRALREAFYRQNLPNVTNLLATILIFLIVIYFQGFRVVLPVRSKNARGQQGSYPIKLFYTSNMPIILQSALVSNLYFISQLLHRKYSGNFIVDLLGKWKESEYGGGQSVPVGGIAYYITAPSSLADMAANPFHALFYLVFMLSACALFSKTWIEVSGSSARDVAKQLKEQQMVMPGHRESNLQKELNRYIPTAAAFGGICIGALTVLADFMGAIGSGTGILLAVTIIYQYFETFEKERASELGFFGF

R-112

>Glyma.12G171400
MARGSSQSQSSTSTATRPGPAGMAPRGSAAATAGMRRRRLGGGNSSASTGVGGGSSNMLRFYTDDAPGLKISPTVVLVMSLCFIGFVTALHVFGKLYRSKSGGAV

R-113

>Glyma.12G168500
MDAIDSVFDPLREFAKDSVRLVKRCHKPDRKEFSKVAVRTAIGFVVMGFVGFFVKLIFIPINNIIVGSG

R-114

>Glyma.15G051600

MERVTSSFMGSFFFWAILVLDLVLKASGNQEGDALNALKSNLQDPNNVLQSWDATLVNPCTWFHVTCNSDNSVTRVDLGNADLSGQLVSQLGQLTNLQYLELYSNKITGKIPDELGNLTNLVSLDLYLNTLNGPIPTTLGKLAKLRFLRLNNNSLTGGIPISLTNVSSLQVLDLSNNHLKGEIPVNGSFSLFTPISYQNNLGLIQPKYTPSPVSPTPPPASSGNSNTGAIAGGVAAGAALLFAAPAIALAYWRRRKPQDHFFDVPAEEDPEVHLGQLKRFSLRELQVATDNFSNKHILGRGGFGKVYKGRLADGSLVAVKRLKEERTQGGELQFQTEVEMISMAVHRNLLRLRGFCMTPTERLLVYPYMANGSVASCLRERQESQPPLGWPERKRIALGSARGLAYLHDHCDPKIIHRDVKAANILLDEEFEAVVGDFGLAKLMDYKDTHVTTAVRGTIGHIAPEYLSTGKSSEKTDVFGYGVMLLELITGQRAFDLARLANDDDVMLLDWVKGLLKDRKLETLVDADLQGSYNDEEVEQLIQVALLCTQGSPMERPKMSEVVRMLEGDGLAEKWEQWQKDETFRQDFNNNIHHPNANWIVDSTSHIQADELSGPR
