# Supplementary material for: Deep learning analysis of soybean cyst nematode effectors to proven soybean resistance genes and homolog identification in the sugar beet-sugar beet root maggot plant pathosystem
Source: Data Brief. 2026 Apr 11;66:112749. doi: 10.1016/j.dib.2026.112749 (PMC13112395; doi:10.1016/j.dib.2026.112749)
Supplement: Supplementary file 3 — Supplemental data 3. The 114 B. vulgaris top matches to the 114 G. max defense protein sequences (Sequences obtained from Supplemental data 2 BLASTp searches). [file mmc3.docx]

R-1 homolog

>Bevul.9G059300.1.p

MGKSYPTVSEDYQKSIEKAKRKLRGLIAEKHCAPIMLRLAWHSAGTFDVQSRTGGPFGTM

RHKGELAHGANNGLDIAVRLLEPIKEQFPELSYADFYQLAGVVAVEVTGGPEVPFHPGRE

DKPEPPQEGRLPDATKGCDHLRDVFIKQMGLTDQDIVALSGGHTLGRCHKDRSGFEGPWT

SNPLVFDNSYFTELLTGEKEGLLQLPSDKALLSDPVFRPLVEKYAADEDAFFADYAEAHL

KLSELGFADA*

R-2 homolog

>Bevul.5G018000.1.p

MAALLVIMLWFIPMAFAGHNYGEALSKGILFYEAQRSGFLPHNQRVNWRSHSGLNDGKIS

GVDLVGGYYDAGDNVKFGLPMAFTVTMMSWSVIEYGRQMASNGELGHALEAIKWGTDYFI

KAHPEPNVLYGEVGDGNTDHYCWQRPEDMTTDRSAYKIDPSRPGSDLAGETAAAMAASSI

VFARSNPAYARELLTHARQLFEFADKYRGKYDSSITVAQKYYRSVSGYADELLWASAWLY

KATNDQYYLSYLARNADALGGTGWAMTEFGWDVKYAGVQVLVSKFLMQGKAGPYRSVFQR

YQQKAEYFLCSCLGKGTKNVQKTPGGLIFRQRWNNLQFVTGASFLLSVYSDYLSSARSSM

QCSAGRVSPTELFSMAKSQIDYILGDNPRATSYMVGYGSNYPQHVHHRASSIVSYKSNPK

FVTCRGGYATWYGMKSSDPNVLTGATVGGPDAYDNFADERDNYEQTEPATYNNAPLIGVL

ARLTGHSGYSQLLPVEIPHVKPTPPPRSKSTPARKARPTPAKKAYRSPAPRAKAVRASAS

SSAHISINQKSTASWVHRGRTYYRYSTTVTNKSNKNLKDMKLSINKLYGPIWGLTKSGNS

YGFPAWINALPSGKSLEFVYVHASTPASISVSSYRLS*

R-3 homolog

>Bevul.7G018600.1.p

MNGASSIAPLAERLKGKVAIITGGASGFGETTARLFVQHGAKVIIADVQDEKGMILCNDI

IASNNTTSHHGNNDVELISYVHCNVSDETDVKNLVDLTISKHGKLDIMFNNAGIPGDIDP

TIVGATIENFKKVFNINVFGCFLGAKHAARVMIPSKSGVILFTSSLASLVAGESPHAYTM

SKHAIIGLMKNLCVEMGQYGIRVNAISPCGVATPLLSNALGMDKGDVDELLCESAVLKGV

APEALDVAHAALFLASDEAKFVSGVNFVIDGGYSVTNPSFSMAVKKLFA*

R-4 homolog

>Bevul.3G031000.1.p

MASDRKVHTFEEVSKHNSNKDCWLIISGKVYDVSPFMDDHPGGDEVLLSATGKDATNDFE

DVGHSDSAREMMDKYYIGEVDVSTVPLKRSYVPPQQAHYNHDKTSEFIIKILQFVVPLLI

LGLAFVVRHYTKKED*

R-5 homolog

>Bevul.6G070900.1.p

MARFTALLNVIIFLSLTLIARSSDDELNCVYTIYVRTGSVLKAGTDSKISLELWGQNGDG

VRITDIENWGGLMGPKYDYFERGNLDVFSGRGPCLNAPVCAMRVVSDGYGPHHGWYCNYV

EITTTGPHIKCAQQKFDVEQWLASDAYPYELTATRNYCPVSLTKELVPTFRSLSSA*

R-6 homolog

>Bevul.7G067700.1.p

MGYWKTKVLPKIQKVFGKDATKKAAAVEACKSFDDCKEEVSKEFEEKKAELQPKVLEVYE

ASTTEIKSLVKEPKEAGIKKHSVKVQSFLDELVKIEFPGSKAVCEASSKFGPALIPGPVI

FVFEKVSTFIVTEEKPREVEAPAAEETAASKETSEVEAEKEIVVEEGATAAAEPTAVDTT

LPKEDPPKVEQPQAEETPAKPEQPTA*

R-7 homolog

>Bevul.8G174200.1.p

MVAKYLVVLLAITLLVWGNYNVSATEFVVGDAAGWNVPDSPTTYSSWASEHNFSIGDILV

FNFTSGSHTVALVNKTAFETCNTDDTISVTSQSPSKVPLQEIGQLHFICTIGAHCSSGQK

LTINVSDHSSSSSSSSSPPLRAPVSAPLSSSSPSNLTPLAIVSSASFVFFLLVDVLV*

R-8 homolog

>Bevul.4G135000.1.p

MANYSLVASAALLLVLLPITAFGNSENCSQVTCGKGTCKSNPDNMPHGFVCECDSGWRRT

RLSDSEEQHLQFLPCIIPNCSINYSCMPAAPPLPPIPTNPHDNSIFNPCNWVYCGEGTCR

NSSDYMHMCQCQPGAYNLFNASHFPCYSDCAFGSDCSKLGINVSKGSVTLGPGSSPSPSS

SQTSRGENHGMRSWKLTWIFITMMSLAATLW*

R-9 homolog

>Bevul.9G062000.1.p

MADQLSDDQISEFKEAFSLFDKDGDGCITTKELGTVMRSLGQNPTEAELQDMINEVDADG

NGTIDFPEFLNLMARKMKDTDSEEELKEAFRVFDKDQNGFISAAELRHVMTNLGEKLTDE

EVDEMIREADVDGDGQINYEEFVKVMMAKKRKMNVEVNRKQIAKQQKPKRRWRNRCKSWL

CSNNE*

R-10 homolog

>Bevul.4G031400.1.p

MEGTVVRRVIPSDNSCLFNAVGYVMDHDKQKASELRQVIAATVCSDPVKYSEAFLGKPNE

AYCSWILDSEKWGGAIELSILADYYGREIAAYDIQTNRCDLYGQDRNFSERVMLLYDGLH

YDALAMSPSEGAPEEFDQTVFSVRNDRTIGIEGLAQNFVKSQHRQRKFTDTGNFTLRCGV

CQIGVKGQKEAMEHAKTTGHVNFQEYK*

R-11 homolog

>Bevul.3G232200.1.p

MMHQISLSHIKDTRVISHPPIFSFKSPPHFIKHLVCIPLSSSTSSSAIATKNPPFSLSSF

PISAMDPVNSWGNSSLEEVDPEIHDLIEKEKRRQCRGIELIASENFTSFAVIQALGSALT

NKYSEGMPGNRYYGGNEHIDVIENLCRSRALECYRLNPEKWGVNVQPYSGSPANFAAYTA

VLNPHDRIMGLDLPSGGHLTHGYYTAGGKKISATSIYFESLPYKVNFQTGYIDYDKLEEK

ALDFRPKMIICGGSAYPRDWDYARFRSIADKCGALLLCDMAHISGLVAAEEAASPFEYCD

IVTTTTHKSLRGPRAGMIFYRKGPKPPKKGQPEDAVYDFEDRINFAVFPSLQGGPHNHQI

GALAVALKQAMSPGFKAYAKQVRANAVAIGNYLMSKGYSIVTGGTENHLVLWDLRPLGLT

GNKVEKLCDLCNITVNKNAVFGDSSALAPGGVRVGAPAMTSRGLLEKDFEQIGEFLHRAV

TITLSIQKEYGKLLKDFNKGLVNNKDIEQLKADVEKFSSSYDMPGFLKSELKYKD*

R-12 homolog

>Bevul.5G033300.1.p

MSHLKPLSILFCLLLVITSLVFTPSNAITFTVKNNCPYTVWGAAVPGGGQQMNSGSTWTV

TANPGQQGARIWARTGCTVTGPNGLRCNTGDCGGLLQCTAYGTPPNTLAEYGLKQFNNLD

FFDISLVDGFNVPMSFLPTGGCSRGITCSSDLNGPCPGPLRVNGGCNNPCTVFKTDNYCC

NSGSCGPTDYSRYFKGKCPDAYSYPKDDATSTFTCPTGTNYMVTFCP*

R-13 homolog

>Bevul.1G041500.1.p

MATQALVSSSLTSSVETARQILGARSGVSPSSKKNSFVVRASATPPVKQGANRPLWFASK

QSLSYLDGSLPGDFGFDPLGLSDPEGTGGFIEPRWLAYGEIINGRFAMLGAAGAIAPEIL

GKAGLIPAETALPWFQTGVIPPAGTYNYWADPYTLFVFEMALMGFAEHRRLQDWYNPGSM

GKQYFLGLEKGFAGSGDPAYPGGPIFNPLGFGKDEKSMKELKLKEVKNGRLAMLAILGYF

IQGLVTGVGPYQNLLDHLADPVNNNVLTSLKFH*

R-14 homolog

>Bevul.2G099800.1.p

MAGKGEGPAIGIDLGTTYSCVGVWQHDRVEIIANDQGNRTTPSYVAFTDSERLIGDAAKN

QVAMNPINTVFDAKRLIGRRFSDASVQADIKLWPFKVIPGPGEKPMIVVNYKGEEKQFAA

EEISSMVLTKMKEIAEAYLGSTVKNAVVTVPAYFNDSQRQATKDAGVISGLNVMRIINEP

TAAAIAYGLDKKATSVGEKNVLIFDLGGGTFDVSLLTIEEGIFEVKATAGDTHLGGEDFD

NRMVNHFVQEFKRKHKKDISGNPRALRRLRTSCERAKRTLSSTAQTTIEIDSLFEGVDFY

SPITRARFEELNMDLFRKCMEPVEKCLRDAKMDKSTVHEVVLVGGSTRIPKVQQLLQDFF

NGKELCKSINPDEAVAYGAAVQAAILSGEGNEKVQDLLLLDVTPLSLGLETAGGVMTVLI

PRNTTIPTKKEQVFSTYSDNQPGVLIQVYEGERTRTRDNNLLGKFELSGIPPAPRGVPQI

NVCFDIDANGILNVSAEDKTTGQKNKITITNDKGRLSKDEIEKMVQEAEKYKSEDEEHKK

KVEAKNALENYAYNMRNTVKDEKIGAKLSEDDKKKIEDAIDSTIQWLDGNQLAEADEFED

KMKELESICNPIIAKMYQGAGGDMGGGMDDEVPPAAGGPGPKIEEVD*

R-15 homolog

>Bevul.1G104100.1.p

MASFSSSTSTTQKLSIFLTIFSLLHLISAARNTPLEDPATTSLLQYHQGPLLSGKITVNL

IWYGKFTPSQKAVISDFVTSLSPNTPTTKSHIHHPSVSTWWNSIGRYYHIANKKASTLSL

SLGKQISDESYSLGKSLTSKHLVELASKGAKSNAVNVVLTASDVAVDGFCSSRCGTHGSN

SRMGRDKFAYIWVGNSETQCPGQCAWPFHQPIYGPQNSPLVAPNNDVGLDGMIINLASLM

AATVTNPFGNGFYQGPKEAPLEVASACTGVYAKGAYPGYAGNLLVDATSGASYNANGVNG

RKYLLPALYDPSSSTCSTLV*

R-16 homolog

>Bevul.4G051500.1.p

MMIEVLIYFSSFGQMIITAIDALNEKMGSNKTSISKFIESKYQNLPAGHSTLLRHHLNRM

KDTGELIFWKNNYMKPDPNFIRRGRGRPPKPKEPGMPTSGGSARPRGRPRKDEFGADEVQ

VPIQVPGAAPPKKQKMSLFEDFDANIGMGSNTKTGRPRGRPPKVKPQMGGEGVSA*

R-17 homolog

>Bevul.8G074100.1.p

MVEIARANGNHEVTINVKDGDQSLPQTSSNNTSSSNSMENPLSCLSVPFLQKMIAEVLGT

YFLIFAGCAAVTVNLDGDKVITLPGIAIVWGLAVMVMVYSVGHISGAHFNPAVTIAFAVC

RRFPWKQVPAYVVCQVVGSTLASGTLRLIFNGHQTQFAGTSPAGSSLQSLVIEFIITFYL

MFVISGVATDTRAIGELAGLAVGSTVLLNVMFAGPISGASMNPARSLGPAIVSHHYEGIW

IYLVGPTVGAVAGAMVYNLIRFTDKPLREITKTGSFLKSKGSSVSRNGSSYR*

R-18 homolog

>Bevul.2G121900.1.p

METHSKNMSKNNLIRNLIVFFALSITMLVTSSSTTPKIFHYAIFLPIIFYLLYSLFSPHA

QNSQNTPPGPFSLPIFGNWLQVGNDLNHRRLAAFSKTYGPVFFLKLGVRNLVVISNPKLA

CEVLHAQGVEFGSRPRNVVFDIFTGGGQDMVFTAYGDHWRKMRRIMTLPFFTNKVVNTYS

PMWEEEMDKVVSDLKHNDHIIGIKAKKEGFVLRKRLQLMLYNIMYRMMFDEGFESMEDPT

FIEATKFNSERSRLAQSFEYNYGDFIPLLRPFLRSYLSKCRDLQKRRLAFFNNYYVEKRR

NIMATNGENHKISCAIDYIIDAQKKGEINEDNVLYIVENINVAAIETTLWSMEWAIAELV

NHPKIQQKIRQEIATNLNGNPVTESNLEQLPYLQATIKETLRLHTPIPLLVPHMNIEEAK

LGGYTIPRESKVVVNAWWLANNPEWWHDPQEFRPERFLQEEVTTDVTVGGGKVDFRFLPF

GVGRRSCPGIILALPILGLIIGKLVTNFEMKPPLGEEKIDVSEKGGQFSLHIAKHSTVVL

SPVHAA*

R-19 homolog

>Bevul.4G043100.1.p

MHVPQKPVLRPTGSDTRGNTSSLHWFLILHLNINNHSLFSLFSFKMQIFVKTLTGKTITL

EVESSDTIDNVKAKIQDKEGIPPDQQRLIFAGKQLEDGRTLADYNIQKESTLHLVLRLRG

GMQIFVKTLTGKTITLEVESSDTIDNVKAKIQDKEGIPPDQQRLIFAGKQLEDGRTLADY

NIQKESTLHLVLRLRGGMQIFVKTLTGKTITLEVESSDTIDNVKAKIQDKEGIPPDQQRL

IFAGKQLEDGRTLADYNIQKESTLHLVLRLRGGMQIFVKTLTGKTITLEVESSDTIDNVK

AKIQDKEGIPPDQQRLIFAGKQLEDGRTLADYNIQKESTLHLVLRLRGGMQIFVKTLTGK

TITLEVESSDTIDNVKAKIQDKEGIPPDQQRLIFAGKQLEDGRTLADYNIQKESTLHLVL

RLRGGF*

R-20 homolog

>Bevul.8G010700.1.p

MPAGGFVTQGGKEYPGKLTPYVLATCIVAAMGGLIFGYDIGISGGVTSMPDFLKKFFPSV

YRKEALDKSVNQYCKFDSVTLTLFTSSLYVAALVASLVASVVTRKLGRKLSMLFGGLLFC

VGAIINALAKDVAMLIVGRILLGFGVGFANQSVPLYLSEMAPYKYRGSLNIGFQLSITIG

ILIANVLNYFFAKIHDWGWRLSLGGAMVPAIIISIGSLLLPDTPNSMIERGKRDEALLKL

KRVRGVDDVEDEFNDLVVASENSKKVEHPWRNLLQRKYRPHLTMAIAIPFFQQLTGINVI

MFYAPVLFKTIGFGDNASLMSAVITGGVNVLATMVSIYGVDKWGRRFLFLEGGVQMFICQ

VVVAIFIGVKFGVSGTAENLPEWYAIVVVLFICIYVSAFAWSWGPLGWLVPSEIFPLEIR

SAAQSINVSVNMFFTFVIAQIFLTMLCHLKFGLFLFFAFFVVVMSIFVYLFLPETKGVPI

EEMSIVWKNHWFWGKYIPDEDLPHGASTLEMGKNNNNVMPSKSVY*

R-21 homolog

>Bevul.8G068200.1.p

MESQVEGNRSRVTTVDSLDQWEFLVNKAKNLACPIVVHFGASWCIPSVVMNPLFQELAFT

YSDVMFLTVDVDDVKKVASKMEVTAMPTFLLMKDGIPVDKLVGANPDEIRRRVESFIQSN

NGAYMA*

R-22 homolog

>Bevul.6G185100.1.p

MDTETDATSSHMIPDTETSSYLPPSMLYKRVNWDVFLSFRGEDTRHIFTTPLRDTLKTKG

LRPFMDDEGMERGDTIQPSLDEAIVDSALAVAIISPRYADSRWCLDELARIFECRKRVIP

VFYKVHPSDVRKQRGVFGMGFQEMLGDSNRFSEAQVKKWRWAMENIGNISAIHGYASNMA

QFIYNDSDEAKEVIRRIVTQVWNELKNTPEYVVKLAVGLDSRVDKVVGKLNLQAQGARFL

GIHGTPGIGKTTLAKAVYNKLAVKFKNRCFISNVREKIATEEGFLTVQEELLKDLSLGKV

QLVVKQVDEGKKAIKRFVEETRVLVVIDDVFEAGQLENLGIVREWFKEGSMIIVTSRNVD

ALLHSACNDELYEAQKLDESEALELFSIHALGRREPTNEFRDVSKKIVSLTGGLPLALEV

FGSSLVKKTIKVWEDAISKFGVTRPPSIQDVLKFSYDSLDHQEKIVFLDISCLLLQMNMD

REDVVDILAGCDLGADLAIDNLVTKSLMKIIEGKLWMHDQIRDMGRQIVTEKVDDPEKLR

RLWDAKDIMRVFRNKKAREDVRGMILDIKKPQDFTDEMIYWYHFRRHPSMTTFIRYLAKM

FKGLLTSWHKTEKDDNSILHARYFENMVQLKLLQMNYAKLQGDFRCMPSELRWLQWKGCP

QKIRPFNIPEELRVLDLSRSNIDRLWSSDYSCWGFNKVVGNLVVVDLSYCPRLTALPDMS

GHKHLKKLTLEYCVGLTKIHPSIGNMSSLVRLNLQGCSNLLDFPRDVSGMKSLKELILSD

CSKFRELPQDVRSMTSLNQLFLDRTAITKLPEYLFSLTQLEMLSLNGCPLLTSLPECIGS

LLSLKELILSATALDNIPSSVGSLTHLEKLNLMHCHKLIALPDSIGNLKSLTELYLNTSS

IKQFPASICSLKSLRVLLLGGCRSLLNMPVSVGGLASIIELELNNTDIKILPDEICSLKL

LEKLRMSNCTLLEKLPDLVGKISNLIILSVEGAAITQLPKSIVELENLTWLNLNKCRKLS

SLPNDIGRLKSLRILMMEDTAVTCLPESFGSLDELRTLKLKKSSNDLPKLSALPNSFCKL

SNLEEFSARACGVSGQIRDDFVELSKMETLDLGYNSFHKLPSTLEGLSVLTQLKLRHCEK

LRFLPQLPSTLVELDAADCYSLESIYNLSKLERLEELELANCNQIKDVPGLECLKSLRRL

FMGGCNARPSKVRRRLSKVALRNLNNLSIPGSEIPGWFSQKAVNFTAHPNLAIKSVLVSV

VVSVDIQVLDDMRAQLPSIVDIQASIVRLDESIFYTTLILMGIPCTREEQFYLCRFKEYH

PLSSLLRDGDIIQVGLRYGTIPGIELLKWGIHLVFENDDDYNGDEDSLQLTESQQSVSQR

LVRFFQSQD*

R-23 homolog

>Bevul.2G239900.1.p

MASIGITSYRDRTAEFRSFSETLKKIGGISAVDSAGNHPSTSIYSPEKTAASLYRSEFNT

KASRIGLGIHEANRKIARLAQLEKKSSMFNDPTVEIQELTALIKSDITALNVAVVDLQNL

QSVETSSSYSDDKAVHANAICDDIKNRLMAATKQFQEVLTARTQNIRAHETRKQLFSANA

SRDNPFQHPTKPITEPPPWSNSSNVSASMEPTVTPSSGIQAGSQLRRRMGTETIPSNNME

MSMLQQAAPEQVNYSQNRASALQNVESTISELSGIFTHLATMVAQQGELAIRIDDNMEES

RTNVEGAHNALLKHLTRISSNRSLLIKLFAVLILFLVVFVFFIV*

R-24 homolog

>Bevul.5G203500.1.p

MAAEKIGLREEIIGKACTVAIKAHKSSSNKPYICEKSKSSSSEVIFGFTGTWDFGDWFTH

KPFGTTKINLNLFPSLKTIGSNEAATVNQAFLQRFEAILAKTSFKNEVEKAATEKPIVFA

GHSAGAATAIFATLWFLDSQNQRHINQIPPRCITFGSPLVGDNILSHALKREHWSQYFTH

FIMRYDIVPRISLTPLSAIEREFQPVLHYYNPKSPFFASDSVGRTTEASMFILTVIRNAS

LVTSRVARKFMGCTNLLLENATNFIELSPYRPFGTYVFCTGNGKFVTIKNPDAIFQILYY

CLQLGSESEWDEVVQRSLKEHVIYEHELLENLEMPSVADLEHLVDLPLSAENMGNSENFA

ICSALDDLGLSARARLCLRAAGQLEKQKARNQIKINSNKNKIRDVLNMLERYRELCEVRK

VGYYDAFKMQNHTEDFNNNIRRLELAGIWDEIIEMLKRNELPDEFEGQKEWIEIGTRYRR

LVEPLDIANYYRHLKNEDTGSYMGKGRPSRYRYVQQWREHVEKMEAETVPDSCFWAEVEE

LCISTRNIEFPEDIKKRILHLERNVSIWAQNELLGKDVFLNESTFVQWWKKLPIQHRSVS

CIRDLFHE*

R-25 homolog

>Bevul.8G131300.1.p

MVIDSRTAFSDSNDISNGSSICCVAATTTTTTTAAENSLSFTPDAAALLRLSENLDSLFQ

PSLSLSDSDSFADAKIVVSGDSREVAVHRCVLSSRSSFFRSAFASKREKEKERDKERVVK

LELKDLAGDFEVGFDSVVAVLGYLYSGKVRNLPRGICVCVDEDCSHEACRPAVDFVVEVL

YLSHKFEIVELVSLYQRHLLDILDKIAPDDVLVVLSVAEMCGNACDGLLARCIDKIVRSD

IDVTTIDKSLPQNVVKQIIDTRKELGFTEPGRVEFPDKHVKRIHRALESDDVELVRMLLK

ERHTTLDDAYALHYAVAHCDAKTTTELLELGLADVNLRNLRGHTVLHVAAMRKEPKIIVS

LLTKGAHPSDITSDDKKALQIAKRLTKAVDFYKTTEQGKDAPKDRLCIEILEQAERREPL

LGEGSVSLAKAGDDLRMKLLYLENRVALARLLFPMEAKVAMDIAQVDGTSEFTLSKNIAD

ARRNAVDLNEAPFILKEEHLQRMKALSKTVELGKRFFPRCSDVLNKIMDAEDLSQLAFLG

KDTPEERQRKRKRYLELQDALTKAFTEDKEEFDRSTLSSSSSSTPMGRPYGKTNFKR*

R-26 homolog

>Bevul.7G205400.1.p

MGICLSARIKAESPSHTGINSKYVSKDGNDPTFPSSKVSSAPLTPRSEGEILQSSNLKNF

SFSELKAATRNFRPDSVLGEGGFGSVFKGWIDENSYAATKPGTGMVIAVKRLNQESYQGH

REWLAEVNYLGQLYHPHLVKLVGYCVEDEHRLLVYEFMPRGSLENHLFRRGSYFQPLSWH

LRMKVAYGAAKGLAFLHSAETKVIYRDFKTSNVLLDSNYTAKLSDFGLAKDGPTGDKSHV

STRVMGTYGYAAPEYLATGHLTARSDVYSFGVVLLEMLSGRRAMDKNRPSGEHNLVEWAR

PYLTNKRKIFRILDNRLEGQYSLDAAFKVASLAFRCLSSEPKFRPLMNEVVKELEQLQDS

SKEARSSSSTGGRTRRRSAQESTNRSISAVSYPSI*

R-27 homolog

>Bevul.2G055200.1.p

MAGTKTIVFLLFASFIAIASAGNFYKDFDITWGDGRGKIINNGQLLTLSLDKTSGSGFQS

KNEYLFGKIDMQLKLVRGNSAGTVTAYYLSSQGASHDEIDFEFLGNVSGQPYTLHTNVFS

QGKGGREQQFRLWFDPTTDFHTYSILWNPQRIIFSVDGTPIREFKNSEYMGIPFPKNQPM

RIYSSLWNADDWATQGGRVKTDWSKAPFTASYRNFNANACVWSKGKSSCKSSSVASKINP

WFTQEMDSTSQARMKWVQKNYMIYNYCTDRQRFPQGLPKECRVN*

R-28 homolog

>Bevul.5G087100.1.p

MPVPLAPYPTPPVPFTPPANGAQSQLVCSGCRNLLLYPAGATSVCCAVCNAVTAVPPPGT

EMAQLVCAGCHTLLMYIRGATSVQCSCCHTVNLALEANQVAHVNCGNCRMLLMYQYGARS

VKCAVCNFVTSIGASASTPEHKFNNS*

R-29 homolog

>Bevul.3G026900.1.p

MNNLISSSFTRFRENSGDIEMGGPPTPSPNPGVNLDKFFDDVETIKDDLKEIEDQQKKLR

AAHEESKTAHTAASVKELRARMDRDVGLALKKAKMIKVRLEALDRSNAANRNLPGCGPGS

SADRTRTSVVNGLRKKLKEKMDEFQELREKINGEYRETVERRFFTVTGENPDERTVDLLI

STGESESFLQKAIQQQGRGQVLDTIAEIQERHDAVKDIERNLVELHQTFLDMAVSVQFQG

EQLDDIQANVGRASSFVRGGTEQLYVARKHQKNTRKWTCFAIILLLVMILVVVLSLQPWK

K*

R-30 homolog

>Bevul.7G120300.1.p

MGQQSLIYSFVARGTVILAEFTEFSGNFTSVATQCLQKLPSSSSKFTYTCDGHTFNYLVD

NGFTYCVVAVESAGRQIPIAFLERVKDEFTKKYGGGKAATASAKGLNKEFGPKLKEQMQY

CVDHPEEINKLAKVKAQVSEVKGVMMENIEKVLDRGEKIELLVDKTENLRSQAQDFRTQG

TKIRRKMWWENMKIKLVVIAIIVALIIILILSICGGFKCTH*

R-31 homolog

>Bevul.5G043900.1.p

MSMDSDTSSHAGDYKYFRQMTRDRLLHEMLGSTKKGDSRSNWKVLIMDRFTVKIMSYACK

MADITGAGVSLVEDIYKRRQPLPTMDAIYYIQPSKENVVMFLSDMAGKKPLYKKAFVFFS

SPVARELVSHIKKDSTVLPRIGALSEMNLEYFAIDRQGFVTDNEMSLDELFGDDENHRKA

DACLNMMAARAATAFASMRELPFVRYRAAKSLDANTMTTYRDLVPTKLAAGIWNYLTKYK

TSIANFPQTETCELLVLDRSVDQIAPVIHEWTYDAMCRDLLNLEGNKYIYEVPSNTGGPP

EKKEVLLEEHDPIWFELRHLHIADASERLHEKMTNFVTKNKAAQIHHSSRESGELSTREL

QKMVQALPQYSEQIEKLSLHVEIAGKINRIIRDQKLKEIGQLEQDLVFGDAGTKEVINFF

RLHEDVSRENKLRVLMVYAAVYPDKFEGDKGSKLMQLAGLSSDDTNAVKNMKLLGTPPET

KKSSLGAFALKFDNKKKHGVRKERVGEETAWQLSRFYPMIEELVEKLCKGELPKNEYPCM

NDPSPTFHGTSHPAAIQYTETPVAHSMRSRRTPTWASRPRNSDDGYSSDSILRHASSDFK

KMGKRIFVFIVGGATRSEMRTCYKLSTKLNREIILGSSSIDDPPQFITKLKLLSASELSL

DDLEI*

R-32 homolog

>Bevul.5G180800.1.p

MIGIKNSPLHRFAKPSSARSKVPSNASSNPFDSDDESDRKPKQGSATKPPSAPPKDLPNS

SMTNPFDDDVDNREREESSSTSKYSFTYAQRNKYKNDFYDAGGVDNQSVEDLEHYAAYKA

EDTTKSVDNCLRIAEGIRDDATKTLVTLHQQGEQIRRTHEEAASMDYDLSRGEKLLGSLG

GLFSKTWKPKKTAPVKGPIMLRDDPVWSKGSHLEQKERLGLTSTKNGRSKSRTPPPEPTN

ALQKVEAEKVKQDDALSDLSDLLGELKVMAQDMGSEIQRQNHSLNHVDDDVAILNDRVKD

SNQRARRLLGK*

R-33 homolog

>Bevul.8G201400.1.p

MVKVTMIARVMDGLPLAEGLDDGRDVQDVEYYKQQIKALFKNLSKGQNDPSRMSVESGPY

IFHYIIEGSVCYLTMCDRSYPKKLAFQYLEDLKNEFERVNGNQIETAARPYAFIKFDTFI

QRTKKLYQDTRTQRNIAKLNDELYEVHQIMTRNVQEVLGVGEKLDQVSQMSSRLTSESRI

YADKARDLNRQALIKKWAPVAIVIGIVVLLFWVKKKIW*

R-34 homolog

>Bevul.9G137300.1.p

MGLWSPILRIFSVIFGFVGFGLGITIGLTVGYYLFIYFQPTDVKDPDIRPLAEQDTAKLQ

RLLPEIPLWVKSPDYDRMDWLNKFIAQMWPYLDKAICKTAKNIATPIIAEQIPKYKIDSV

EFEKLTLGSLPPTFQGMKVYVTDEKELILEPSLKWAGNPNILVALKAFGLKATAQVLDLQ

VFASPRITLKPLVPSFPCFANIYVSLMEKPHVDFGLKVLGADLMSIPGLYRFVQELIKDQ

VANMYLWPRTLDVPILDPAKAMRKPVGILHIKVIRAMKLKKKDLLGGADPFVKLKLTDDK

TPSKKTTVKHKNLNPEWNEEFKLTVRDPETQSLQLNMYDWEQVGKHEKMGLNVVPLKDLT

PDEPKEFTLDLLKTTDPNDPQNEKSRGQIVIELTYKPLKEEDVGKDFDDSMQQAPAGTPA

GGGVLIVRIHEGQDLEGKHHTNPYARVIFRGEEKKTKTIKKNRDPRWDEQFEFVLEEPPT

KDKMHIEVYSTSSRSLLHQKESLGYIDISLGDVVNNKRINEKYNLIDSKNGLILVELIWR

AAPPKS*

R-35 homolog

>Bevul.4G061800.1.p

MANDTMIVTNTPARDLVSTNFVYCSLVDLRRLAAPGSRLVFAYVGDSWVLSVGAHDDVPN

GSIGLNAVQRKYLRVSTGDSIPVRRFVPPENFKLAVLTLELDYLSRAKARDEQVDAVIVA

QLIKRRLVDQVLTVGQKVTFEYLGNNFLFTVNQAVIEGQEKSDSERGLLSADTYVIFEAA

GGSGIKIINQRESASSNLFRQKEFNLKALGIGGLGDEFADIFRRAFASRVFPPHVTSKLG

IKHVKGMLLYGPPGTGKTLMARQIGKMLNGKDPKIVNGPEVLSKFVGETEKNIRDLFADA

EHDQRTRGDQSDLHVIIFDEVDAICKSRGSTRDGTGVHDSIVNQLLTKIDGVEALNNVLL

IGMTNRKDLLDEALLRPGRLEVQVEISLPDEAGRLQILEIHTTKMKENSFLAPDINLAEL

AARTKNYSGAELEGVVKSAVSYALNRQLNMDDLNKPVDEENIKVTMNDFLEAIDEVKPAF

GASTSDLERCRLNGMVDCGVRHEHIQQRTMLLAEQVKHSKGSPLLTCLLEGPSGSGKTAM

AATVGICSDFPYVKIVSAESMIGLSESTKCAHIVKVFEDAYRSPLSVIILDDIERLVEYV

AIGPRFSNIISQTLMVLLKRLPPLGKKLLVLGTTSEVGFLDSIGIRDTFSVTYNVPTLKT

DDARKVLQQLGVFSEYDIDSAAEALNDMPIKKVYMLIEMAAQGDQGGKAEAIYSGKEKID

ISHFYDCLQDLVRY*

R-36 homolog

>Bevul.2G187100.1.p

MGDHLARGEDCEKKAEKKLSGWGLFGNKHEDAAELFEKAANSYKLAKSWDQAGAAYLKLA

NCYLKVESRHEAANAYAEAGHAYKKTSSKEAVSCLEQAVNSFLEIGRLNMAARYCKEIGD

IFETEENLEQAIVWYDKAADLFQSEEVTTSANQNRQKVAQYSAQLEQYPKAIEIYEDIAR

QSINNSLLKYGVKGHLLNAGLCHLCKGDVVSINNALDKYQDLDPTFVGTREYKFLNGLAS

AIDEQDVAKFTDLVKEFDSMTPLDAWKTTLLLRVKEKLKAQDEDDDDLT*

R-37 homolog

>Bevul.1G192600.1.p

MDFGTLAIYLLCALFATKILYQWLKSYLYTTYKLPPGPPRWPLFGNLLQLGPLPHRDFAS

FCEKYGPLVYIRLGNVDAITTNDPEIIREILVRQDDVFASRPHTLAATHLAYNSGDVALA

PMGPKWKRMRRICMEHLLTTRRLELFVSHRADEARHLVQDVLTRSHKDKVVNLREVLGAF

SMNNVTRMLLGKQYFGAGTAGPQEALEFMHITHELFWLLGLIYLGDYLPFWRWVDPYGCE

KKMREVEKRVDDFHRKIIEEHRKEKKRKEEMGVNEGEMDFVDILLALPGENGNEHMDDAD

IKALIQDMIAAATDTSAVTNEWAMAEVIKHPRVLHKIQQELNTIVGPNRMVTESDLPHLN

YLRCVVRETFRMHPAGPFLIPHESLRHTTINGYDIPSGTRVFINTHGLGRNLKVWDNIED

FYPERHWPLDGSRVEISHGSDFKILPFSAGKRRCPGAPLGVVFVLMGLATLFHAFDWLPP

DGMKAEEIDTKEVYGMTMPKAQPLMALAKPRLAPHLYL*

R-38 homolog

>Bevul.4G172800.1.p

MAQFFGPDETKALQPDLVETGNSCRLSLQSQNSSFRRNSSLGSSKGSLDEEFIREWAKIE

RLPTYQRSRVFLFDDYDGESTTNVNGKRMIDVSMLGPLDRRLFIEKLIKQIEQDNLQLLK

KLRKRMDKVGLHFPAVKVKYKNLFVDMRCEVVDGKPLPTLYNSLKSSLSVFTNLLGSDQQ

EAKLDIIKDVSGVIKPGRMTLLLGPPGCGKTSLLLALSGNLDSSLEIKGDVEYNGHKLEQ

FVPQKTSAYISQHDLHIPEMTVRETIDFSARCQGVGSRADLLIEVCRREKQEGIIPDPDI

DAYMKAISVEGLKSSLQTDYILKILGLDVCADTLVGDAMRRGISGGQKKRLTTGEMIVGP

TKVLFMDEITNGLDSSTAFQIVSYIQQLVHITDASALIALLQTAPETFDLFDDVILMAEG

KIVYHGPRDHVVQFFETCGFRCPKRKGIADFLQEVISQKDQSQYWFLEKPYGYMSVDMFC

EKFRVSHVGKTLYEEVSQPPVKMQDDENAISFSKFSLPKGELFKACLSRECLLMKRNSFI

YIFKIIQLVIIAIITMTVFLRTRMHVDILHANYYLGSLFYGLVMLLVDGFPEVSMTIMRL

PVFYKQRDLYFYPAWAYAIPATLLKVPLSILLAVLWTSLTYYTIGYAPEVGRFFCQVLLF

SGVHLASVTMFRFLASLFQTIVVSNLASVYFLLFAFIFSGFIIPKSSMPVWLEWGFWVVP

LTYGETGLATNEFLAPRWQKMLSSNTTIGQEVLQSRGLLFDGYFFWVSVGALFGFSLLCT

VGYTLALTFLKAPVASAAFISREKLSQLQELRHPCRVDDLESCGDKSTVESQKGNMILPF

QPLTVAFHNVQYYIDTPVEMREQGFTENKLNLLHDITGSFKPGVLTALMGVSGAGKTTLL

DVLAGRKTSGTIEGDIRIGGYPKVQETFARVSGYCEQTDIHSPHITVEESLIFSAWLRLP

DNIDSNTKTDFVNEVLKTIELNEVKDTLVGLPGVNGLSTEQRKRLTIAVELVANPSIIFM

DEPTTGLDARAAAIVIRAVKNIAVTGRTVVCTIHQPSMDIFEAFDELVLLKTGGRIIYSG

PLGQQSSKVIEYFQSIPGMPKIRDNYNPATWILEVTSASAEIELGIDFAEVYNSSVLCQK

NEEFVQQLSIPSVGSRDLHFRSRFSLSGWGQYKSCLWKQQLSYWRSPSYNLTRLMFAFVS

SLIFGLLFWQQGRNIKDQQSIFTVFGSMFAAVMFLGINNCSPVLPHIATEKTVVYRERFA

GMYSSWAYSFAQVTVEIPYLLTVALIFTIITYPMIGYYSSAYKVFWYFYAMSCTLMYFNY

MGMMLISLTPNSQLAAITASAIYALMNLFSGFLIPQPQIPKWWLWLYYLCPSSWTLNGLL

SSQYGDIHEYITVFDKTKTIASFVEDYFGFHHDRLGLVAAVLIIFPLMFAYLFAYFIGKL

NFQRR*

R-39 homolog

>Bevul.6G082000.1.p

MLMIRRNRLSICLFIVALTTIIKHAYCDCSSTNVTRKSFPKSFVFGTASSAYQYEGAVKE

DGRGPCVWDTFAHRLGKVIDFSNADVANDHYHRYLEDVKLMKDLGVSAYRFSISWTRIFP

NGTGKINQAGVDHYNNLINALLANGIQPYVTMFHWDTPQALEDAYKSWLSHQIVNDFGAY

AETLYQKFGDRVKHWITLNEPHTVAAQGYDIGIFAPGRCSILLHLFCRAGNSATEPYIVA

HHLLLAHATAVNIYRTKYQRKQRGTIGAAFDVMWYEPATNTTEDIEAAQRAQDFQLGWFL

DPLMFGDYPSSMRERVGDRLPKFSAAESSLLKGSLDFVGINHYTTYYARNNKTNIIGFLL

NDALADSGTITLPFGHDGKPIGERANAIWLYIVPRGMRELMNYIKHKYGNPIVFITENGM

DDGNSIFKSLEDALKDEKRIRYFNGYLENLAAAIKEDGCNVKGYFAWSLLDNWEWAAGYT

SRFGLYYVDYKNNQKRYPKNSVQWFKNFFNPTC*

R-40 homolog

>Bevul.6G018700.1.p

MFLWKSSQNRDENDPLNRDAKIKELRVALGALSGRHSKFCTDACLRRYLEARNWNVDKAK

KMLEETMKWRDMYKPEEIRWHEVAHEGETGKVSRANFHDREGRAVLIMRPGKQNTTSGEG

NIRHLVYLLENAILNLPEGQEQMVWLIDYTGFSMSTNVSVRTTREIINILQNHYPERLGI

AVLYNPPRIFQAFWRAVKYFLDPKTFQKVKFVYPKNKDSTELMRSYFDVENLPKEFGGNA

TLEYDHEEFSRLMAEDDVKASRYWGFDDKPCHAAQVCAGRDIPVQVQPILAIGHSKAEVA

PEPF*

R-41 homolog

>Bevul.7G211700.1.p

MAAPPARARADYDYLIKLLLIGDSGVGKSCLLLRFSDGSFTTSFITTIGIDFKIRTIELD

GKRIKLQIWDTAGQERFRTITTAYYRGAMGILLVYDVTDESSFNNIRNWIRNIEQHASDN

VNKILVGNKADMDESKRAVPTAKGQALADEYGIKFFETSAKTNLNVEEVFFSIARDIKQR

LADSDTRQEVAQPSITIKPADQSGNQAAAKSACCGS*

R-42 homolog

>Bevul.7G015900.1.p

MASPDMAGAPPPQSVDPEGIDGVRMTWNAWPRTKVEASKCVIPIAASISPIRANPEIPTL

PYAPLRCKTCVAALNPYCRVDFTAKIWICPFCFQRNHFPPHYSGISENNVPAELYPQYTT

LAYSLSSNPGDPHSNSGLIPPVFLFVLDTCMIEEELGFVKSSLRRAIVSLPDNALVGFIS

YGTQVHVHELGFSDMNKVYIFRGSKEITKDHVLDQLGLGPSSRRGGPGHVGAGPGYPKVG

QQQVVQNGLPGSGVDRFLLPASDCEYTLNALLDELQTDQWPVPPGSRALRCTGVALSVAA

GLLGACSPGTGARIIALVGGPCTEGPGTIVSKDLSDPVRSHKDLDKDAAPYFQKAVKFYD

TLGKQLVSQGHVLDLFASALDQVGVAEMKVAVEKTGGLVVLSESFGHSVFKDSFKRLFEE

GEQSLGLCFNGTLELYCSKDIKIQGAIGPCTSLEKKGVAVADTVIGQGNTTAWKLCSLDK

NTNVTVFFDISSSERANPPGTLNPQLYLQFLTSYQSSDGQTMLRITTVSRRWVESVVGSE

ELVQGFDQEAAAVVMARLCSHKMEMEEGFDATRWLDRNLIRLCSKFGDYRKDDSTSFTLN

PCFSLFPQFMFNLRRSQFVQVFNNSPDETAYFRMLLNRENVTNAAVMIQPSLIAYSFNAP

PSPALLDVASIAADRILLLDAYFSVVIFHGMTVAQWRNMGYQNQPEHQAFKELLEAPQQD

SQMIIHDRFPVPRLVVCDQHGSQARFLLAKLNPSATYNNVNELAAVSDVIFTDDVSLQVF

FEHLQRLAVQS*

R-43 homolog

>Bevul.6G123100.1.p

MMLQYHGFSGLKTKRIVFENVFSVLDSVSLEHMKELTARRRLIEESINETSCITEATARE

MSGGLTSRSQQDIQKLEQYLPILENFVIQVDSISRNKNLLISELRIQWSSTVSSSRFRIG

GPKFFQINSLKYELGMILFLYGVTIREQAFEILSEDKQKSACLLRKAAGIYKHLNDEVLP

LLQSILPPEKPPETATNVSFAMNNICLAEAQAIAIKMGEMQGLSAGLLAKLHYGVTQLLD

EASSLLQPGPRDISRRFLDFIVSCSTLHELSSYRHLAESYKHAEQVGVAIGLLNRAMSKA

KNLPGEQSWREVFKNELNKISDMLKKYNDENAFVWHHKIPLDFELPSLEGRTIVTATPYE

PERWERQITFST*

R-44 homolog

>Bevul.9G140300.1.p

MLVSNSFDLWQTDSFFSAAEEVQESADILESAYRTWIKQKREGLASNDLDELRREVQTAL

GTAKWQLEEFERAVSLSYGNCRDQNKMARHQQFVVAIESQISRVEDTLRETLNEEGKQPF

RWVNLNEDERDDLAMFLSGSSGTFTKNASDKIVKPSTGLVEKNLYKKKDLDYAACSSSST

TLRDDVVINIGQPNSLLELSAREVLGTKGDMNCEAERTRSAKRTRVSPNSGALKIVIPNA

ENEMRALGSTIEATPKEKGSKTSSLLQFRGINCINKLFKCSNGLNSGMQSSKRLQFKSSM

RLIIILMVSFFLLVPYFLQSS*

R-45 homolog

>Bevul.5G117200.1.p

MNNLLSDSFEIPRGESSRGGDLEMGVNLQNSGELGLQNFSKKVQEVDKQYDKINELLRKL

QDAHEESKAVTKASAMKAIKKRMEKDVDEVQKIARLIKSKIEELDRDNLSSMQKPGCGKG

TAIERARTAQTIALKKRLRDKMAEFQVLRENIHQEYREVVERRVYTVTGQRADEEKIDQL

IETGDSEQIFQKAIQEQGRGQVMDTLAEIQERHDAVRDVERKLLELQQIFLDMAVLVDAQ

GDLLDNIESQVSNAVDHVNTGNTALQKAKSLQKNSRKWMCIAIIILLIIVAVIVVGVLKP

WQQKGGA*

R-46 homolog

>Bevul.7G059200.1.p

MSVIDILTRVDAICHKYDKYDVDKQRESNVAGDDAFARLYSSVESEIQSALQKAELAANE

TNRASIVALNAEIRRIKGRLLEEVPKLQRLAQKRVKGLSIEELGARNDLVLALPDRIQAI

PDGTPTSKKNGGWTSGWTSSATRQEIKFDSDGRFDSEYLQETEQSSQFRQEYEMKKMKQD

QGLDMIAEGLDTLKNMAHDMNEEMDRQVPLMDEIDTKVDKATSDLKNTNVRLKDTVTQLR

SSRNFCIDIILLCVILGIAAYLYNVLKK*

R-47 homolog

>Bevul.4G018100.1.p

MGNHSPNPPLSQGLMASTSGDSWMKEYNDATKLADDINGMISEKTAMSGSGPDSQRHFSA

VRRKITILGTRLDSLESLLSKPPSKKSITEKELNHRKDMLSNLRTKTNQMATTLNMSNFA

NRDSLLGPESKPVDAMTRVAGLDNQGIVSLQRQIMREQDEGLEQLEETVMSTKHIALTVN

EELELHTRLIDNLDQHVEFTGSRLQRISRSLANLNKKTKGGCSCLCLLVAVAGIVILILA

IFVLIKYL*

R-48 homolog

>Bevul.3G112700.1.p

MATRGGAAPYRSSDGLSTRQVGNSDEIQLRIDPMHGDLDDEILGLRSQVHKLRNVAQEIH

SEAKFQSDFVDQLQMTLIRAQASLKNNMRRLNSSIVRSGSNHVLHVVIFALFCLFLVYFW

SKFSRR*

R-49 homolog

>Bevul.8G100000.1.p

MAAPDLLFNLRNNFYLGAYQAAINNSEIPNVSPDEAVERDCLVYRSYIALGSNQLVISEI

DASAATPLQAVKLLALYLSASENKETTISSLKEWLADPAIGNIPTLRLVAGVIFMHEQDY

SEALKHTNAGGTMELHALNVQIYLKMYRSEHAEKQLRLMQQIDEDHTLTQLANAWLNLAV

GGSKIQEAYLIFQDFSEKYPMTGLILNGKAVCCMHMGNFDEAESLLLEALNKDAKDAETL

SNLVVCCLHLNKPTGRYLSQLKGTHPEHMFVKRLATAEESFERAVQSVA*

R-50 homolog

>Bevul.2G179100.1.p

MLLAVLISNAEGNILVERFNGVPSEERLHWRTFLVKLGAENLKDAKNEELFVASHKSVYV

VYTVLGDIHIYLVGKDEYDELALSEAIFIITSTLKDVCGKPPNERLFLDKYGRICLCLDE

IVWTGVLENTDKERIKRLVRLKPPTEF*

R-51 homolog

>Bevul.1G097300.1.p

MDGIINKIRNLDAYPKINEDFYSRTLSGGLITLVSSFVMLLLFFSELSLYLNAVTETQLV

VDTSRGETLRINFDVTFPALPCSILSLDAMDISGEQHLDVRHDITKKRLDSHGNALETRQ

EGIGAPKIEKPLQRHGGRLEHNETYCGSCYGAEAADEDCCNSCEDVREAYRKKGWAVSNP

DLIDQCKREGFLQKVKDEEGEGCNIYGFLEVNKVAGNFHFAPGKSFQQSGVHVHDLLAFQ

KDSFNISHTINRLTFGDYFPGVVNPLDGVHWTHNTPNGMYQYFLKVVPTVYTDVNGHTIN

SNQFSVTEHFKSASAGHFSTLPGVFFFYDLSPIKVTFKEEHVSFLHFLTNVCAIVGGVFT

VSGIIDSFVYHGQKAIKKKMEIGKFS*

R-52 homolog

>Bevul.4G165200.1.p

MKFPIFAFVFILPLIFLQTLGITSAAPIISNHNSIQDTLINCLNDHSVPSHPISPQIYAP

QNPSFSTVLQSYIRNLRFNESTTRKPLFIITATHVSHIQAAIICAKQHGIEMKVRSGGHD

YEGLSYVSIVPFFMVDMFNFRSIDVNIDEETAWVDAGSTLGEVYYNIGNNSRIHAYPAGV

CPTVGSGGHITGAGYGNLMRKYGLTVDNLIDAKIVDVNGRILDRATMGEDLFWAIKGGGG

ASFCVILSYKIKLVRVPERVTVFRVSRLLEQNLTNIVDQYQHVAPNLDHNAFIRLTLGVT

NSSETGLLTNQATFRCLFLGDTESLISLVHENFPLLGLEKSDCLEMSWLESILFYSDYSM

GTPVETLLDRQPPSVTYLKRKSDYVKKPIPRYGLEGLWKIMVELQPLQLTFNPYGGRMAE

IAANATPFPHRAGNLFKLQYAINWNEGGSERAKYYIDLTRKLHAYMTPFVSKNPREAFLN

YRDLDLGINHQGSRSYVEGKKYGVMYFKDNFDRLVRIKSMVDPDNFFRNEQSIPTLPR*

R-53 homolog

>Bevul.3G155800.1.p

MLSWCIGQRRVAQIQRSFHKMKVTILCLLLTLIMIRSMLGAGEYGTPKQDFDLLHSSLAS

AGSHHTHNRRILEELKDADDKKSNTNSNSYESFDISKILVDDEPAEKVDPNKPYSLGPKI

SDWDEQRREWLEKNKEFPNFVQPNKPRVLLVTGSSPKPCENPVGDHYLLKSIKNKIDYCR

IHGIEIFYNLALLDAEMAGFWAKLPLIRKLLLSHPEIEFLWWMDSDAMFTDMVFELPWER

YKDYNMVMHGWNEMIYDEKNWIGLNTGSFLLRNNQWSLDMLDTWAPMGPKGKVRDEAGKI

LTRELKGRPVFEADDQSAMVYILATQREKWGNKVYLENHYYLHGYWGILVDSYEEMIEKY

RPGFGDHRWPLVTHFVGCKPCGKFGDYPVERCLKQMDRAYNFGDNQVLQMYGFSHKSLAS

RKVVKIRNETSNPLEVKDELGLLHPTFKALKV*

R-54 homolog

>Bevul.4G169500.1.p

MAPPNLEPATTKPTLGSVGKHNMGRAYVTFLAGNGDYIKGVVGLAKGLRKARAAYPLVVA

VLPDVPEEHRRILVSQGCIVREIEPVYPPDNQTQFAMAYYVINYSKLRIWEFVEYTKMIY

LDGDIQVYDNIDHLFDLPNGYFYAVMDCFCEKTWNHTPQYKIGYCQQSPNRVCWPEEELG

PKPAKYFNAGMFVFEPSLSTYLDLLNTLQVTPPTPFAEQDFLNMYFKDIYKPIPSIYNLV

MAMLWRHPENINLEEVKVVHYCANGSKPWRYTGKEENMDREDIKLLVNKWLDIYNDESLD

YKPEVEAHRRKLQPFKAALSEAGILHYVSAPSAA*

R-55 homolog

>Bevul.4G043200.1.p

MAEEESSCCRCCISFIFTSGLTALFLWLSLRPSNPTCSIENFDVFILNKTANSTFKNNHT

ILYDLKLNNKGNKDKGIYYDTLNLTFYYKPNLTLINTLGNATYVPFYQGYGKNTHRKGSI

DARGVKWENATATTAVFRVELNTLVRFKIVFWKTKRHRLDLKADLLVNEQGSLIKRKKKK

GIKLTSGVGNSMSCSKFLLLGVLGVLIFIHFW*

R-56 homolog

>Bevul.9G059700.1.p

MENEAKGKEVRGVPTHGGSYVLYNVLGNLFEVSSKYAPPIQPVGRGAYGIVCCARNSETN

EEVAIKKIADAFNNRIDAKRTLREIKLLCHMDHDNVIKIKDIILPPEREKFNDVYIVYEL

MDTDLHQIIRSNQELTEDHCQYFLYQLLRGLKYIHSANVLHRDLKPSNLLLNANCDLKIC

DFGLARTTAETDFMTEYVVTRWYRAPELLLNCSEYTAAIDMWSVGCIMMEIIKREPLFPG

KDYLQQLQLINELLGSPEDSDLGFLRSDNAKRYVKQLPHFPKQPFAQKFPNVSPLVLDLA

EKMLVFDPSKRITVDDALNHPYLLSLHEINEEPTCPKPFQFDFEQSSLDEEDIKELIWRE

ALKFNPR*

R-57 homolog

>Bevul.5G026700.1.p

MDCFEALHGEANQEGKIKGVMTHGGRYVQYNVHGSLFEVSSKYVPPIRPIGRGASGLVCA

AVNSDTHLEVAIKKIGNAFDNIVDAKRTLREIKLLRHMDHENIIAIKDIIRPPKKEAFND

VYIVYELMDTDLHQIIRSNQSLTDDHCQYFLYQLLRGLKYVHSAKVLHRDLKPSNLLLNA

NCDLKIGDFGLARTTTETDFMTEYVVTRWYRAPELLLNCSEYTAAIDVWSVGCIFGEIMT

REPLFPGKDYVHQLRLITELIGSPDDSSLGFLRSNHARRYVKQLPQYPKQQFSARFPNMS

PAATDLLEKMLVFDPNKRITVDEALCHPYLSSLHDINDEPICSAPFRVDFEQTSLTEENV

KELIWKESVKFNPDPAH*

R-58 homolog

>Bevul.4G084300.1.p

MQQDQRKKGLKDTEFFTDYGEANRYKILEVIGKGSYGVVCAAVDTHTGEKVAIKKINDIY

EHISDAIRILREVKLLRLLRHPDIVEIKRIMLPPSKRDFRDIYVVFELMESDLHQVIKAN

DDLTREHHQFFLYQMLRALKYMHTANVYHRDLKPKNILANANCKLKICDFGLARVAFNDT

PTTIFWTDYVATRWYRAPELCGSFFSKYTPAIDIWSIGCIFAEVLTGKPLFPGKSVVHQL

DLITDLLGTPSHDVISGVRNDKARKYLTDMRKKQPVPFSQKFPNADPLGLRLLQRLLSFD

PKDRPTAEEALADPYFKGLARMEREPACHPISKLEFEFERRRVTKEDVRELIYREILEYH

PQLLKDYMNGNEGSNYIYPSAIGQFKKQFAHLEENSGRSGPVIPLERKHISLPRSTVHCS

TIPPKPQPLASSQRDHQVKEEMRTSESYGSLSSSSRQPPRGPSARPGRVVGPVVSYEAGR

SMKDSYDARISMQNAVLPPQTTSASSQYYFRPNVTPPPQTKPGTENEQGRTSWAKQQPLP

VQSTHEVAIDINTNPYHQPQPKPDQYSSRIALDAKILQAQSQFGATAVAMAAHRNVGTVQ

YGMS*

R-59 homolog

>Bevul.3G132300.1.p

MQQDQRRKTSADVDFFTEYGEGNRYKIEEVIGKGSYGVVCSAYDTHIGEKVAIKKINDIF

EHVSDATRILREIKLLRLLRHPDIVEIKHILLPPSRREFKDIYVVFELMESDLHQVIKAN

DDLTPEHYQFFLYQLLRGLKYIHTANVFHRDLKPKNILANADCKLKICDFGLARVAFNDT

PTAIFWTDYVATRWYRAPELCGSFFSKYTPAIDIWSIGCIFAELLTGKPLFPGKNVVHQL

DLMTELLGTPSPDAIARIRNEKARRYLSSMRKKKPIPFTHKFPNADPLALRLLERMLAFE

PKDRPTAEEALADPYFKGLAKVEREPSAQPVTKMEFEFERRRMTKEDVRELIYREILEYH

PKMLKEFLDGSEPTSFMYPSAVDHFKKQFAYLEEHYKNGSTGAPIDRQHASLPRACVSYS

DNSVHSSVEVTDDLSKCRIKEVEKPHVDRNGAIPMTRLPLQVPQNVQGAAARPGKVVSNL

LRYNNCGAAATAEVMDRRAVRNPSVPSQFPTSSYPKRNPGCKNEREDEPMEGSNSVQPKP

PYLARKVAAAQGVPGSHWY*

R-60 homolog

>Bevul.9G059400.1.p

MSEVAQNQMAAGLSPEFPTAFTHGGQFIQYNIFGNLFEVTSKYRPPIMPIGRGAYGIVCS

VLNSETKEMVAMKKIANAFDNYMDAKRTLREIKILRHFDHENIIALRDVVPPPLRREFSD

VYVATELMDTDVHQIIRSHQSLSEEHCQYFLYQILRGLKYIHSANVIHRDLKPSNLLINA

NCDLKICDFGLARPTSENEHMTEYVVTRWYRAPELLLNSSDYTAAIDLWSVGCIFMELMN

RRPLFPGKDHVHQMRLLTELLGTPTEADLGFLHNEDARRYIRQLPPQPRQQLRQVFPHVN

PLAIDLIEKMLTFDPTRRITVEEALSHPYLARLHDIADEPICQKPFSFEFEQQALGEEQM

KDMIYEEALALNPGYA*

R-61 homolog

>Bevul.1G157100.1.p

MATQVEPPNGVRCPGKHYYSMWQALFEIDTKYVPIKPIGRGAYGVVCSSINKETNEKVAI

KKIHNAFENRVDALRTLRELKLLRHLRHDNVIALKDVMLPAHLKNFKDVYLVYELMDTDL

HQIIKSSQALTNDHCQYFLFQLLRGLKYLHSANILHRDLKPGNLLINANCDLKICDFGLA

RTSSGKGQFMTEYVVTRWYRAPELLLCCDKYGTSIDVWSVGCIFAELLGRKPLFPGTECL

NQLKLIVNILGSQKDFDLEFIDNPKAKRFIKSLPYSPSTSLSRLYPTAHPLAIDLLQKML

VFDPTKRISVTEALQHPYMSALYDPRANPPAQFPIDLEIDEDLDEDMIRETMWQEIMHYH

PEAVNPNMDVCG*

R-62 homolog

>Bevul.3G114700.1.p

MEASAAQPSDTEMSDAGIPAPENPLPDHSQPPQQQQQQQISIPATLSHGGRFIQYNIFGN

VFEVTVKYKPPIMPIGKGAYGIVCSALNSETNEHVAIKKIANAFDNKVDAKRTLREIKLL

RHMDHENVVAIRDIIPPPRREAFNDVYIAYELMDTDLHQIIRSNQGLSEEHCQYFLYQIL

RGLKYIHSANVLHRDLKPSNLLLNANCDLKICDFGLARVTSETDFMTEYVVTRWYRAPEL

LLNSSDYTAAIDVWSVGCIFMELMDRKPLFPGRDHVHQLRLLMELIGTPSEHELGFLNEN

AKRYIRQLPQYRRQSLAEKFPHVNSAAIDLVEKMLTFDPRQRITVEDALAHPYLNSLHDI

SDEPICMTPFSFDFEQHALSEEQMRELIYREALAFNPEFHPE*

R-63 homolog

>Bevul.5G026700.1.p

MDCFEALHGEANQEGKIKGVMTHGGRYVQYNVHGSLFEVSSKYVPPIRPIGRGASGLVCA

AVNSDTHLEVAIKKIGNAFDNIVDAKRTLREIKLLRHMDHENIIAIKDIIRPPKKEAFND

VYIVYELMDTDLHQIIRSNQSLTDDHCQYFLYQLLRGLKYVHSAKVLHRDLKPSNLLLNA

NCDLKIGDFGLARTTTETDFMTEYVVTRWYRAPELLLNCSEYTAAIDVWSVGCIFGEIMT

REPLFPGKDYVHQLRLITELIGSPDDSSLGFLRSNHARRYVKQLPQYPKQQFSARFPNMS

PAATDLLEKMLVFDPNKRITVDEALCHPYLSSLHDINDEPICSAPFRVDFEQTSLTEENV

KELIWKESVKFNPDPAH*

R-64 homolog

>Bevul.9G059400.1.p

MSEVAQNQMAAGLSPEFPTAFTHGGQFIQYNIFGNLFEVTSKYRPPIMPIGRGAYGIVCS

VLNSETKEMVAMKKIANAFDNYMDAKRTLREIKILRHFDHENIIALRDVVPPPLRREFSD

VYVATELMDTDVHQIIRSHQSLSEEHCQYFLYQILRGLKYIHSANVIHRDLKPSNLLINA

NCDLKICDFGLARPTSENEHMTEYVVTRWYRAPELLLNSSDYTAAIDLWSVGCIFMELMN

RRPLFPGKDHVHQMRLLTELLGTPTEADLGFLHNEDARRYIRQLPPQPRQQLRQVFPHVN

PLAIDLIEKMLTFDPTRRITVEEALSHPYLARLHDIADEPICQKPFSFEFEQQALGEEQM

KDMIYEEALALNPGYA*

R-65 homolog

>Bevul.9G221400.1.p

MNLTKNLASFLCFFFATLALFHMCHAQNSPQDYVNAHNAARAAVGVGNIQWDDQVAAFAQ

QYANQRKADCALRHSGGGGRYGENIAVSSGSSMTGTAAVKLWVDEKAFYNYNTNTCASGK

VCGHYTQVVWRNSNRLGCARVQCNNGGVFVTCNYSPPGNYIGQKPY*

R-66 homolog

>Bevul.9G018500.1.p

MEMASSSGSKNEFGLQRPPSRRMTRTPTMIDPTKDESVPIDSELVPSSLAVIAPILRVAN

EVEKENPRVAYLCRFHAFEKAHKMDPTSSGRGVRQFKTYLLHRLEKEEAETRPVLAKSDP

REIQKFYQNFCEKNIRLGQHTKTPEEMAKIYQIATVLYDVLRTVVPYAKVDEETENYAKE

VERNREQYEHYNILPLFAVGVKPAIMELPEIKAAIRAIRTLENLPMPRVSPAPITQDDNV

IMPEYRDKSINDMLDWLASIFGFQKGNVANQREHLILILANMDVRIRRSEEYEVLDFQTI

HQLKEKIFKNYERWCDYIHCKSNLKFPPGADLQQLELLYIALYLLIWGEASNVRFMPECL

CYIFHNMASEIHGILYSNVHPITGDTYQSTRHGDESFLKEVIAPIYDVVRKEARRNKGGK

ASHSAWRNYDDLNEYFWSQKCFKLGWPMDRNADFFVHTDETRPSSTRHDQVAIGKRKPKT

NFVEMRTFWHLFRSFDRMWILFLLAFQTMVIVAWSPSGSITSIFDPDVFESVLSIFITAA

FLNFLGATLDIFLSWKAWGSLKFHQILRYFLKFVVAAMWAIVLPIGYTSFVQNPAGIVNF

LTSWAGDLRSPLFFKFAIALYMAPNILAALLFFFPPIRKFVERSNSRIIILIMWWAQPKL

YIGRGMHEDTFSLLKYTLFWILLLMCKLSFSFYVEILPLVGPTKLIWRMKVDDYQWHEFY

PNATHNFGVIIAIWAPVVLVYLMDTQIWYAIFSTIVGGILGAFSHLGEIRTLGMLRSRFE

SVPLAFRKRLVPRLKGETKQGSADVLEARKNIAKFSQVWNEFIHSLRLEDLISHRERDLL

LVPYTSSKVSVVQWPPFLLASKIPIALDMAKDFKKKHDSELFNKIKDDDYMYSAVIECYE

TLREILFELLEDKDDKLAIKQICEKIETSIQQQKFLTEFRMNGLPMLHDKMEKFLKLLLS

DSDYDDEDLYKSHIINVLQDIVEIITQDVMSDEHDILKRPQLHHQIDDDGKREQRFEKIH

IFLLRNKSWREKVVRLYVLLSEKESAINVPMNLEARRRMTFFTNSLFMTMPSAPFVRNML

SFSVLTPYYKEDVLYSWEELHEENEDGISTLFYLQKIYPDEWSNFYERINDPKLGYASKD

SKELTRHWVSYRGQTLSRTVRGMMYYRQALDLQCFLEYAEDKAIFSGYRTIEKSEAHKKI

FDYSQALTDLKFTYVVSCQVYGNQKKSSDARDRSCANNILNLMLTYPSLRVAYIDEREEK

VEGKSEKVYYSVLVKGGDKLDEEIYRIKLPGPPTEIGEGKPENQNHAIIFTRGEALQTID

MNQDNYFEEAFKMRNVLQEFQKSRRKRRKPTILGLREHIFTGSVSSLAWFMSNQETSFVT

IGQRVLANPLRVRFHYGHPDIFDRLFHITRGGLSKASKIINLSEDIFSGFNSTLRGGYIT

HHEYIQVGKGRDVGMNQISLFEAKVANGNGEQTLSRDVYRLGRRFDFYRMLSFYYTTVGF

YFSSMVTVLTAYIFLYGRLYTVLSGLEKSIIESATINQAKALEQALAPQSMFQIGVLLVL

PMIMEIGLERGFRTAIGDFIIMQLQLASVFFTFQLGTKAHYYGRTILHGGSKYRATGRGF

VIFHAKFADNYRRYSRSHFVKALELFILLIVYQAYGDSYRSSNLYLFVTWSMWFLVASWL

FAPFIFNPSGFDWQKTVDDWTDWKRWMGNRGGIGIQTDKSWESWWDGEQEHLKHTTVRGR

FLEIVLACRFFLYQYGIVYHLDISHGSRSFLVYALSWVVMGTALLVLKMVSMGRRRFGTD

FQLMFRILKGLLFLGFVSVMTVLFAVFNLTIKDLFASILAFFPTGWAMLLIGQTCRGLLK

GIKFWESIKELARAYEYVMGLIILTPIAVLSWFPFVSEFQTRLLFNQAFSRGLQISMILA

GRKDRSAPNMQGP*

R-67 homolog

>Bevul.4G243500.1.p

MSRAEELWERLVKVTLRNARDGAAAHGRHTTGIAGNVPSSLANSRVIDDILRIADEVQDE

DPNVARILCEYAYSVAQNLDPNSEGRGVLQFKTGLMSVIKQKLAKREAGNIDRSLDIARL

QEFYRKYRRMNNVDQLLEDEMKLRESGAFTGNLELERKTKKRKRIFATLKVLGEVLEQMT

QEVSPEEAETLIPEELKRVMESDAAMTEDLIAYNIIPLDTTSTTNRISTFPEVRAAVSAL

KVFKGLEELPAEFVSPVRGRDMLDFLHFVFGFQKDNVSNQREHIIHLLANEQSRLRIPEE

FEPILDEAAVQRVFMKSLENYIKWCNYLVLLPVWSNLESLSKEKKLLFVSLYYLIWGEAS

NVRFLPECLCYIFHHMAREVDEILRQQVVKQADSCMTDDGVSFLEKVISPLYDVIAAEAA

NNDNGRAPHSKWRNYDDFNEYFWSMHCFELSWPWRKSSSFFMKPERKSKNLLKSGGGKRH

GKTSFVEHRTFLHLYHSFHRLWIFLVMTFQALAIIAFNDGNINSTTIKQVLSLGPTFVIM

KLFESVLDILMMFGAYSTTRHIAVSRVFLRFVWFACASAVITFLYVKSIQGVATIYRIYV

IVIGIYAGVQFFISFLMRIPACHQLTDRCHRWSLIQFTKWLRQERHYVGRGMYERTSDFT

KYMFFWIIILAGKLSFGYFVQIRPLVNPTRIIVDMNNIRYSWHDFVSQNNHNALTVLSIW

APVFCIYLLDIYVFYTIVSAIWGFLLGARDRLGEIRSLDALHQLFEQFPRAFMDKLYFLP

FKSLFFYLIKVTETSKEDAARFSPFWNEIIKNLREEDYITNLEMELLLMPKNSGNLPLVQ

WPLFLLASKIFLARDIAAESRDSQEELWDRIFRDEYMKYAVIECYHSIKLILMEILEEDG

RKWVERVYEDIEECVAKKSIHVDFELNKMSLVIQKVTALMGVLKGSGTADLEKGAVKAIQ

DLYDVIRYDVLAVNMRDHMQTWNALSKARAEGSLFSKLKWPKDPELKAQIKRLYSLLTIK

DSASNIPKNLEARRRLQFFTNSLFMDMPPVKAVREMMSFSVFTPYYSETVLYHMDELRKK

NEDGISILFYLQKIYPDEWKNFLARIGRDENAQETDLFDSPNDVEELRFWASYRGQTLAR

TVRGMMYYRKALMLQTYLEKIASGDLEAPISGNDSLNVQGFELSPEARAQADLKFTYVVT

CQIYGKQKEEGKPEAADISLLMQRNEALRVAFIDVVETMRDGKVQTEYYSKLVKADINGQ

DKEIYSVKMPGNPKLGEGKPENQNHAVIFTRGQAIQTIDMNQDNYFEEALKMRNLLEEFN

HDHGIRPPTILGVREHVFTGSVSSLASFMSNQESCFVTLGQRVLANPLKVRMHYGHPDVF

DRVFHVTRGGISKASRVINISEDIYSVPCFYQADIFFYNLQLTVLTVYIFLYGKTYLALS

GVGQTIQDRAHISQNTALSAALNTQFLIQIGIFTAIPMILGFILEQGFFRAIVSFITMQF

QLCSVFFTFSLGTRSHYFGRTILHGGARYQATGRGFVVRHIKFSENYRLYSRSHFVKGLE

VVLLLIVFLAYGYNDGGAIGYILLSISSWMMALSWLFAPYLFNPSGFEWQKTVEDFSDWT

NWLLYRGGIGVKGEESWEAWWEEELGHIRTFGGRVAETILSLRFIIFQYGIVYKLHVQGT

NTSFAIYGFSWAALAGIIILFKVFTFSQKASVNFQLLLRFVQGITFLMALAGIAVAVVLT

DLSVPDIFASILAFIPTGWMILSIAIAWKPYVKKVGLWKSIRSLARLYDAGMGMLVFIPI

AFFSWFPFVSTFQTRLMFNQAFSRGLEISLILAGNNPNQGL*

R-68 homolog

>Bevul.8G026500.1.p

MNRRPRANPTRPYPHAPPPPTTTHQPFNIIPIHDILSNHPSTRYPEVRAASSSLRAVEDL

RKPPFHTWHDNYDLLDWLGLFFGFQNDNVRNQREHLVLHLANSQMRLTPPPQNAAVLEFD

LVRKFRKKLLTNYSRWCSYVGKKSRVRIHRLRKNTDEFRRELLYVSLYLLIWGESANLRF

MPECICYIYHHMAHELNLILDDRDDPETGRPYLPVISGENGFLNYVVKPLYDTIKDEVGF

SKGGSKPHSAWRNYDDLNEFFWSRRCFRSLKWPLDLSSTYFSSASKGTRVGKTGFVEQRS

FWNVFRSFDRMWVFLLLFLQAMLIVALEGTKWPWEAVGRRDVQVKLLTVFITWAGLRLLQ

SVLDAGTQYSLVTRETWLLGVRMVLKCMVAMAWIVVFSVFYARIWSQNERDRGRWLYQGE

QRILMFLKAVFVFVIPELLAVVLFLLPWLRNFLEEVNFPLFHLLTWWFHRRIFVGRGVRE

GLLNNVKYSSFWVTVLVSKFAFSYFFQIKPLIDPTRALLRLRNVEYNWHEFFSNTNRIAV

VMLWLPVIIIYLVDLQIWYSIYSSVVGAYVGLFSHLGEIRNIWQLRLRFQFFASAMQFNL

MPEEQLPTVPVINVVHKLRDAVKRLKLRYGLGQPYKKIESSQVEATRFALLWNEIMKYMR

EEDLLSDREVELLELPPNCWNIRVVRWPCILLCNELLLSVCQAEATANVHDRSLWYKICN

SEYRRCAIIEAYDSIKHLLLEQVVKYNTEEHAIVTRAFLEMEHYMEIEKFTAFYNLTLLP

RIHKKLLTLIELLQRAEKDTNGIINVLQALYELCVREFPRIKRTMVELREQGLALKNPAG

FLFANAVEFPDEEDVFYYRQLRRLHTLMTSRDSMHNVPMNREARRRIAFFSNSVFMNMPH

APQVEKMLAFSVLTPYYDEDVIYGTSKLQSANEDGISILFYLQKIYEDEWNNFMERMRRE

GLEDEREIWTTKVKELRLWASYRGQTLSRTVRGMMYYYHALKMLAFLDSASEVDIRTGCQ

QIASHGSLSLSNNWDGLGSIRVPSSRNLGRTPSGVNILYKGNEFGTAMMKFTYVVTCQVY

GIHKMAGDSRAEEILYLMKNNEALRVAYVDEVHLGRDEVEYFSVLVKYDQQLQREVEIYR

IKLPGPVKLGEGKPENQNHAIVFTRGDALQTIDMNQDNYFEEALKIRNLLEEFNRNYGIR

KPTILGVRENIFTGSVSSLAWFMSAQETSFVTLGQRVLANPLKVRMHYGHPDVFDRFWFL

TRGGVSKASKLINLSEDIFAGFNCTLRSGDVTHHEYIQVGKGRDVGLNQIAMFEAKVASG

NGEQVLSRDVYRLGHRLDFFRMLSVFYTTVGFCFNTMMVVMSIYMFLWGRLYLALSGVER

HALRFRNTSNNAALGAIVNQQFIIQIGLFTALPMIVENSLEHGFLPAIWDFLKMQLQLAS

VFYTFSMGTRSHYFGRTILHGGAKYRPTGRGFVVQHKKFAENYRLYSRSHFVKAIELGVI

LTVYASHSPLATNTFVYIAMSVSSWFLVFSWIMAPFIFNPSGFDWLKTVYDFDDFITWIW

YGSGVSTKAERSWETWWYEEQEHLKTTGLWGKLLEIILDLRFFFFQYSIVYQLNITANRT

SIGVYFISWLYFFGLVGICVIVAYARDKYGARKHIYFRIIQSSVISLTVLVIIILLRFTR

FQVMDLLTSALALIPTGWAMILIAQVLRPFLQTSVVWDTVVSLARLYDLTFGIIVMAPVA

FLSWMPGFQSMQTRILFNEGFSRGLQISQLLTAK*

R-69 homolog

>Bevul.4G243400.1.p

MSSPSTSRGGGSDQAPARRLMRTQTAGNLGETAFDSEIVPSSLNEIAPILRVANEVEKHN

PRVAYLCRFYAFEKAHRLDPTSSGRGVRQFKTALLQRLEKENDPTLMGRVKKSDAREMQS

FYQHYYKKYIQALQNAADKADRAQLTKAYQTANVLFEVLKAVNLTQAMEVDREILDTHNK

VAEKTEIYAPYNILPLDPDSANQAIMRYPEIQAAMVALRNTRGLPWPKDYKKKQDEDILD

WLQAMFGFQKDNVANQREHLILLLANVHIRQFPKPDQQPKLDERALTEVMKKLFKSYKKW

CKYLDRKSSLWLPTIQQEVQQRKLLYMGLYLLIWGEAANLRFMPECLCYIYHHMAFELYG

MLAGNVSPRTGENVMPAYGGEEEVFLRKVVTPIYDVIRREAESSKKGKSKHSQWRNYDDL

NEYFWSVDCFRLGWPMRIDADFFCMSVQQLHQDKVGESKAGDRWTGKVNFVEIRSFWHIF

RSFDRMWSFFILALQAMIIVAWNGTGDPSAIFETNVFKNVLSIFITSSILTLGHAALDVV

LGWKARRTMTFHVKLRYILKVVSAAAWVVILPVTYAYSWDNPPPGFAQTIKSWFGSNAHS

PSLFFLAVVIYLSPNMLSAMLFLFPSIRSLLERSNYKIVMLMMWWSQARLFVGRGMHESA

FSLFKYTMFWVLLLLTKLAFSFYIEIKPLVAPTKDIMSVHITRFKWHEFFPQARSNIGAV

ISLWAPIILVYFMDTQIWYAIFSTLFGGIYGAFRRLGEIRTLGMLRSRFQSLPGAFNARL

MPAEKTEHKKKGLKATFSRRFPDIPSNKEKEAARFAQLWNQIITSFREEDLISDREKDLL

LVPYWADRDMDLIQWPPFLLASKIPIALDMAKDSNGKDRELIKRIAADPYMPCAVHECYA

SFKSVIKHMVKGGPEKIVIENIFAEVEKHIGEGDLVTEYRMSALPSLYGHFVKLMKCLLE

NKKEDKDQVVLYFQDMLEVVTNDIMEDTSVLESIHGGSLDSGMASFELFASEGAINFPIE

PITEAWKEKIKRLYLLLTVKESAMDVPSNLDARRRLSFFSNSLFMDMPPPPKVRNMLSFS

VLTPYYTEEVLFSLQALETQNEDGVSILFYLQKIFPDEWENFLERVNCKNEEELRDNEEL

EEDLRLWASYRGQTLTRTVRGMMYYRKALELQAFLDMANDKVLMEGYKALELNTEENSQS

ERLLCQAVADMKFTYVVSCQRYSIHKRSGDPRAQDILKLMTTYPSLRVAYVDELEERSED

GSKKTNKIYYSALAKAALASSTNSNEAGQNLDQVIYRIKLPGPPILGEGKPENQNHAIIF

TRGEGLQTIDMNQDNYMEEALKMRNLLQEFLKKHGGVRYPTILGLREHIFTGSVSSLAWF

MSNQETSFVTIGQRLLANPLKVRFHYGHPDVFDRLFHLTRGGVSKASKVINLSEDIFAGF

NSTLRGGNVTHHEYIQVGKGRDVGLNQISMFEAKIANGNGEQTVSRDIYRLGHRFDYFRM

LSCYFTTVGFYFSTLITVLTVYIFLYGRLYLVLSGLEEALNSQRRFRNNKPLQVALASQS

FVQIGFLMALPMLMEIGLERGFRTALSEFILMQLQLAPVFFTFSLGTKTHYYGRTLLHGG

AKYRSTGRGFVVFHAKFADNYRLYSRSHFVKGIELMILLIVYEIFGQTYRSPIAYIFITI

SMWFMVGTWLFAPFLFNPSGFEWQKIVDDWTDWNKWISNTGGIGVPPEKSWESWWEEEQE

HLRHSGIRGIIAEILLSLRFFIYQYGLVYHLKITEKTRSFLVYGLSWLVIMLILFVMKTV

SVGRRKFSADFQLVFRLIKGLIFLTFVGILVTLIVLPGMTLQDIIVCILAFMPTGWGLLQ

IAQALKPVVHKAGFWGSVRTLARGYEIVMGLLLFTPVAFLAWFPFVSEFQTRMLFNQAFS

RGLQISRILGGHRKDRTSRNKD*

R-70 homolog

>Bevul.2G057700.1.p

MAEISHLIMFSLLSLSLFNFSFALNPQVSYISNHQHHSKYHPKVNQEIGVYELKNGNVSV

KLTNYGATVISVIVPDKYGKLADVVLGYDSAKEYVNDTTYFGAIVGRVANRIAGAQYTYN

EKHYKLIANEGKNILHGGPKGFSRVIWKVDRYSPKAHYPHIKFSYFSFDGEQGFPGKLKV

TVIYKIIKGNHLLVTMRAKPLNKPTPVNLAQHTYWNLGGHTSGNILAHEVQIFASQITPV

DKNLIPTGKFEQVKGTPFDFLKPHTVGSTINKLPKGYDINYVVDGYKTRHAAVTPVAVVK

EKKSGRVMELWANKPGVQFYTSNTLNTTGKGGHVYGPHAALCLETQGFPDAVNHPNFPSV

MVSPGETYEHYMLFKFSTMN*

R-71 homolog

>Bevul.8G025400.1.p

MGSIHLLQACAILISLLVIVIPSSFTATSAELLPIFHSHPYSFAPSLAPSPAPSHHHHHH

HNHHSPSPSPAYSPAKPPTSAHAPAHSPYSAKPPTKAHHHAHAPYYSAKPPTKAHAPAPY

YSAKPPTKAHAPGHYSAKPPTKAHAPAPYSAKPPTKAPAPYSKPLPRKLVAVQGVVYCKN

CSYAGVDTLLGASPLSGATVELRCRNTKYTIKKTSTTDKNGYFFLQAPKYITTYGVHKCR

VFLIEMPKKGGPCSHATNLNGGVSGAFLFFNKKLTPPPKPLPFSLFTVGPLAFEPPKCYK

H*

R-72 homolog

>Bevul.8G161800.1.p

MGFLGKFMIFIVVICLICESGNGFYLPGSYPHKYVVGDTLSVKVNSLTSIDTEMPFSYYS

LPFCKPQEGVKDSAENLGELLMGDRIENSPYRFKMYTNETEIFLCKTSPLSADEFKLMKK

RIDEMYQVNLILDNLPAIRYTKKDNFLLRWTGYPVGIKVQDAYYVFNHLKLNVLVHKYEE

PNVARVMGTGDAVEMIPNGANGPVEPGYMVVGFEVVPCSVQHDMNAIKNSKIYDKYPGKI

NCDPATVSMAIKESQPVAFTYEVSFVESDIKWPSRWDAYLKMEGAKVHWFSILNSLMVIT

FLAGIVLVIFLRTVRRDLTRYEELDKEAQAQMNEELSGWKLVVGDVFRAPGYASLLCVMV

GDGIQLLGMGVVTIMFAALGFMSPASRGTLLTGMVFFYLVLGIAAGYVAVRLWRTIGCGD

HKGWVSVSWKAACFFPGVAFLIMTILNFLLWGSHSTGAIPFSLFVILILLWFCISVPLTL

VGGYFGAKAPHIEYPVRTNQIPREIPPQKYPSWLLVLGAGTLPFGTLFIELFFIMSSLWM

GRVYYVFGFLFIVLILLVVVCAEVSLVLTYMHLCVEDWKWWWKSFFASGSVAIYIFLYSI

NYLIFDLKSLSGPVSATLYLGYSLFMVLAIMLVTGTVGFLSSFWFVHYLFSSVKLD*

R-73 homolog

>Bevul.4G021700.1.p

MIFCFVFAADTGKIGINYGRIANDLPTPVKVVQLLKTQGVSRIKLYDTDSDVLKAFSNSG

ISLTVALPNENLTAAAASQSFTDTWVQTNIVPFHPGSNLVAIAVGNEANVDPNITSYVTP

AMSNVHASLQKYSLDDKIKISSPISFSALQNSYPSSSGSFKQELLEPFIKPMLEFLKKTS

SYMMVNAYPFFAYSSNSDEISLDYALFKPNAGVVDSGNGLKYESLFEAQLDAVYAAMDAL

KFNDLKIVVTETGWPSQGDENEVGASGPNAAAYNGNLAKRVLTGGGTPLRPNDPLDVYLF

ALFNENQKPGPTSERNYGLFYPNEQKVYNVPLTAAQIGDAAVAPASANNGSKVVVPPTGQ

TWCVANEQAGEEKLQDGLNWACGEGKADCRPIQPGATCYNPGTLVAHASYAFNSYYQMKA

RSTGTCDFGGAAHVVTQSPQFGSCKFPTGY*

R-74 homolog

>Bevul.7G175500.1.p

MASCLKSRKQVILLLVSVLLAYGVSELQAARSPPTNQPYRTAYHFQPRKNWINGPMLYKG

VYHLFYQYNPNGVIWGPPVWGHSTSKDLVNWVPQPLTMEPEMAANINGSWSGSATILPGN

KPAILFTGLDPKYEQVQVLAYPKDTSDPNLKEWFLAPQNPVMFPTPQNQINATSFRDPTT

AWRLPDGVWRLLIGSKRGQRGLSLLFRSRDFVHWVQAKHPLYSDKLSGMWECPDFFPVYA

NGDQMGVDTSIIGSHVKHVLKNSLDITKHDIYTIGDYNIKKDAYTPDIGYMNDSSLRYDY

GKYYASKTFFDDAKKERILLGWANESSSVEDDIKKGWSGIHTIPRKIWLDKLGKQLIQWP

IANIEKLRQKPVNIYRKVLKGGSQIEVSGITAAQADVEISFKIKDLKNVEKFDASWTSPQ

LLCSKKGASVKGGLGPFGLLTLASKGLEEYTAVFFRIFKAYDNKFVVLMCSDQSRSSLNP

TNDKTTYGTFVDVNPIREGLSLRVLIDHSVVESFGAKGKNVITARVYPTLAINEKAHLYV

FNRGTSNVEITGLTAWSMKKANIA*

R-75 homolog

>Bevul.7G182600.1.p

MGGLYIYGLLHIFSLLAVSTTALPAREPKTTSSQPGQINSNSVLVALLDSHYTELAELVE

KALLLQKLEDVVGKQNITIFAPRNEALERDLDAEFKRFLLEPGNLRSLQTLILSHIVPVR

VNSDEWPTHENQPQKHITLSNDRVHLQTDKKSGQKTVDLMRVIRPNDVVRPDGVIHGIER

LLVPHSVLEDFNRRRSLRSISAVLPEGAPVVDPRTNKLIKKKKSVGPPPPPGAPPALPIY

AAMAPGPSLAPAPAPGPGGPHHHFNGEAQVKDFITTLTHYGGYNELADILVNLTSLATEM

GRLVSEGYLITVLAPNDEAMAKLTTDQLSEPGAPEQIMYYHMIPEYQTEESMYNAVRRFG

KIKYDTLRLPHKMTAEEADGSVKFGQGEGSAYLFDPDIYTDGRISVQGIDGVLFPIEEKD

HQISALQKSNRAKVVTKPRRGNFFDDLFAL*

R-76 homolog

>Bevul.6G025100.1.p

MAKSLNHLIVLCFLVVAPVCLSMKSQGGPLYPQFYGHSCPKVEEIVWSVVAKAVAKERRM

AASLLRLHFHDCFVKGCDGGVLLDSSGTLVSEKRSNPNRNSARGFEVIDEIKAAVEKECP

MTVSCADILAITARDATVLTGGPSWVVPLGRRDTLDASLSGSNYNIPAPNNTFQTILTKF

KLKGLDLVDLVALSGSHTIGDARCTSFRQRLYNQSGNGQPDNTLNQAYASVLRTRCPQSG

GDQTLFNLDYATPFKFDNSYYKNLLAYKGLLSSDQILVTKNQASMNLVKQYAENNALFFQ

HFAQSMVKMGNIAPLTGSRGEIRRVCRRVNP*

R-77 homolog

>Bevul.7G209100.1.p

MALISSLTLFSFLAIILTLAQAQSELILTVVNNCPFTIWPAVLPNHGFPILEKGGFPLSS

LTHRSFPVPNTHWAGRVWARTGCTYSNGKFSCATGDCGGRIECNGLGGQVPATLAQFSIH

HGNRDHSAYGVSLVDGFNLPMTITPHEGKGVCPVVGCKANLLQNCPKPLEFRGLHGNGPI

VGCKSGCLAFNTDEFCCRNQYNSAKTCHGNTYSQYFKHACPATFTYAHDTSLMHDCAAPR

ELKVIFCH*

R-78 homolog

>Bevul.5G123000.1.p

MAKSSADDQELRRACESAIEGTKQKIIMSIRVAKSRGVWGKSGKLGRQMAKPRVLAISIK

QKAQRTKAFLRVLKYSNGGILEPAKIYKLKHLSKVEVNSNDPSGCTFTLGFDNLRNQTVA

PPQWTMRNTDDRNRLLLCILNVCKDMLGRLPKVVGIDVVEMALWAKENTTTTATTNQVNS

EDGPDPTTITEKDMKVNVEKDLVSQAEEEDMEALLGTYVMGIGEAEAFSERLKRELQALE

AANVHAILETETLVEQVLRGLDNALNCVDDMDEWLGIFNVKLRHMREDIESIETRNNKLE

MQSVNNKALIEELDKLLERLHVPNEYASCLTGGSFDESNMIKNIEACEWLSGALHGLEVP

NLDTTFANMRAVREKKAELEKLKSTFVRRASEFLRNYFASLVDFMTSDKSYFSQRGQLKR

PDHADLRYKCRTYARLLQHLKCLDKICLSPLRKAYCSSLNSLLRREAREFANELRASTKA

SRNPTVWLDASAGAGQTVSNADTSAVSDAYAKMLTIFVPLLVDESSFFAHFMCFEVPALN

PPGGVPNGNKSASNVDDADDDDLGIMDIDDNDSKAGKNSAELAALNECLQELLDGIQEDF

YAVVDWAYKIDPLRCISMHGITERYLSGQKADAAGFVRLLLGDLESRISMQFSRFVDEAC

HQIERNERNVRQMGVLSYIPRFATLATRMEQYIQGQSRDLVDQAYTKFVSIMFVTLEKIA

QTDPKYADVFLLENYAAFQNSLYDLANVVPTLAKFYHQASEAYEQACTRHISMIIYYQFE

RLFQFARRIEDLMYTITPEEIPFQLGLSKMDLRKVLKGSLSGVDKSIQAMSKKLQKNLTS

EDLLPSLWDKCKKEFLDKYESFAQLVAKVYPTENIPSVAEMRDLLASL*

R-79 homolog

>Bevul.8G085700.1.p

MMVEDLGVEAREAAVREVAKLLPVPELLQSISSIKADYISRQQANDAQLSTMVAEQVEQA

QSGLKSLSLSERTVNQLRDNFVSIEKLCQECQTLIDNHDQIKLLSNVRNNMNKTLKDIEG

MMSISVEAAEARESLSDDKELINTYERLTALDGKRRFALAAVASHKEEVGRIREYFEDVD

RTWETFDKTLWGHVGNFHKLSKESPQTLVRALRVVEMQEILDHQLAEEAAEAQGDGAMAS

IGNARRTTKDGKRNNAMASPKRKIQGKGYKDKCYEQIRKTVEERFNRLLTELVYEDHKAA

LEEARTIGEELGDIYDYVAPCFPPRYEIFQLMVNLYTERFVQMLRLLSDKANELTNIEIL

KVTSWVVDYQQNLIDLGVDESLAQVCSESGAMDPLMNAYVERMQATTRKWYLNILENDKT

QPPKRTEEGKLYTPAAVDLFRILGEQVQIVRENSTDVMLYRIALAIIQVMNDFQAAERKR

LEEPASEIGLEPLCAMINNNLRCYDLAMELSNSTLEALPQNYAEQVNFEDTCKGFLEVAK

EGVHQTVTVIFEDPGVQELLVKLYQKEWCEGQVTEYLVATFGDYFTDIKMYIEERSFRRF

VEACLEETVVVYVDHLLIQKHHIKEETIERMRLDEEVLMDFFREYISVSKVENRVRILSD

LRELASAESLDTFTLVYTNILEHQPDCPHDVVEKLVSLREGIPKKDAKEVVQECKEIYEN

SLVGGNPPKAGFVFPRVKCLQASKGSIWRKLT*

R-80 homolog

>Bevul.1G149700.1.p

MGLFDGLPVPSDKAHLREDLARIDESWAAARFDSLPHVVRLLTAKDELQALKEQSEVIED

VVDEVVHAYHSGFNRAIQNYSQILRLFSESADSISVLKVDLAEAKKRLGARNKQLHQFWY

RSVTLRHIISLLDQIEGISKVPARMEKLIAEKQFYAAVQLHVQSTLMLEREGLQTVGALQ

DVKSELTKLRGVLFYKVLEDLHAHLYNKGDYSSVSTSLLERDDEVTMSATFSLSMSISQP

ASRRTRSPKTDNQVDEGSFDGHGEEDGLRGEEADGYVRTNGGDYGLKKGGIITNRLPPWL

SSATPDEFLEAVRKSDAPLHVKYLQTMVECMCKLGKVAAAGAIICQRLRSTIHEIISSKI

KAHAENMSSSRPGSGLTAYSSTLQTLKGQLETYQLSKQKRANGLSLSGTLLAVSPVTPVM

APMGTAQAAARELLNSILDAIIKIFENHVVVGELLEEKASQQVEINTPRSLVDVGSNSES

EVTGGYNIGFSLTVLQSECQQLICEILRATPEAASADAAVQTARLAGKVPSKEKRDGSDD

GLTFAFRFSDATMVIPNQVQSWNKKAQGLLQEGYGSASVLPEEGIYLAAAVYRPVLQFTD

KVASMLPEKYIQPGNDGLLAFVENFVKDHFLPTMFVDYRKSVQQAISSPAAFRPRAHAAA

SYTPSVEKGRPVLQGLLAIDLLAKEVLGWAQAMPKFAAEFVKYVQTFLERAYERCRTSYM

EAVLEKQSYMLIGRHDIESLMKLDPASSCLQNCLSDNPSDAYGIEVEKQMSELLLGLRPI

KQEYLIRDDNKLILLASLSDSLEYVADSIERLGETFSMACNQVEEKGRNQRTPRRSEVSS

SPSKDLASFADDYRKLAIDCLKVLRVEMQLETVFHMQEMTSREYLDDLDAEEPDDFIISL

TAQITRRDEAIASFVSGIKQNYIFGGICGVAANTSIKVLADMKSINLFGVQQICRNSIAL

EQALAAIPSIDSEAVQLRLDRVRTYFELLNMPFEALLAFITEHEDLFTPEEYANLLKIQV

PGREIPADALDRVSDILR*

R-81 homolog

>Bevul.5G012100.1.p

MKESTGNGPLILDIEDFKGDFSFDGLFGNLVNELLPSYQDDDTDAHHDGHGNIGANDVLP

NGTLRISSEATKVAQGLSMPLFPEVDSLLTLFKDSCKELIDLRQQVDGRLSSLRKEVSQQ

DAKHRKTLAELEKGVDGLFDSFARLDSRISSVGQTAAKIGDHLQSADAQRETASQTIDLI

KYLMEFNSTPGDLMELSPLFSDDSRVAEAASIAQKLRQFAEEDIGRQGIGVQSTTGNATA

SRGLEVAVANLQEYCNELENRLLQRFDTASQRRELSTMAECAKILSQFNRGSSAMQHYVA

TRPMFIDVEIMNADNRLVLGEQGSQANPSNVARGLSSLYKEIADTVRKESATIMAVFPSP

NEVMAILVQRVLEQRVTALLDKLLQKPSLLHPPPLEEGGLLVYLRMLAVAYEKTQELARD

LRAVGCGDLDVEGLTESLFSAHKEDYHEHEQASLRQLYQAKMEELRAESLAELGGSGSIG

RSKGASIASNHQQISVAVVTELVRWNEEAVSRCNIFSSQPSVLAANVRAVFTCVLDQVSQ

YLTEGLERAREGLTEAAALRERFVLGTNISRRVAAAAASAAEAAAAAGESSFRSFMVAVQ

RCASSVALVQQYFANSISRLLLPVDGAHAASCEEMATAMSKAESAAHKGLQQCIDTVMAE

VERLLSAEQKATDYRSPDDGIIPDHRATSACTRVVTYLSRVLESAFTALEGLNKQAFLTE

MGNRLHKLLLSHWQKFTFNPSGGLRLKRDITEYGEFVRSFNAPSIDEKFEVLGILANVFI

VAPESLSTLFEGSPSIRKDAQRFIQLRDDYKSAKLAAKLSSLWPSLG*

R-82 homolog

>Bevul.5G012100.1.p

MKESTGNGPLILDIEDFKGDFSFDGLFGNLVNELLPSYQDDDTDAHHDGHGNIGANDVLP

NGTLRISSEATKVAQGLSMPLFPEVDSLLTLFKDSCKELIDLRQQVDGRLSSLRKEVSQQ

DAKHRKTLAELEKGVDGLFDSFARLDSRISSVGQTAAKIGDHLQSADAQRETASQTIDLI

KYLMEFNSTPGDLMELSPLFSDDSRVAEAASIAQKLRQFAEEDIGRQGIGVQSTTGNATA

SRGLEVAVANLQEYCNELENRLLQRFDTASQRRELSTMAECAKILSQFNRGSSAMQHYVA

TRPMFIDVEIMNADNRLVLGEQGSQANPSNVARGLSSLYKEIADTVRKESATIMAVFPSP

NEVMAILVQRVLEQRVTALLDKLLQKPSLLHPPPLEEGGLLVYLRMLAVAYEKTQELARD

LRAVGCGDLDVEGLTESLFSAHKEDYHEHEQASLRQLYQAKMEELRAESLAELGGSGSIG

RSKGASIASNHQQISVAVVTELVRWNEEAVSRCNIFSSQPSVLAANVRAVFTCVLDQVSQ

YLTEGLERAREGLTEAAALRERFVLGTNISRRVAAAAASAAEAAAAAGESSFRSFMVAVQ

RCASSVALVQQYFANSISRLLLPVDGAHAASCEEMATAMSKAESAAHKGLQQCIDTVMAE

VERLLSAEQKATDYRSPDDGIIPDHRATSACTRVVTYLSRVLESAFTALEGLNKQAFLTE

MGNRLHKLLLSHWQKFTFNPSGGLRLKRDITEYGEFVRSFNAPSIDEKFEVLGILANVFI

VAPESLSTLFEGSPSIRKDAQRFIQLRDDYKSAKLAAKLSSLWPSLG*

R-83 homolog

>Bevul.3G213900.1.p

MNAKTKRRTVAENGDTGTGEDLVLATLIGNGEDLSPLVRHAFETGRPDPLLQQLRNLSKK

KEAEIEDLCRLHYEEFIIAVDELRGVLVDAEELKAELQTDNFKLQEVGASLLSKLDEILE

SYSIKKNISEAIKMSKMCFQLLELCSKCNAHVSEGKFYPALKTIELIEKDYLKNIPVRKL

RLVVEHRIPLIKLHIEKKVCNEVNEWLVHIRTLAKDIGQTSIAHTASVRQKDEDMKARQR

EVEENNRLQLPEAAYSLDVEEIDEDLSLKIDLTPIYRAYHIHKCLGIEERFREYYYNNRL

AQLNSDLQIFSSQPFLESHQTFLGQVAGYFIVEDRVLRTAGGLLEESQVETMWETAVSRI

TSVVGGQFSQMTNTSHFLLIKDYMTLFGATLRYHGYEVKSLLETINGTKDKYSKLLMEEC

RKQIFDIVANDSFEQMILKKESDYQGTVLSFQLQTSDIMPAFPYVAPFSSMVPEACRVVR

SFIKDFVNYLSSGGEANLFDGVKRCLDTLLIDVLNEVILDKISDTSIGVSKGMQIAANVA

FLEKACDFFVKTATHLCGVPTRSMDHPQSTLTAKVVLKTSRDAAYIALLSLVNNQLDEFM

SLPENINWIPEEILENGHEYMNEVIIYLDTLLSTAQQILPMDALYKVGSGALEHISNSIT

GAFLSESVKRFNANAVVAIDNDLKILENFADERFESTGLSEDYKDGHFRYYLIEARQLLN

LLLSSQPENFMNPVIRQKNYNALEIKKIAIICDKFKDTADSLFGSLSNRNAKTSSRKKSM

DVLKRRLRDFS*

R-84 homolog

>Bevul.7G101200.1.p

MQSTKTRRRIPPGDSEQSSNGDKQDQLLLSSAICNGEDLSPFVRKAFVAGKPDALLHLLK

YFSRSKESEIEEVCKAHYQDFILAVDDLRSLLSDVDDLKSSLSLSNSHLQSVAQPLLSSL

DAFIETRNVNANLNQCMDLVVLCVQIVELCARANSHLKNDQLYLALRCIDQLERCYLNKN

KVPSLTLGNMLEKHIPAVRVYVEKKTNLELSDWLVEIRAVSRNLGQFAIGQASSARQREE

ELRMKQRQAEEQSRLSLRDCVYALEEDDDEDDLDLGMVGSGNDDRGNGGSLGFDLTPLYR

AYHIHQSLGMEDRFKHYYFETRKLQLTSDFQVSSMTPFLESHQTFFAQIAGFFIVEDRVL

RTAGGLISKWEVENLWEMAISKMCSVLEDQFSRMQTANHLLLIKDYVSLLGVSLRRFGYP

VDALLDVLSKHRDKYHELLLSDCRKQITDAVSADKFEQMLMKKEYEYSMNVLAFQLQTSD

ITPAFPYVAPFSTTVPDCCRIVRSFVEDSVSFMSYGGQLDSYDVVKKYLDRLLSEVLDEV

LVKLIRTSVFGVSQAMQVAANMAVFERACDFFFKHAAQLSGIPLRMADRGKRQFPLKKAR

DAAEEMLAGMIKAKIDGFMSLIENVNWMGDEPLQGGNEYVNEVIIFLETLVSTAQQILPP

HVLNRVLQDVMSHISEKIVGALAGDIVKRFNMNAITGLDIDIQLLESFAENQGSGLSEAD

ANQLKLGLAESRQLVNLLLSNNPENFLNPVIREKTYNALDYRKVVTITEKLRDSSDRSFF

ATRGARPNPKRKSLDALIKRLREVS*

R-85 homolog

>Bevul.4G101900.1.p

MELPFSIIFSIVSLSFTLPLLNTLWFWRTTPDLLMSPFPFIRHWTLSHKIRVNFSLKFRL

IMFYLAYQQVLIFLAAIDSPFQAFRGASKILFELAGFKQICSISYLYVMCFTFYYIGYNK

YRGLSEEVVEMEHELSELRKHISAHGILIQDLESGVFCEIDEWIKAGNQEDVPESQISEF

NDLSSKDDFDVKKIFLEDIDILLAEHKVLDALEALNMEEKSSPELRSAGDMNVPYKSEFL

KRKASLEDQLVKFSAQPSVGDVELKQALSGLQKLGKGSMAHQILLKKYASRLQKRVEASV

PLCSLYLETYPATFSKIIFSFISLAAKESHSTFGDDPVYTNKVVQWAEEEIESFVRLVKE

HGPLSETTTALRAASVCVEASLRNCTILEVQGIKLSKLLMVLLKPYMEEVLEMNFRRAKR

VFLDITENDGCLLLSPRFFSPLSVFASSSDDVLISSGLKFISIIKDIVEQLTPLAILQFG

ANLLGRVVQLFDCYVDLLTKALPSPSEDDGITELKEALPFKAETDSQQIAVLGVANSVVD

DLLPMAVSTIWNVKMETHDPTIGSHESISPIGGHAIEFKDWRRQLQHSLDKLRDHFCRQY

VLTFIYSREGKARLEARIYVDGEGEDFLWSSDPLPSLPFQALFARLQQLATVAGDVLLGK

EKLQKALLARLTETIVMWLSAEQEFWSVFEDGSVPLKPFGLRQLILDMHFTVEIARFAGH

SSRQVLQLASGIIARAVKTFAARGIDPQSTLPEDEWFSEAAKAAINKLLLVASGSEASET

EEHAVLHQHIVSDSEDSISCASTADSFHSFVSAETDLDSPSHSFSEIVHLPMGLGS*

R-86 homolog

>Bevul.4G106500.1.p

MGLPIHPVIAGAASMDTLSSKAASMRESLQKSQTITDNIVSILGSFDHRLSALETAMRPT

QIRTHSIRRATENIDRTLKSAEVVLSQFDLSRQAEAKVLKGPHEDLESYLEAIDQLRSII

RYFGSCKGFRNNEGVVTHVNSLLAKAVLKLEEEFKQLLASYSKPVEPDRLFECLPNSMRP

SSGSHGDQADPGAKNHGDSKPSENAVYTPPALIPPRILPLLHNLAQQMVEAGNHQQVNKI

YRDTRASVLEQSLRKLGVEKLSKEDVQKMQWEVLEAKIGNWIHFMRIAVKLLFAGERKVC

DQMFEGIDSMRDQCFADVTASSVELLLSFGDAIAKSKRSPEKLFVLLDMYEIMRELHSEI

ELLFSGKVCAEMREAAFGLTKRLAQTAQETFGDFEEAVEKDATKTAVSDGTVHPLTSYVI

NYVKFLFDYQSTLKQLFQEFEQAEDSNSQLAKVTMRIMQALQTNLDGKSKQYKDPALTHL

FLMNNIHYIVRSVRRSEAKDLLGDDWVQRHRRVVQQHANQYKRIAWAKILQSLSTQGLTS

SGGGGSGVAVDGGNSSGVSRALIKDRLKTFNVQFEELHQRQSQWTVPDTELRESLRLAVA

EVLLPAYRSFVKRFGPLVESGKNPSKYIRYTPEDLERMLGEFFEGKTLNEPKR*

R-87 homolog

>Bevul.7G216200.1.p

MAENNINGEEKLIAVARQIAKSLGHSNSMTDDIINIFSTFDGRFSRDKLSPSSETITMLP

ESRIADKPSSAVVTLEDCLSSLDHQISQYLSFDRPLWFDSADSSAFLEILDELIATIRDF

APLARENKQLAVVLDRAEDLLQSAIFKLEEEFRGLIQRGADSFDATRDSSDSDSEGNYDD

DGGAIPVAYPVTDFNIVIDALPSSTINDLHEIAKRMVAVEYGRECSHVYSACRREFLEES

LSRLGLQKLSTDDVQKLSWPELEDEIERWCKSAVFSLRILFPSERRLCDRIFFGFPPTSD

FAFMEVCRGSAIQLLNFADAVAKSSRSPERLFKVLDIYETLRDLIPEFEILFSDQYCVFL

RNEALAIWKRVGDAIKGIFMELENLIRCDPVKDPVPGGRLHPITRYVMNYLRAACKSQQT

LEQVFEEERERGMSGISSLSVQMAWIMELLESNLEAKSKLYKDLALSSVFMMNNGRYIVQ

KVKDSELGLLLGEDWIRKHNVKVRQYNMNYQRSTWTKVIGVLKPENGGGSNVNGGPNSRT

LKERFRMFNTYFEEVLKVQSSWIVFDDELREQLKISVTQNLLPAYRNFIGRFQNSPEAGR

NPEKHIKYSVEDVEAQINNELFRGNNNGGRR*

R-88 homolog

>Bevul.7G167100.1.p

MESPPRSSGAGGEDGGIESAEKIILRWDSTVSEEARDKMIFSGDRHEVDRYLKAVDEIQR

SVHSLAMASPSSGGSGGGGDRNPNTIQIAMARLEDEFRNILLSHSSAVDADSLLVDTPRS

HSSSSRDHFDHFAAAADAAVEDEERGNIPRLDSGGSSSGGGGGGGGSSSSSRRSIRSTTS

IREVDLIPLDAIGDLRSIAERMIAAGYLRECIQVYGSVRKSSVDASFRNLGIEKLSIGDI

QRLEWDALENKIRRWIKAAKVCIRTLFASEKRLSEQIFDGLNAYSSSSLSGGGACCGSSP

PPSPCSYSEACFLETVKGPAMQLFNFAEAISISRRSPEKLFKILDLHDALAELLADIEVV

FESKTAESIRVQAAEILSRLAEAARGILSEFENAVLREPSRVPVPGGTIHPLTRYVMNYI

SLISDYKQTLFELIVSKPSSSGTRYSGELSVPDFDFSELEEERTTLALHLIWIIVILEFN

LEGKSQHYKDTSLAHLFMMNNVHYIVQKIKGSPELREMIGDEYLKKLTGKFRLAATSYQR

ATWVKVLNCLRDEGLHVSGSFSSGVSKSALRERFKSFNAVFEEVHRTQAMWFVPDTQLRE

ELRISIAEKLLPAYRSFLGRFRSHIESGRHPEQYIRYSWEDLDSAVLDLFEGNPVTQHMR

KRSQG*

R-89 homolog

>Bevul.7G167100.1.p

MESPPRSSGAGGEDGGIESAEKIILRWDSTVSEEARDKMIFSGDRHEVDRYLKAVDEIQR

SVHSLAMASPSSGGSGGGGDRNPNTIQIAMARLEDEFRNILLSHSSAVDADSLLVDTPRS

HSSSSRDHFDHFAAAADAAVEDEERGNIPRLDSGGSSSGGGGGGGGSSSSSRRSIRSTTS

IREVDLIPLDAIGDLRSIAERMIAAGYLRECIQVYGSVRKSSVDASFRNLGIEKLSIGDI

QRLEWDALENKIRRWIKAAKVCIRTLFASEKRLSEQIFDGLNAYSSSSLSGGGACCGSSP

PPSPCSYSEACFLETVKGPAMQLFNFAEAISISRRSPEKLFKILDLHDALAELLADIEVV

FESKTAESIRVQAAEILSRLAEAARGILSEFENAVLREPSRVPVPGGTIHPLTRYVMNYI

SLISDYKQTLFELIVSKPSSSGTRYSGELSVPDFDFSELEEERTTLALHLIWIIVILEFN

LEGKSQHYKDTSLAHLFMMNNVHYIVQKIKGSPELREMIGDEYLKKLTGKFRLAATSYQR

ATWVKVLNCLRDEGLHVSGSFSSGVSKSALRERFKSFNAVFEEVHRTQAMWFVPDTQLRE

ELRISIAEKLLPAYRSFLGRFRSHIESGRHPEQYIRYSWEDLDSAVLDLFEGNPVTQHMR

KRSQG*

R-90 homolog

>Bevul.2G074500.1.p

MGGYESEVPAEGDLIAAAQHIVAALGTKKMLSQDERKILVDLRTRLSTMTMVDDTKTNEP

PVNGVSETEQYLNLVSERIMNRVSDETMIWDLGPEDACEYLKAVEEARILTEKLEGLCLN

KDDEEVELLRKAHDVLQTAMARLEQEFGHIIVQHSQPFEPEHMSFRSSEGVGVVGSPCSL

EDDPLEDSLRRESVGKNLEDFIVDLVHPDVIPDLKCIANLMFISSYERECSQAYVTARKH

ALNRCLFALEVEPLSIDDVLKMEWGSLDSQIKRWVWAIRIFVRGYLANEKRLTDEIFGDF

GSLSSMCFTDSSKASVMQLLSFGDAVAVSPRQPEKLFRVLDMYEVLAQLHADINSMYTDQ

AIRDEFCEVLRRLGDAVRATFIEFKNAIATNASINPFAGGGTHHLTRYVMNYLKLLTDYR

DTLNQILEEAERVESLSPLADSVPFWDEMKQFDGTCNATPMAFHFQSITSALLANIDEKS

KLYKDTALQHFFLMNNIHYMAQKVKSCELRDIFGDDWIKRHNGKFQQQAMNYERATWSSI

LSLLKDDGSQNSGSNSSIKNILKERLKSFYAAFEEVYRNQTGWLIKDSQLQEDLRIKTSL

QVIQAYRTFIGRHSSEISDKYIKYTADDLENHILDLFSGSQRSLSNIYRK*

R-91 homolog

>Bevul.5G154500.1.p

MATTVEGEDRVLATAQHIVKSLRTSKDVTDDMLLILSTFDNRLSNISDLVSAPTSNINSL

SSSELSRLDFAEKLILRHDSNNNHFIDSPDESCEFLAAIDDVLDLIADLKLQQNPTFIDR

AESAVQSAMSKLEDELRSLLARSAVPIDADRLYGSIHRVTLSFASHDGDIIDGDFGNFNH

PYHERGGSLAGDVSVDLIYPDVVSDLREIADRMIRAGYEKECCQVYSSVRRDALEECLAN

LGVERLSIEDVQRIEWTALDEKMKKWVQAVKIFVRVLLTGEKRLCEIIFDGAQSELIREV

CFVETAKGCVLQLLNFGEAVAIGKRSPEKLFRILDMYEALDDTLRYLRALFCDDSGEFVC

AEARGVLDGLGEAARGTFIEFESAVKGEVSRKLVQGGEIHPLARYVMNYVKLLVDYSATL

NKLLEYDVVEGEGEGEGEADNSQGRSDGGEAREMSPLGKRLTSLISSLESNLEEKAKFYD

DSAMQYIFLLNNKLYILQKVKDSELGKLLGDDWVKRRRGIIRKYATNYLRASWSKVLACL

RDEGIGSGSNSAFKIALKERFKNFNTCFEDIYRTQTSWKVPDPQLRTELRISISEKVIPA

YRSFLGRFGPQLENTRHAGKYIKYTAEDLDGYLADLFEGTPGVLHHMRRKSS*

R-92 homolog

>Bevul.3G069900.1.p

MGSNNIERLFSARKSLNLSSEKSKTLGLALEKAGPRLEEINQRLPSLEAAVRPIRANEDA

LVSVVGHIDRAVGPAAAVLKVFDAVHGLEKSLLSDSRNDLPGYLAVLKRLEEALLFLGNN

CGLAIQWLEDIVEYLEDNVLADERYLSELKTSLKGLREFQNDGGNVSLDGGLLEAALDKL

ENEFRCLLTENSIPLPMSSVDDQACIAPSPLPVAVIQKLQAILGRLIANKRLDNCISIYV

EVRSSNVRASLQALNLDYLEISVSEFNDVQSIEGYIAQWGKHLEFAVKHLFEAEYKLCID

VFERIGLDVWMGCFAKIAAQAGILAFLQFGKTVTESKKDPIKLLKLLDIFASLNKLRLDF

NRLFGGAACAEIQNLTRDLIKRVIEGACEIFWELLVQVELQRQTPPPIDGSVPRLVSFVT

EYCNRLLGDDYKPLLTQVLVIQRSWKNEKFQERLLVNAILEILKAIELNLEAWSKMYEDT

ILSYLFLMNNHWHLYKHLKGTKIGSLLGDTWLKEHEQYKEYYAAIYLRETWGKLPTLLSR

EGLILFSGGRATARDLVKRRLKKFNETFDEIHKKQSGWVVSDKELREKTWQLIVQAIVPV

YRSYMQNYGPLVEQDSSSTKYAKYTAQSLEKMITSLFLPKPGRFNSFKGRQFSGKFNNVP

ADQHQPSPTGSK*

R-93 homolog

>Bevul.7G214100.1.p

MEDTMDSSMKIIASWDYETSDISPNTSLFNHHSRLEVLEFMRAVKDLHAAIQFFATTSAF

FSATSANSANHPPHKKIVSSQNLLRVAKKRLSSEFFSLLKANSKHHIDYYSVEDDNSHSS

SGDLSTLNRNNFENNNIFTVTNYNNNSSEDHSTTEESSAMADLKLITDCMMSAGFGKKCL

KIYKLTRRSFVDDVLLKLGVRRLKKFQVQKFDWETIDAKMNRWLKIVGTCVKVFGNEKNL

CEHLFSASTLSSVTETCFSEVCKEAALLLYRIPGKLAKHSKKSHDKILKFLNLYEGMSED

ISEVDRIFSSEVTSAVRLQLRSSHGRVSETVKLMVTEFEASIHKDASKGLVSGGAVHPLT

KHVINYLVNLSHNAILLDKILADYPVSLQSPLPESYFEGETLSSPVALQFAWLILVLLGK

LDTKSELYKDAALSYLFLANNLQYVINQVKSTKLYNVLGEKWVTKHTSKVVQYAANYERM

GWGRVISSLPKDITIEISLDKVKDSFKRFNIGFEEEYRKQVDWIVQDSKLRDDMKASIVR

KVDLRYRKMYDKYKVVLAKTRERGKIDSVIKFTPEDLENYLSDLLDGHEEDDDVSGSRGS

VETTSYDGKENPVLQRRSRRLLLLVRQHHHQ*

R-94 homolog

>Bevul.4G216200.1.p

MRASSENHSAAMASGAGFREAETLFRAKPVSEIRNVEMNTRKQIEDKKEELRQLIGNRYR

DLIDSADSILLMKSSCESISSNISSISDSIHSLSSAVSPKLSSNPNPNRFKIYGIASRVK

YLVDTPENIWGCLDEFMFLESSARYVRAKLVYQGLNLVGHDGGMILNKNFPLLNHQWQIV

ESFKAQISQRSRERLLVMLDKEESDVWKLPIGAFADALAGVAIIDELDPELAFGLFLDSR

KSWILQKLSNFDDNAVVPVLCEVTRIIQVTIAHVGEMFLQVLNDMPLFYKVILESPPAAQ

LFGGILNPDEEVRLWSEFRDKVESIMVILDREFIAKACSRWLRECGREIVKKIDGRCLID

VIPNGAELASAEKLIRETMESKDVFEGSLEWLKSVFGSDIELPWSRTRELVLEENVDLWD

EIFEDAFVSRMKVIIESAFRNLTGAVNVSDSIRAVGGNFDDKSDFGAYLNRASADGGVWF

IEPNVRKGGIVPGIKALHEEFDFGSCLSAYFGPEASQIRDAVDSCCYNVLEDLLYFLESP

KAPLRLKDLAPYLQNKCYESLSTILTDLTNELENLRAALDNVKKGGKFPPPAAIVERSLF

IGRLLFAFRNHSKHIPVVLGSPRVWVNEIVASGSETLPSSSRYSRFFVDSSTPDSPKPLL

NNSKRQMSLAANALFGVNDTTSPKLGELTSSMRDLSIAAHGLWISWVSDELANVLSEDLR

VDDALSAANPLRGWEQVVVKQDQSSESDSEMKILLPSMPSLYICSFLFRACEEAHRIGGH

VLDKTILQRFAKRLLEKVIGIYDDLDTSGVQTSEKGILQVLLDLRFAADVLSGYDSTVSE

GLSKSPSTKFNYRRKQDRSTTKSATRERIDDLINRFSQRLDPIDWQTFEPYLWENEKQSY

LRHAILFGFFVQLNRMYTDTVQKLPTNSESNIMRCSTVPRFKYLPISAPALSSRAVAKAP

IRTSSDDVSSRSPWKAYTNGEFSPKASLDDNSSFGVAAPFLKSFMQVGSRFGEGTFKLGS

MLTDTRFKDKSAASMSFGDILPVQAAGLLSSFTATRSDS*

R-95 homolog

>Bevul.5G153100.1.p

MASADLAQPSPPPRSHTDVFGDPFEDSSPLWFKKDLFLSPKFDSETYISDLRTFVPFDTL

RSQLHSHLSSLKHQLVDLINRDYNDFVNLSTNLVDVDSSVARMRAPLSDLREKISAFRDC

VQDSLVSLQNGLNQRAQAANAREVLELLLDTFHVVSKVEKLIKELPSVPADWSNGDVNSS

EKSHLSNGLSLQQSENESNVRETQSMLLERISSEMNRLKFYMAHAKDLPFIQNIEKRFQS

ASLLLDSSLGHCFVDGLEHRDENAIYNCLRAYAAIDNTRNAEEIFCTTVVAPLIEKIIPH

ASSGLISVASEDELEADFREMKQCIEKDCTFLLRISSRENSGFHVFNFLANSILKEVLSA

IQKGKPGAFSPGRPKEFLKNYRSSLDFLSHLEGYCTSRSAVSKLRAEPVYVDFMKQWNLG

VYFSLRFQEIAGALDSALLAPGLVGQNSRHVDGNLPALTLKQSIVLLESLRSCWSEDVLV

LSGSDKFLRLSLQLLSRYSSWLSYGLNARKSGHTDAKPGSEWAISAAPEDLIYVIHDVKL

LVNEVCGDYVGHVMEVLSSFPKEVLDNVKQSVLQSGESLERTTLPVIKTSIIDALVQKSV

EDLKQLKGISATYRMTNKPLPVRHCPYVSAILHPLKAFLEGETAIYLDDATKKELLRGTA

LEITKSYNAMASELVNVARKTESSLQRIRQGAQRRAGASSDVSDHNVSNTDKICMQLFLD

IQEYGRNLAALGVDAAEFEDYRSLWQCVAPADKQSTIVF*

R-96 homolog

>Bevul.8G148500.1.p

MMEMDRRRRLHQMVGPKNTSSPSVPKSGAISKGYNFASTWEQNAPLTEQQQAAIVALSHA

VAERPFPPNLAQEGTPGQEHGLTIDTQDSVADNSAAIEAVLVNTNQFYKWFTDLEAAMKS

ETEEKYRHYVDTLIQRIQTCDGILRQVDDTLELFNELQFQHQAVANKTKTLHDACDRLLL

EKQKLIEFADALRSKLSYFDELENISTSFYSQSMNVGNQDFLPLLKRLDDCISYVESNPQ

YAESSVYLVKFRQLQSRALNTIRSHVQSVLKNASSQVHGAIRNIGGNKPAVSEGMEASII

YVRFKAASSELKLVLEEIESRSSRKEYSQLLVECHKLYCEQRLSLLKGIVQQRISDFSKK

EALPSLTRSGCAYLAQVYQLEHQLFSHFFPASAEDVSSLAPLIDPLCTYLYDTLRPKFIH

ETNIDFLCELVDVLKVEVLGEQLNRRSESLAGLRPTFERILADVHERLTFRARTHIRDEI

ANYLPVNEDLDYPAKLEQSPEVEEGAQTSEENQDVFRTWYPPLEKTLSLLSKLYRCLEPG

VFTGLAQEAVEVCSLSIQKASKLISQRSSPMDGQLFLIKHLLILREQIAPFDIEFSVTHK

ELDFSHLLDHLRRLLRGQASLFDWSRSSSIARTFSPRVLESQVDAKKELEKILKSTCEEF

IMSVTKLIVDPLLSFVAKVTAVKISLSSGSQNQKIETVMAKPLKEQAFATPEKVSELVQK

VSAMIQQELPRVIQKMKLYLLNPSTRTILFKPIKTNILEAHTQVQSLLKSEYSPEEIESI

KMVSTQDLQAELDSLL*

R-97 homolog

>Bevul.7G052600.16.p

MASSPPSPNTHQHPQQQFSIKFGTQESLSQIKSLTDVNTITRLLHECIAYQRSLDLDLES

LLSQRSSLDNNLFSLLKSAQILSFVRSDADQVLSSVRSTSILAENVSAKVRQLDLAQSRV

STTLLRLDAISQKSACIDAVKSALGCDDYESAAIHVKKFLEIDGEFPDSSGDAQREEMIA

YKKQLEGIVKKKLLAAIDHRDHDSVVKFIRLYPLFENNDEGLKMYVGYLKKVISLRSRTQ

FEQLVELMENSYSSSSSTSNSQVNFVACLTNLFTDIVLAIEENDEVLRSLCGEDGVVYAI

LGLQEECDARGSLIMNKFMEYRKLMNLTEEINSYSKNLLSVGSVEGPDPREVELYLEEIL

SLTRLGEDYTVYMLSKIKGLSSINPELVLKVTEAFEKGGFSKGLFEVTKFYVVLEVFFMV

ENVKKAIMIDEHVSDSLTTSMVDDVFYVLQSCCRRAISTSKSESVVSVLYSAANLLSNEY

LEALQQKMREPNLGAKLFLGGVGVQKTGTEIATALNNVDVSSEYILKLRHEIEEQCAEVF

PASSDREKVKFCLPMLVETSNSFKQALNAGMEQLVATVTPRIRSFLDSVGTISYELSEAE

YADNEVNDPWVQRLLHAVESNAAWLQPVMTANNYDTFVHLIIDFITKRLEVIMMQKRFSQ

LGGLQLDRDTRALVSHFSSMTQRTVRDKFARLTQMATILNLEKVSEILDFWGENSGPMTW

RLTPAEVRRVLGLRVDFKPEAIAALKL*

R-98 homolog

>Bevul.9G018900.1.p

MHTTTTTTTMSSPLSPSSSPFKHTHPSSLSSIDNNNNDPLSSFSSDPIFSLFLSPSFSST

HFSSQALSSGSAASTAERLQDAIRLLETQLRSQVLTHHDHLFSQLSSLNDADSALSLVRS

SVFSLQSSVRRIRSQITDPNNLIKSQTTQLTNLHSTSHLLQSSVKLLRLINKLRSLMCVD

DNNNTGEELLDILKAAQLHCEIMWISNSNDLSGIEAVDEELRWVSEAGARLRSEGMKVLE

RGLEGLNQAEVGSGLQVFYNLGELRGTVDGLVVKYKTMGVKSIVSALDMKAISAAVSAAG

FGGPGGIQRSGTPQIGGSGKAKDALWQRMSGCMDQLHSIMVSVWLLQRVLSKKRDPFTHV

LLLDEVIQEGDSILTDRVWEAFVKSFASQMKSAFTASSFVKEIFTLGYPKLYSMIENVLE

RVSRETDVKGVLPAISSEGKEQMRAAIEIFRTAFLALCLSRLSDLVNSILPVSNRGSVPS

KEHISRIILRIQEEIEAVQFDGHLTLLVLREVGKVLLLLAERAEYQISTGPEARQITGPA

TPAQLKNFMLCQHLQEIHTRISALLSGLSLEASEVLSPSLGVVYGVACDSVESLFQAMLD

RLQSCILQIHEHNFGVIGMDAAMDNNASPYMEELQKCILHFRSEFLSRLLPSSKTAAATG

TESISTKLARGMASRVLMFFIRHASLVRPLSESGKLRMARDMAELELAVGQNLFPVEQLG

APYRALRAFRPVIFLDTSQLEASPLLQDLRPSTILHHLYSRGPEELQSPMQRNKLTPLQY

SLWLDSQGEDQIWKGIKATLDDYATLVRSRGDKEFDPVYPIMLRIGSSLSENSSQSERT*

R-99 homolog

>Bevul.5G167800.1.p

MGTATLAPGLSRKLKKVLESRIDSPELLSSLSTLSTFYTDNSSHARRNLRSTIEKRSLSI

NHDFLTASDAAQQALDQVEEEVNALAECCDRIAKALSSCSATTGDIIGTTERLKLELDAT

TQRQEIVSCFLRDYQLSNEEINALKEEELNENFFKALSHVKEIHANCKILLKTHHQRAGL

ELMDMMAMYQEGAYERLCRWVQAECRRLGDTDNPEVSELLRTAVRCLKERPVLFKYCAEE

VANMRHNALFRRFISALTRGGPGGLPRPIEVHAHDPLRYVGDMLAWLHQALASERELVHA

LLDPDADMDSGPTARQLSNNSESERGKIEADLTFVLDRIFEGVCRPFKVRVEQVLQSQPS

LLVSYKLSNQLEFYSYTISDLIGSETVLCQTLWTLKGAAQKTFFDILKSRGEKLLRYPPV

VAVDLSPPPAVREGVSVLLEIMETYDGMMIPLSKKPQFDPVISALLDPIIQMCEQAAEAH

KSKGGGYSSRRNRLSSDSNQLNKSSVDAILSNNDTLSAPQSAETPSKIFLINCLCTIQQP

LIGHEAAATYAKSIGDMIENHMRILVDKEVETILRRCNLLSKMANFRKTFIEAREEALAG

PLAEVEETSPASLSESLNIFFGLILGTGNSLPEFEQLQVPKLRSEACIQLAKSLADSYEL

VYNAIMDPKNSYPDPKSLARHPPDQIRTILGI*

R-100 homolog

>Bevul.2G248500.1.p

MMVDLGAFSDEKFDAKKWINMAVKSRHPQDALEKHLVDLEMKLQLMSEEISASLEEQSAA

AILRVPRATRDVIRIRDDAVSLRSSISSILGKIKQAEGSAADSIAALAKVDTVKFRMEAA

YKTLQDAAGLTQLSSTVEDVFASGDLPRAAETLANMRHCLSAVGEVAEFANIRKQLEVLE

DRLEAMVQPRLTDALSNRKVEVAQEMRGILVRIGRFQSLEVYYTKVHLKSIKQLWDVFES

RQRSNKLATEKNDGERLASLNEFQSNHSALSLLSWLPSFYDELLFYLEQEWKWCTIAFPD

DYKTLIPKLVIETMLAIKPSFISRINLATGDAVPETKALAKGILDLLSGDMPKGIKIDSK

HLDAMIELHNVTCTFARNIQHLCSESEMQVLLDTMKAVYSPFETFKQKYGKMERAILASE

IGKLDLRGAVTRGVGAQGIELSETVRRMEESIPQVILLLEAAVERCINFTGGSEADELIA

SLDDIMLQYISMLNETLKSLRAVCGVDNTFDSAKRESGLDKKEGAHNPRKPDLISNEEEW

SIVQGALQILTVADCLTSRSSVFEASLRANLSGLSTSLVVSVFGVDQNQRKLSIDDENAN

AKLDVKAALDMATVRLFDAPEKARKLSYLLEQSKDPRFHALPLASQRVAAFAETVNELVY

DVLISKVRQRLSDLSRMPIWSSAEETSAFHLPSFSAYPQSYVTSVGEYLLTLPQQLEPLA

EGISSDDANTDEAQFFATEWMFKVAEGATAIYIEQLRGIQYITDRGAQQLSVDIEYLANV

LSALSVPIPPILSTFQTCLSTPRDQLRDLIKSDSGTQLDLSTANLICKMRRLNLDQ*

R-101 homolog

>Bevul.8G123100.1.p

MDSLSSYLEDNNNGEGIAGLIPLASVSQQPYVSELLSFTLDRLHKEPELLRVDAERIRRQ

MQEVAVANYRSFISAADALVSIREEVSSIDNHLESLIAEVPKLTSGCSEFIESAEQILEK

RKMNQTLLANHSTLLDLLEIPQLMDTCVRNGNYDEALDLEAFVSKLCTMHPKIPVIQALS

AEVKQTTQSLLSQLLQKLRSNIQLPECLRIIGYLRRIGVFSEYEMRLQFLRCREAWLTGI

LDDLDQRNPYEYLKGMVNCHRMHLFDVVNQYRAIFADDTSGSEENYDGGLLFSWAMHQIT

SHLRMLKVMLPKITEGGSLSNILDQCMYCSMGLGWVGLDFRGLLPSLFEEAVHNLFVKNI

TTAVQNFQLVLDSHRWVPLPAVGFSGSTAGEESQEDVTPPSYLMEHPPLAVFINGVSAAM

NELRPCAPISLKHILAQELVKGLRAVHNSLLRYNEARMLRENESTLFFSLCRAFMEVAFP

HCATCFGRCYPGGTGIIMDAKSSFDGLTRLLSVSSSREIPRRTQSLDIQSISTNGSSSTI

ENGAITESEHEGATDVVEEGVKSEDVKAEDKQGDS*

R-102 homolog

>Bevul.5G184400.1.p

MENDEVGPSNGKSNDQFIDRSKVRILLCDNDSKSSQEVFSLLCKCSYQVTSVRSARQVVD

ALNAEGSDIDLILSEVDLPTKKGFKLLKYIMRDKELQRIPVIMMSTQDEVSLVVKCLRLG

AADFLVKPLRTNELLNLWTHMWRRRRMLGLAEKDIVNYDFDLAQSDPSDANTNSTTLFSD

DTDDKSRRTVNPEMSITVQQEEECNKFTSEAVPRYSLDCQPAVPALSDRRTGQFAPFPKK

SELKIGECSAFFTYVKSGSIKSNCQAVIDVEGYAAQPSRPDDVSDTWNEHLNNGNMRYEN

GDTWENNSHGEDFPSSTSVPDSTSVERSCTPPVAVEFMLNKSSDEGLSEVHVQPKNGTHF

DVSTLATHAAYPYFMAGVMNQFMMPSSTHVHQKDLQNHGTSTVMSHYNHLPHCPSHLPGM

APFPYYPVGVCLPQGQGQSPANHQLTSFGNSSSSEAKLSKVDRREAALIKFRQKRKERCF

DKKIRYVNRKKLAERRPRVRGQFVRKLNGVTVDLNGDPSADIDEEDEDDRNSSPEDDTS*

R-103 homolog

>Bevul.1G069100.1.p

MERPSERWIDGLQFSSLFWPPPQETQQRKAQTTAYVEYFGQFISEQFPEDLAELIRSRYP

FDEKRLFDDVLATFVLHHPEHGHAVVLPIISIIIDGTLVYDKERPPFASFISLFCPNDEN

EYSEQWALACGEILRILTHYNRPIYKHERQKSPENCTSCKDYATCSNASDAKSSHSSSVQ

SERRTSRPLSPWITDILLAAPLGIKSDYFRWCGGVMGRYAAGELKPPMIASSRGNGKHPQ

LMPSTPRWAVANGAGVILSVCDEEVARYETASLTAVAVPALLLPPPTTSLDEHLVAGLPA

LEPYARLFHRYYAIATPSATQRLLLGLLEAPPSWAPDALDAAVQLVELLRAAEDYATGMR

LPRNWMHLHFLRAIGIAMSMREGIAADAAAALLFRILSQPALLFPPLRQVEGVDVQHEPL

GGYISCYRKQIEMPSAEATIEATAQGIASMLCAHGPEVEWRICTIWEAAYGLIPLSSSAV

DLPEIIVATPLQPPLLSWNLYIPLLKVLEYLPRGSPSEACLMKIFVATVESVLQRTFPLE

SSMEENRRSRYSSEAGAASKNLAVAELRTMVHSLFLESCASEELASRLLFVVLTVCVSHE

AHTNGKRSRVEDSYSDRAAKHSKSTQSSPSSSTKSKKQKESRSKKPKKQGPVVAFDSYVL

AAVCALACELQLFPLIAGISNPSSSKDSVEIAKPVKLNGSSHEFKDGIDSAIRHTRRILA

ILEALFSLKPSTIGTSWGYSSNEIVAAAMVAAHISELFRRSKACMNALSVLMRCKWDNEI

HSRASSLYNLIDIHRKAVASIANKAEPLEAHLSQTPVWRDSSIVTNGRKHNDFAGTVCFL

PEVPSTLTCEDPAHSKNSLNCGKAVHTNNDTGNTAGKSVASFQFDASDLAQFLTMDRHIG

FNCSVQIFLQSVLEEKQELCFSVVSLLWQKLIASPETQPSAESTSAQQGWRQVVDALCNV

VSASPAKAAAGIVLQAERELQPWIAKDDDQGQKMWKINQRIVKLMVELMRNYDTPESLVI

LASASDLLLRATDGMLVDGEACTLPQLELLEATARAVQPVLKWGESGLAVADGLSNLLKC

RLPATITCLSHPSAHVRALSTSVLRDIQQNGSIKFSFKQESRNGTHKTAFEYLHIGIIDW

HTDIEKCLTWEAHSRLTRGKTIEYLDMAAKELGCAITI*

R-104 homolog

>Bevul.8G214200.1.p

MVAATDDSNSNSNNMRGLLSGWGLSLTGRSCDSCHAAAALLYCKADAAFLCGPCDTKIHL

PTSYPRHERVWVCEVCEQAPAVVTCKADAAALCSSCDQDIHSVNPLASRHERVPIIPFLD

TTTAPSSAASAFLVPDHLPASIEDADAWLIPNPSTTLLYNDSDFLNFGYGFESPTQFQQQ

DVKPIVHQQNGAYAFTDSVVPTQTQTQTQSPSQSQSQGNNFNNVTTSLFENCFNIDFTQP

NKLNNTNNKNKNNHLFSSSFNYSANAHQSVSSSDIGVVPDGNTNSMSSEVSYTFVRSMSN

DVGSSIGAPVMDREARVLRYREKRKNRRFEKTIRYASRKAYAETRPRVKGRFAKRTDTKE

TSEIDFTLDDGYGVVPAF*

R-105 homolog

>Bevul.1G201000.1.p

MDNYSSGEEPIVKTRKPYTITKQRERWTEEEHNRFLEALKLYGRAWQRIEEHIGTKTAVQ

IRSHAQKFFSKLEKEALVKGVPIAQAIDIEIPPPRPKRKPSNPYPRKTGSISSPNTQVST

KDGKQIPHTHATEQVVDLEKEQLLEKSGCDDGDSNPDETDGGDKCTEAFTLFKEKPSALG

VPSSEFPSTTVGSARKLSNFKEFMPMKKPVNDDTGGFDAADVHNRNQKPEKPDVVEMVQN

DSENEKNAAKTFPKHAPVQILDGSLVSCSQGLSSDVSYQEPVFHQMGIPSLPALFSNPTV

SAAVESQTSTSRSTNHQMFPSFHPPFSPYPNTQEDYSSFLQMSSTFSSMIISALLQNPGA

HAAATFAASFWPCGNLENSANSPAVSSGGLPPRPMNPAPSVAAIAAATVAAATAWWAAHG

LLPLCAPLHAGYSFPPASGTTPLANVSQTQVANEEKEEKNFQRHGSQVQQPNQELSEALH

QEHLASKSSAASSSDSGNTSSAKMDTGVAANNNENKIPASSELKDTSKGKSKKQVDRSSC

GSNTPSGSDIETDALGKNDKVEEEAKEPDINRPTSEPNSRRNRVVCNIYDSWKEVSEGGR

LAFQALFSREVLPQSFSPPQNGNVEMLDKKLCIEGNRQIVERSNDVSQLDLNSNTWESCS

VNQGIEKNDTRQEDSCKNGLLIFSYEQGKLKTNRTGFKPYKRCSMEARESRVMNSSAPEQ

EKCSKRLRLEGEASI*

R-106 homolog

>Bevul.3G234100.1.p

MDVLNKQSCSIYSNFQHTNMARSNVPEFGNWKTEENVPYTTYFENARRVSFSEKVNPNSS

PDDDSTLASKLEGRKRFEAMRAKHDNMVSQEDGELQKSSDSPSRPDGGHKTSTNSTHSRP

GGVSSDTPKRAALANKVGFRSADHSPLHPHYQAKISGAKGNAASSPSWEKKGSSEGSHGL

APSTPGRSRLKSVPRGNETPDRGASVPKFGDWDETDPSSADGFSHIFNKVKEERHGGVGN

APGTATRSKDSDGQKLNRSKYSKGCGCFPW*

R-107 homolog

>Bevul.6G143500.1.p

MPFPSSSITRKSEKRPFLRKCHSTTTPYQSHHQLHLQSRSISMPSSDPNLSSASPVSLSS

TFFKRLLVSANWVFFYLLSFICFSSVAYTVSLWKRLTALQGEILELRRFCKGNDVIHNSV

IEISQSNDAKSSSDIINVSGKTFALYIVIFTILMPFLVYKYLDYLPRLLKSLSKRTENYK

EEVPPKKIFAYMVDVCFSFYPYAKLLALLFATIFLIGFGGLALYAVSDNSLAEALWLSWT

FVADSGNHADRVGVGPRIVSVSISAGGMLIFAMMLGLISDAISEKVDSLRKGKSEVIESN

HVLILGWSDKLGSLLKQLAIANKSLGGGVVVVLAERDKEEMEMDIGKLEFDFMGTSVICR

SGSPLILADLKKVSVSKARAIIVLASDENSDQSDARALRVVLSLTGVKEGVRGHVVVELS

DLDNEPLVKLVGSELVETVVAHDVIGRLMIQCALQPGLAQIWENILGFENAEFYIKRWPQ

LNGMCFEDVLLSFPDAVPCGVKVAAKSGKIILNPEDDYIIKEGDEILVIAEDDDTYAPGT

LPKVRRGLCPMTHDPPRYPEKILFCGWRRDIEDMIMVLEALLARGSELWMFNEVPEKERE

RKLTDGGLDIPSLRNIKLVHRVGNAVIRRHVEALPLEAFDSILILADESLEDSVVHSDSR

CLATLLLIRDIQSKRLPCRDTKSSNLQYPAFCHSSWIREMQKASDKSIVISEILDSRTRN

LVSVSRISDYVLSNELVSMALAMVAEDKQINRVLEELFAEKGNEMCIRPAEFYLHDQEEL

CFFDVMIRGRQRQEIVIGYRLADSDHDVINPSKKAEPRRWSVGDIFVVISRS*

R-108 homolog

>Bevul.6G169200.1.p

MGSYCQVLVLLLLSLPCILSQVTEFISIDCGGGSDYSDKRTGLAWISDSGIMSHGEVVDV

QNTNDDWEPYKKRRDFPTDDKKYCYTLSTKERRRYLVRATFLYGSTGNADTYPSFQLYLD

ATKWATVTVFDGARTYVREMIIRAPSSSIDVCLCCATTGSPFISTLELRPLNLSMYATDY

EDKFYLKVAARVNFGAPNEKPIRYPDDPYDRIWESDLIKRPNFLVGIAPGTERINTTKNI

EVNNREYPPVKVMQTAVVGTEGNLSYQLTLEDFPGNARAFAYFAEIENLRLNETRKFKME

HPYVPDYSNAVVNIAENANGTNKLYEPSYMNVTLDFVLSFSFVKTPDSTKGPVLNAIEIS

KYVKIGSKTDAQDVIALNAVRAMLPASLGKQDEGDPCSPTHWEWVICSSTAPPRILEIHL

SQRNLTGNIPPEIENMDALTDLWLDGNSLTGRIPDISNLINLKIVHIENNGLSGPLPSYL

GSLPSLQELYIQNNFFNGKIPPALLGKKIVFNYDGNPRLTKGTRLGSVKILGISVAALGA

LVAVILVIVLLLRNFRRKASREKIDYKGSSLHTSTKPPKGHTLKRGTHLMEDGVACCIKL

SEIEDATMKFSKNIGKGSFGVVYYGRMKDGKEIAVKTIGDPTSQGTKQFLTEVSLLSRIH

HRNLVPLIGYCEEANHRILVYEYMHNGTLRDHIHDPIKQRQLDWLTRLRIAEDAAKGLEY

LHTGCSPSIIHRDVKTSNILLDINMRAKVADFGLSRQAEEDLTHISSVAKGTLGYLDPEY

YANQQLTEKSDVYSFGIVLLELISGRKPITEEYGMDWNIVHWARSLIRKGDVGSMMDPLL

VDSVKLESVWRVAEVAIQCVEQHGISRPKMQEIILAIQDAVILEKGTENNQNFSSGSGKT

QSSRKTLLTSFLEIESPDLSNARLTPSAR*

R-109 homolog

>Bevul.5G113200.1.p

MGNNCISRLGKVGFWFKPPKGVIGESESKGKEKSSDVQFKAPEVVKIGSKLGRQESRLSR

QESRLGRQESRVNRQESQLGKQENRLGRQESRLGRQESRLGRQESRLTRQESRTISVNVN

VGDQEKNKKQQQPPVNNNNNNVKRVSSAGLQVHSVLRRKTGNLKEFYTLGKKLGHGQFGT

TFLCVEKTSGNEFACKSIAKRKLVNKEDVDDVRREIQIMHHLKGHPNVITIKDAYEDGAA

VHVVMELCAGGELFDRIVKRGHYTERKAAYLARIIVSVVEACHSLGVMHRDLKPENFLFV

NEKEESPLKTIDFGLSIFFKPGDVFNDVVGSPYYVAPEVLRKRYGPEADVWSAGVILYIL

LSGVPPFWAETEEEIFEEVLHGDLDFDSDPWPNISEGAKDLVRRMLVRDPKKRLTAHEVL

CHPWIMADGLAPDKPLDHSVISRLTQFCAMNKLKKMALRVIAERLSEEEIAGLKEMFKMI

DTDNSGQITFEELKNGLKRFGANLEESEIHYLMQSADVDNSGTIDYGEFIAAMLHLHRVD

KEDHLCAAFAYFDKDGSGYITKDELQQACREFGMEEIHVEEIIHEADQDKDGRIDYNEFV

AMMQRGNVDLGRRQKPSSAAAKEARQ*

R-110 homolog

>Bevul.3G186700.1.p

MYSHHHHQASLISISLFLLLLSSTLSAELCHPQDKKTLFNIKKAFNNAYIFASWTHNTDC

CEWYLVECDETTHRITSLIVSKDDKVAGSIPDAVGDLPYLESLTFHKLPKLTGHIPQAIS

KLKNLKSLTLSHNNLTGSVPDFLSQLTKLTYINFAVNKLTGPIPPSLSSLKELGGIFLER

NQLTGHIPESFGKFKQDDFYLHLAKNKLSGPLPKSFGAPNFASLDLSRNSFTGDASMLFG

ENNDHLQTINLSRNKFSFDLSKVVFTRSLFHLELSHNMIYGSLPPVLAKLPNLQQFNVSY

NRLCGRIPRGGELNRYNKYDYAHNKCLCDSPLPPCK*

R-111 homolog

>Bevul.9G226600.1.p

MGGGFRVLHLVRPFLSFLPEVQSADRKVPFREKVIYTVISLFIFLVCSQLPLYGIHSTTG

ADPFYWMRVILASNRGTVMELGITPIVTSGLVMQLLAGSKIIEVDNNVREDRALLNGAQK

LLGILIAVGEAVAYVLSGMYGSVGQLGVGNAILIILQLCFAGIIVICLDELLQKGYGLGS

GISLFIATNICENIIWKAFSPTTINAGRGAEFEGAVIALFHLLITRTDKVRALREAFYRQ

NLPNVTNLLATVLIFLIVIYFQGFRVVLPVRSKNARGQQGSYPIKLFYTSNMPIILQSAL

VSNLYFISQLLYRKYSGNFFVNLLGKWKESEYGSQSIPVGGLAYYVTAPASLADMAAHPF

HALFYIIFMLSACALFSKTWIEVSGSSARDVAKQLKEQGMVMPGHRDSNLQKELNRYIPT

AAAFGGICIGALTVLADFMGAIGSGTGILLAVTIIYQYFETFEKERASELGFLGL*

R-112 homolog

>Bevul.1G137600.1.p

MATGTAPPRGSAAAAASLRRRRPAAGGAAGGGASGTMLQFYTDDAPGLKISPNVVLMMSI

GFIAFVAVLHVVGKLWLVRKD*

R-113 homolog

>Bevul.5G216000.1.p

MDVLDSVFDPLRDFSKDSVRLVKRCHKPDRKEFSKVAFRTAVGFVVMGFVGFFVKLIFIP

INNIIVGST*

R-114 homolog

>Bevul.1G018600.1.p

MEQGVCTVWVPLCLILLFSYFRYASANAEGDALNALKTSLADPNGVLQSWDPTLVNPCTWFHVTCNSENSVTRVDLGNANLTGELVTQLGQLPNLQYLELYSNNISGPVPYELGNLTNLVSLDLYLNNLSGPIPDTLGKLQRLRFLRLNNNTLSGQIPRTLTNITTLQVLDLSFNDLSGTVPTNGSFSMFTPISFNGNKNLVMPPTPPSSPVPSAPTSNSGANSATGAIAGGVAAGAALLFAAPAIALAWWRRRKPQDHFFDVPAEEDPEVHLGQLKRFSLRELQVASDNFSHKNILGRGGFGKVYKGRLADGSLVAVKRLKEERTQGGELQFQTEVEMISMAVHRNLLRLRGFCMTPTERLLVYPYMANGSVASCLRERPETDPPLSWEIRKRISLGAARGLAYLHDHCDPKIIHRDVKAANILLDEEYEAVVGDFGLAKLMDYKDTHVTTAVRGTIGHIAPEYLSTGKSSEKTDVFGYGVMLLELITGQRAFDLARLANDDDVMLLDWVKGLLKEKKLETLVDGDLQGNYVDAEVEQLIQVALLCTQSSPTERPKMSEVVRMLEGDGLTERWEEWQKEEMFRQEINLSHHPNTEWIVDSTSNLRPDELSGPR*
